# Challenges and Opportunities Arising from Host–*Botrytis cinerea* Interactions to Outline Novel and Sustainable Control Strategies: The Key Role of RNA Interference

**DOI:** 10.3390/ijms25126798

**Published:** 2024-06-20

**Authors:** Maria Spada, Claudio Pugliesi, Marco Fambrini, Susanna Pecchia

**Affiliations:** 1 Department of Agriculture Food and Environment, University of Pisa, Via del Borghetto 80, 56124 Pisa, Italy; 2 Interdepartmental Research Center Nutrafood “Nutraceuticals and Food for Health”, University of Pisa, Via del Borghetto 80, 56124 Pisa, Italy

**Keywords:** gray mold, plant protection, plant immunity, dsRNA, SIGS, HIGS, post-transcriptional gene silencing, RNAi-based fungicides

## Abstract

The necrotrophic plant pathogenic fungus *Botrytis cinerea* (Pers., 1794), the causative agent of gray mold disease, causes significant losses in agricultural production. Control of this fungal pathogen is quite difficult due to its wide host range and environmental persistence. Currently, the management of the disease is still mainly based on chemicals, which can have harmful effects not only on the environment and on human health but also because they favor the development of strains resistant to fungicides. The flexibility and plasticity of *B. cinerea* in challenging plant defense mechanisms and its ability to evolve strategies to escape chemicals require the development of new control strategies for successful disease management. In this review, some aspects of the host-pathogen interactions from which novel and sustainable control strategies could be developed (e.g., signaling pathways, molecules involved in plant immune mechanisms, hormones, post-transcriptional gene silencing) were analyzed. New biotechnological tools based on the use of RNA interference (RNAi) are emerging in the crop protection scenario as versatile, sustainable, effective, and environmentally friendly alternatives to the use of chemicals. RNAi-based fungicides are expected to be approved soon, although they will face several challenges before reaching the market.

## 1. Introduction

According to an estimation by the Food and Agriculture Organization of the United Nations (FAO), pests and pathogen infections lead to a yield loss that is about 20% to 40% of the potential annual total world crop production [1,2]. Food production systems still rely on chemical pesticides to maintain crop yields. However, the pervasive use of pesticides is a predominant cause of contamination for water, soil, and air, leading to the loss of biodiversity and pesticide resistance [3]. Moreover, human exposure to chemical pesticides is connected to the onset of chronic diseases such as cancer, heart, respiratory, and neurological ones [4].

Among the fungal pathogens, one of the most damaging for crops is *Botrytis cinerea*. This necrotrophic fungus, a causal agent of gray mold disease, results in significant losses in agricultural production, estimated to range from $10 to $100 billion per year [5]. The control of this fungal pathogen is rather difficult due to its wide host range and its environmental persistence. Currently, the management of gray mold disease is still mainly based on chemicals. However, the flexibility and plasticity of *B. cinerea* in challenging the defense mechanisms of plants, along with its ability to evolve strategies for escaping chemicals, require the development of new control strategies for proper management.

The use of RNA-based biopesticides as an alternative to the repeated application of conventional fungicides highlights the role of biotechnology in developing innovative and more environmentally friendly paths to achieve sustainable food security. For this reason, a new biotechnological tool based on the use of RNA interference (RNAi) is emerging in the crop protection scenario. RNAi is a process of post-transcriptional gene silencing (PTGS) triggered by molecules of double-stranded RNA (dsRNA), small interfering RNA (siRNA), or hairpin RNA (hpRNA), resulting in the specific degradation of target mRNA. In particular, the exogenous application of dsRNA against some pathogenic fungi has been reported as a non-genetically modified organism (non-GMO) strategy for plant disease control by targeting specific genes in a nucleotide-sequence-specific manner [6,7,8,9].

RNAi-based technology requires the fine-tuning of many factors to achieve effective pathogen control. In the design phase, a crucial step is to choose the target gene to be silenced while avoiding problems for non-target organisms. Moreover, other challenges for RNAi applications are represented by the amount of dsRNA produced and the stability of the formulation used. Due to the sequence-dependent nature of RNAi, dsRNA sequences can be customized to reduce the possible effects on non-target species. One of the possible strategies is to use highly specific genes of the pathogen, such as virulence genes, which are much less likely to have adverse effects on non-target organisms. Therefore, in view of the prospects of applying RNAi for pathogen control [Spray-induced Gene Silencing (SIGS), in vivo production of dsRNA and dsRNA-based formulations], the use of this type of gene is much more useful and less risky than using essential genes [10,11,12].

The aim of this review was to outline a detailed and updated picture of some aspects of host-*B. cinerea* interactions from which new and sustainable control strategies could be developed to minimize the use of classic synthetic fungicides. The control of this generalist and fearsome pathogen through increasingly ecologically sustainable solutions is an unresolved challenge that presupposes progress in research on various aspects such as the life span of the pathogen, disease cycle, virulence factors, host susceptibility traits, plant immunity mechanisms, hormones, and post-transcriptional gene silencing. All of these aspects have been addressed in this review, and their overview has represented our specific objectives.

Among the new and most promising unconventional control strategies against *B. cinerea*, the development of RNAi-based solutions is particularly interesting. Therefore, in this review, we focused our attention on the results obtained in different crops to control *B. cinerea* through the new solutions offered by RNAi. In this context, we paid particular attention to the prospects offered by exogenous dsRNA treatments. Additionally, some issues related to dsRNA production, formulation, and risks associated with the release of nucleic acids into the environment were addressed.

In summary, the different topics related to *B. cinerea* included in this review constitute an overview of the ongoing studies on this devastating pathogen, while the specific focus on RNA interference and dsRNA treatments to silence specific target genes allows us to understand where the research has reached and the future prospects of one of the most innovative control strategies in plant pathology.

## 2. *Botrytis cinerea*

*Botrytis cinerea*, belongs to the Ascomycota phylum, and is the causal agent of gray mold disease. Christian Hendrik Persoon described it for the first time in 1794 [13]. Later, Heinrich Anton de Bary [14] discovered that *B. cinerea* and *Botryotinia fuckeliana* represent the same fungus, the latter is the apothecial (sexual) stage of *B. cinerea*, while *B. cinerea* is the anamorph one [15].

**Figure 1:**
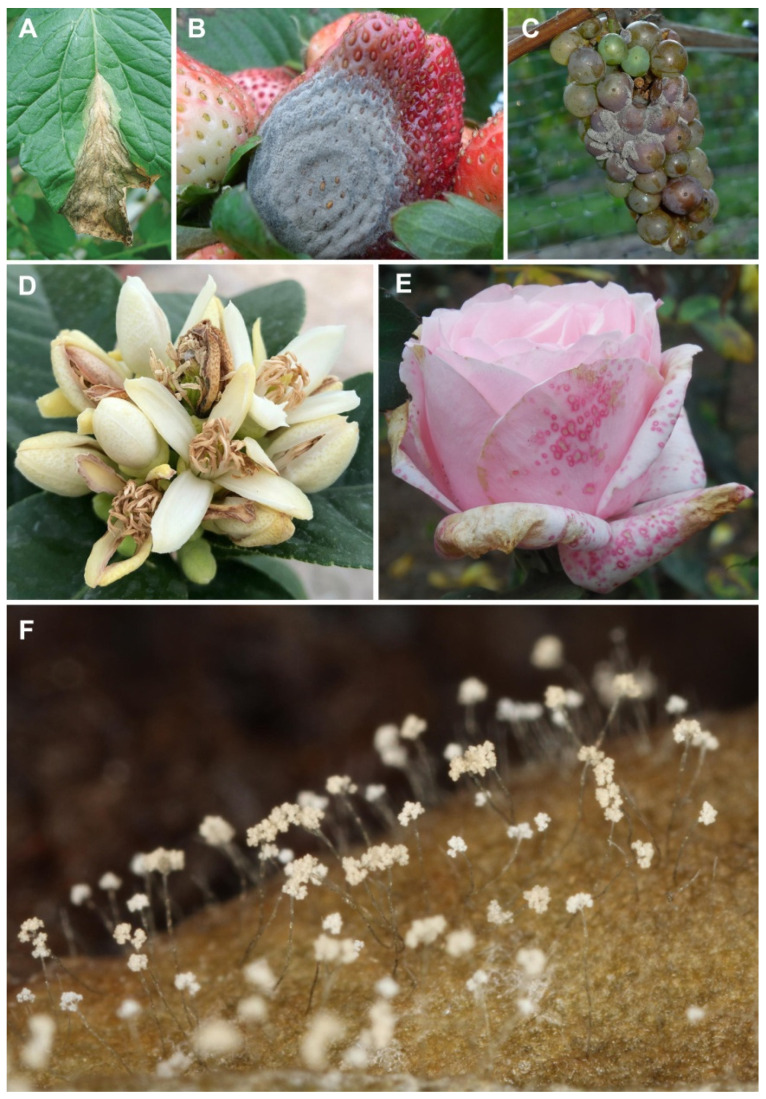
Symptoms of gray mold caused by *Botrytis cinerea*. (**A**) Leaf blight lesion on tomato (*Solanum lycopersicum*, L.), credit photo: K. Cullum, BioWorks, Inc., www.bioworksinc.com. (**B**) Fruit rot on strawberry (*Fragaria* × *ananassa* Duch), credit photo: Jonas Janner Hamann, Universidade Federal de Santa Maria (UFSM), Bugwood.org. (**C**) Bunch rot on grapevine (*Vitis vinifera* L.), credit photo: Gerald Holmes, Strawberry Center, Cal Poly San Luis Obispo, Bugwood.org. (**D**) Flower blight on Buddha’s hand citron (*Citrus medica* var. *sarcodactylus*), credit photo: Emilio Resta. (**E**) Petal blight on rose flower (*Rosa* L.), credit photo: Dr. Parthasarathy Seethapathy, Amrita School of Agricultural Sciences, Amrita Vishwa Vidyapeetham, Bugwood.org. (**F**) Conidiophores and conidia of *B. cinerea*, credit photo: David Cappaert, Bugwood.org (https://www.ipmimages.org).

*B. cinerea* is considered the major pathogen within the genus *Botrytis* [16,17], which includes 32 species that are commonly recognized as necrotrophic pathogens since they use enzymes to induce host cell death [18]. *B. cinerea* is considered a generalist pathogen, causing gray mold diseases in 586 genera of vascular plants [19,20]. *B. cinerea*, having a necrotrophic lifestyle, causes death of host cells with extensive damage to plant tissues, usually ending in rot of the plant or harvested product (Figure 1) [21]. *B. cinerea* is an outstanding pathogen due to its elastic infection approaches, high reproductive capability, wide host range, and aptitude to survive for prolonged periods as conidia and/or sclerotia. The pathogen is most damaging to senescent or mature plant tissues, but it can also attack them at early stages of development. Extensive damage is also caused by harvesting apparently healthy crops that are transported to markets, resulting in obvious post-harvest losses [22]. Due to its highly damaging nature and worldwide spreading, *B. cinerea* was ranked second on the top ten list of fungal pathogens for its economic and scientific importance [5]. Management of diseases caused by *B. cinerea* is rather complicated because of the pathogen’s tendency to develop rapid resistance to fungicides [23], and the use of a single control approach is unlikely to be successful [24,25]. As in the case of other fungal pathogens, the most pervasive method of controlling *B. cinerea* is through chemical approaches [26,27]. However, the availability of the genome of *B. cinerea* [23], which has been fully sequenced and recently improved in coverage and annotation [28], is a tool that provides a better understanding of the pathogen and represents a reservoir of opportunities for controlling its activity.

### 2.1. The Life Cycle of Botrytis cinerea

*B. cinerea* can live as micro- and macroconidia, chlamydospores, ascospores, mycelia, sclerotia, and apothecia. The life cycle of the pathogen includes an anamorph stage in which asexual spores (macroconidia) are released from mature conidiophores (Figure 1), while sclerotia are formed under unfavorable environmental conditions [29,30]. Under advantageous environmental conditions, sclerotia germinate, emitting conidiophores or, following a heterothallic sexual cycle, forming apothecia, which deliver ascospores [29]. Ascospores are produced through microconidia that fertilize sclerotia of different mating types; in any case, the sexual cycle is not very common in nature [22,31]. Many microconidia are observed in the sexual reproductive cycle, which constitute an additional microscopic propagule when *B. cinerea* is subjected to adverse conditions [32]. Microconidia can behave as spermatia during sexual reproduction and can develop from the germ tubes of macroconidia, within the bare cells of old hyphae, and from sclerotia and appressoria [32,33]. Chlamydospores are another temporary survival structure through which the fungus can overcome short-term adverse conditions and originate after a transformation of the vegetative mycelium and its hyphal disintegration [33]. Therefore, the fungus has a wide variety of overwintering structures and, likewise, various sources of inoculum that enable it to initiate infection. In addition, its remarkable flexibility allows it to survive in different habitats and in environments that are not always favorable. The combination of these unique characteristics explains why disease control caused by *B. cinerea* is so difficult to manage.

### 2.2. The Disease Cycle of Botrytis cinerea

**Figure 2:**
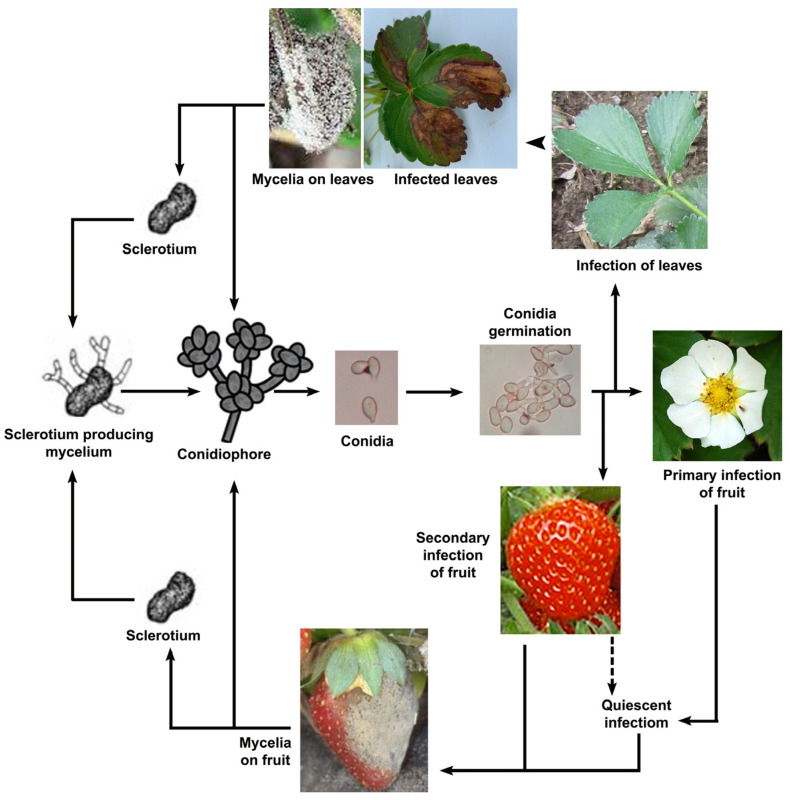
Disease cycle of *Botrytis cinerea* in strawberries. Modified by Petrasch et al. [35]. The photos of the conidia and their germination come from Spada et al. [36].

The disease cycle of *B. cinerea* begins with a conidium that lands on the plant surface (Figure 2) and attaches to it. After its attachment, it starts to germinate, producing a germ tube that will develop into an appressorium through which it penetrates the host surface [34].

The fungus begins its primary injury by killing the underlying cells, and host defense responses and necrosis occur at this time [34]. However, when the host defense system is bypassed, the fungus begins to grow intensely, leading to rapid maceration of plant tissues and even sporulation to produce the inoculum for the next infection [37].

#### 2.2.1. Conidia Attachment and Germination

The conidia of *B. cinerea* are pervasive in the air and can randomly end at any host [33,38]. They may also be delivered to host plant tissues by insects after their ingestion [39,40].

After conidia arrive on the host surface, adhesion to the epidermis occurs in two distinct phases. Rather weak adhesive forces, apparently involving hydrophobic interactions, define the first phase, which occurs after hydration of the conidia. The second phase of adhesion appears when the conidia have already been incubated for many hours under conditions favorable for germination [41]. At this point, germlings can actively adhere to both hydrophobic and hydrophilic substrates [42].

Once landed and attached to the surface, the conidia start to germinate. Many factors can influence conidia germination. First, a very high level of relative humidity is fundamental for germination and must be higher than 93% [43,44]. Moreover, the availability of free surface water plays a crucial role in germination. In fact, during plant surface penetration, its absence could cause the formation of a shorter emerging germ tube [44,45]. The availability of sugars (glucose, xylose, and galactose) at this stage seems to be important not only in enhancing germination but also involved in oxidative processes connected to host cell death [46]. Another factor that could influence germination is the presence of ethylene, which is usually produced by the plant at the time of tissue senescence or fruit ripening. A necrotroph such as *B. cinerea*, since it feeds on dead plant cells, could take advantage of this signal that stimulates conidia to germinate on the hydrophobic host surface and to start the infection [47,48]. Ethylene might have a dual task during infection: weakening the host and promoting conidia germination and hyphal growth [34].

#### 2.2.2. Appressorium Formation and Role of the *BcPls1* Gene

**Figure 3:**
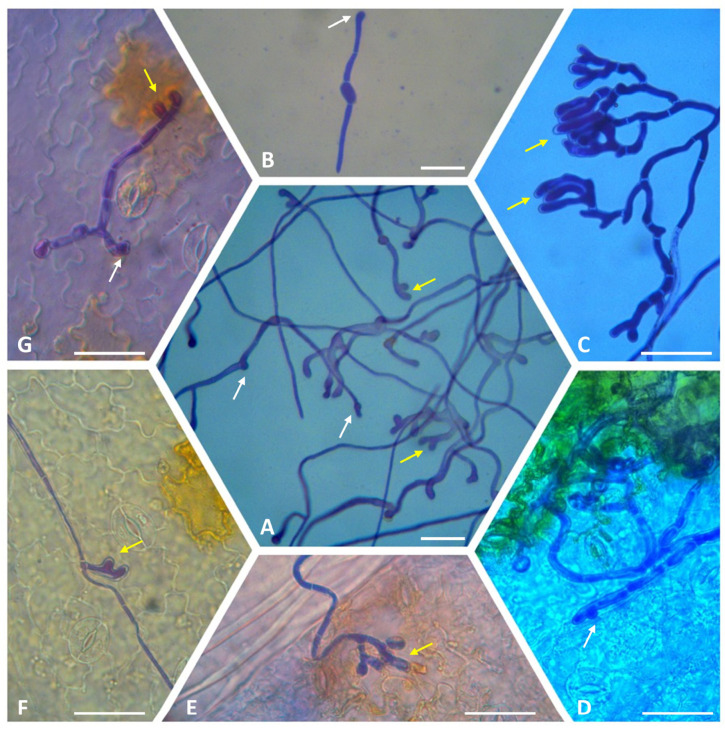
Formation of appressoria and infection cushions by the *B. cinerea* B05.10 isolate. Conidial suspensions in SMB medium were inoculated onto glass slides or sprayed onto the surface of lettuce plants [63]. Glass slides were prepared as described by Hou et al. [64] and stained with trypan blue or lactophenol cotton blue after 24 h (**A**,**B**) or 72 h (**C**) of incubation. Artificially inoculated lettuce plants were incubated in transparent plastic propagation boxes at 25 ± 1 °C with 98–99% relative humidity under natural lighting conditions in the laboratory for 6 days. Infected leaf discs (Ø 6 mm) were fixed, cleared, and stained with lactophenol cotton blue (**D**,**E**) or trypan blue (**F**,**G**) as described by Isenegger et al. [65]. White arrow = appressorium; yellow arrow = infection cushion. Light microscopy was performed with a Dialux 22 microscope (Leitz, Wetzlar, Germany). Images were captured using a Leica DFC 450C digital microscope camera with control software LASX Version 3.1.1.17751 (Leica Microsystems Ltd., Heerbrugg, Switzerland). Scale bars = 25 µm.

To finalize the host penetration*, B. cinerea* starts to form infection structures, and the germ tube, once it reaches lengths of 10–15 µm, differentiates into an appressorium [33] (Figure 3). The appressorium adheres to the host surface redirecting, the polarized growth to 90 °C and forming a penetration peg, which can break the host cuticle [45,49,50]. The germ tube forms a simple unicellular appressorium within 6 h after germination, which is a hyaline and lightly swollen germ tube apex adhering to the host. When exogenous nutrients are available, at least 12 h after germination, multicellular dome-shaped appressoria can be formed, referred to as infection cushions [33,51,52,53] (Figure 3). The hyphal tip of the germ tube of *B. cinerea*, after an increase in osmotic potential, begins to swell and retain water.

Gourgues et al. [54] identified the *BcPls1* gene in the genomes of *B. cinerea*, *Colletotrichum lindemuthianum*, and *Neurospora crassa*, homologous to the *Pls1* gene of *Magnaporthe grisea*. In *M. grisea*, this gene encodes for a tetraspanin required for penetration and essential for appressorium function [55]. Tetraspanins are small proteins associated with membranes and were first identified in mammals in multiple copies [56]. Their function appears to be as adaptors of membrane proteins activated in cell differentiation, motility, and adhesion [57,58,59]. Gourgues et al. [60] showed that *BcPls1* was expressed immediately before and during penetration of *B. cinerea* in appressoria, germ tubes, and conidia, but it was no longer detectable at 18 h post-infection, corroborating the hypothesis that *BcPls1* is needed for the penetration of *B. cinerea* into intact host plants. In addition, they pointed out that functional appressoria are necessary for the successful penetration of *B. cinerea*. In fact, *B. cinerea BcPls1*-deficient null mutants (*Bcpls1::bar*) generate an apparently normal appressorium but cannot direct the penetration peg toward the host surface, instead differentiating secondary hyphae that grow on the surface without penetrating. *Bcpls1::bar* mutants were not able to infect intact tissues but were still able to infect injured plant tissues, suggesting two different pathways, one dependent and the other independent of *BcPls1* [59].

Moreover, the lack of penetration for *BcPls1* mutants could be explained by the inability to establish polar growth at the base of the appressorium orthogonally to the host surface [61].

#### 2.2.3. Active Penetration of the Host

When *B. cinerea* attacks the host, it can choose between an active penetration and a passive one. In the last case, it can use natural wound sites, entering through open stomas or also by artificial openings due to previous infections operated by other pathogens.

Concerning active penetration, when conidia land on host tissues, the real first barrier they meet is the cuticle covering the epidermal cells, since the wax layer does not seem to be a real obstacle. In fact, *B. cinerea* can reduce surface hydrophobicity and overcome the wax layer by using the polysaccharide cinerean as a surfactant [62].

When the penetration peg cracks the cuticle, it can generate H_2_O_2_ [66], which could help penetration by providing a substrate for oxidases that tweak the cuticle. During cuticle penetration by appressorium, a superoxide dismutase *BcSOD1* is secreted, and it may contribute to the process since it was demonstrated that ∆*bcsod1* mutants had a lower virulence on different hosts [67].

After passing through the cuticle, the penetration peg of *B. cinerea* invades the anticlinal wall standing between two epidermal cells (rich in pectin), and concurrently, the swelling of their cell wall occurs [68]. This suggests that a pectin degradation process is involved during host penetration; in particular, the endopolygalacturonase (endoPG) BcPG2 seems to regulate this process. *Bcpg2* gene deletion mutants exhibit a consistent delay in primary lesion formation, and their virulence is greatly reduced in tomato and bean [69], although mutants in several *endoPG* genes did not have the same strong effect on necrotizing activity.

### 2.3. Botrytis cinerea: A Killer Pathogen

*B. cinerea* has always been classified as a necrotrophic pathogen, although recently a classification as hemibiotrophic was proposed since it has a short biotrophic phase of 8–16 h at the very beginning of infection [70], but in any case, it remains a model organism among fungi with a necrotrophic lifestyle.

Many metabolites and proteins can be produced by *B. cinerea* to cause cell death or to induce symptoms of programmed cell death (PCD) that the fungus takes advantage of for plant invasion [47,71]. Among the secreted secondary metabolites, two main compounds with phytotoxic activity have been identified: a bicyclic sesquiterpene called botrydial and a highly substituted lactone called botcinolide [72,73,74].

*B. cinerea* can also produce oxalic acid (OA), which is not considered a primary phytotoxic agent but rather a co-factor in pathogenesis [65]. In fact, low pH levels enhance the synthesis and activity of aspartic protease, endoPG, and laccase [75]. In addition, OA stimulates pectin degradation after endoPG activity. When OA subtracts the Ca^2+^ ions of Ca-pectates present in the cell walls, it negatively acts against the intermolecular forces between pectic polymers, helping to break down the pectin structure, which therefore swells because of water absorption [68].

The reactive oxygen species (ROS) are also involved in the attack strategy of the fungus; in fact, they are produced at the host-fungal interface. The presence of O_2_^−^ and H_2_O_2_ in hyphal tips or close to the invaded host cell wall was demonstrated [76]. Usually, in plant-pathogen interactions, the oxidative burst appears during a hypersensitive response (HR), a PCD aimed to stop the availability of water and nutrients for the fungus, and that gives resistance to biotrophic pathogens [77].

In the case of *B. cinerea*, being a necrotroph, plant cell death is profitable to the pathogen [47]. During cuticle penetration and primary lesion formation, the fungus actively provokes an oxidative burst [76,78]. In ROS production, nicotinamide adenine dinucleotide (NADPH) oxidases are enzymes that play a key role in *B. cinerea* virulence as they generate O^2−^, which is further converted to H_2_O_2_ by a superoxide dismutase (BcSOD1) [79].

Two genes involved in the pathogenicity of *B. cinerea* and encoding two NADPH oxidases have been studied: *BcnoxA* and *BcnoxB*. Mutants for these genes showed a reduced virulence, proving that *BcnoxB* was involved in penetration and *BcnoxA* in lesions spreading. Moreover, a double knockout *ΔbcnoxAB* mutant was approximately avirulent [79].

Among the metabolites and proteins secreted by the fungus and capable of causing PCD are those that induce HR symptoms. For example, NEP1-like proteins associated with the cell membranes of the responsive cell can reach the nuclear envelope of the responsive cell [80]. BcNEP1 and BcNEP2 belong to NEP1-like proteins and can increase callus apposition, ROS accumulation, and the induction of defense genes to the point of causing cell death when present at high doses [81].

Another family of proteins that can induce HR symptoms is the cerato-platanin family. In particular, the BcSpl1 protein is one of the most abundant in this family. It has been shown to induce the hypersensitivity response in the host plant, including ROS induction, cytoplasmic shrinkage, electrolyte leakage, cellular autofluorescence, and activation of defense genes [82].

#### 2.3.1. From Living Plant Tissues to Fungal Biomass

The main goal of *B. cinerea* is to convert plant biomass into fungal biomass; therefore, the fungus synthesizes a plethora of cell wall degradation enzymes (CWDEs), whose main substrate is pectin. For example, pectin lyase, pectin methyl esterase, and many polygalacturonases (PGs) show a preference of *B. cinerea* toward hosts with a high amount of pectin in the cell wall [83,84,85]. Pectin methylesterases (PME) seem to help in the depolymerizing of highly methylated pectin, making it easier to be degraded by pectate lyases and PGs [86]. In the strain Bd90, a mutant for the gene *Bcpme1* encoding PME was less virulent, even if in the strain B05.10, mutants in two *Bcpme* genes included *Bcpme1* and did not show any reduction in virulence. A possible explanation is that the strain B05.10 can avoid demethylation for pectin degradation [86,87].

In the process of pectin degradation, a key role is played by endoPGs. In the genome of *B. cinerea*, there are at least six genes encoding for endoPGs, whose expression may vary depending on the stage of infection and host physiology [88]. *B. cinerea* also produces exoPGs that catalyze the hydrolytic cleavage of galacturonan, thus making low molecular mass and easier-to-metabolize compounds available to the fungus [89].

Among the CWDEs, the fungus produces cellulases that degrade the cellulose at the β-1,4-glycoside bonds, although when the cellulase gene is deleted, it does not compromise fungal virulence. In fact, Cel5A, an endo-β-1,4-glucanase, is not needed in pathogenesis, even if it is expressed during infection [90].

*B. cinerea* also produces xylanases that break the β-1,4-polysaccharide bonds of xylan, the main hemicellulosic component of the host cell wall. A knockout mutant for the *Xyn11A* gene had a wide effect on pathogenicity, with a reduction in the lesion size of 70%.

Proteases are another important class of enzymes for *B. cinerea*; in fact, thirty-four different proteases have been found experimentally in its secretome [91]. First, proteases generate amino acids that promote fungal growth, and second, they may cooperate in the process of plant cell wall degradation. The aspartic protease (AP) BcAP8 is produced by *B. cinerea* immediately after germination and accounts for about a quarter of the protein mass [92]. Mutants for six aspartic proteases have been generated, and only the *Bcap8* mutant showed a 70% lower protease activity, but no difference in virulence was observed [93].

However, it is worth highlighting that specific changes in cell wall compositional profiles appear to correlate with fungal disease susceptibility. In some wine and table grape cultivars, hemicellulose layers and the arabinogalactan protein content were largely unaffected by *B. cinerea* infection. Cell wall factors important in influencing resistance may include pectin methylesterification profiles as well as extensin reorganization [94].

#### 2.3.2. Tissue Invasion and Host Response: A Cross Talk

*B. cinerea* is programmed to kill the host plant using a double strategy: on one side, necrotic cell death, and on the other, the host PCD. Its penetration of the host surface and the enzymatic breaking of plant cell walls stimulate a cascade of events both in the fungus and in the host [91,95,96].

Cell wall appositions (CWA), such as papillae, are post-infection defense mechanisms developed by plants in response to pathogen attack. Numerous studies have focused on the characterization of papillae by ultrastructural observations or histochemical analyses. Several classes of compounds are associated with papillae: callose, phenolic compounds including lignin, reactive oxygen species (ROS), cell wall structural proteins such as arabinogalactan proteins, and cell wall polymers. In particular, callose acts as a physical barrier but also serves to protect the plant cell from toxic metabolites that accumulate in papillae [97].

The accumulation of callose and suberin in young tomato fruit has been linked to the formation of “ghost spots” (small necrotic lesions surrounded by a white halo), which appear to limit *B. cinerea* growth [98,99].

Callose deposition also occurs after infection with mycorrhizal fungi and plant growth promoting bacteria. Recently, a study showed that tomato plants inoculated with *R. irregularis* induced higher levels of callose and displayed smaller mycelium diameters during infection with *B. cinerea*. Knowledge of the factors involved in the regulation of callose during these microbial interactions could be useful in the development of new biotechnological approaches for crop protection [100].

At the host-pathogen interface, the two pathways of autophagy and apoptosis are in a dynamic equilibrium between them. The ability of *B. cinerea* to suppress autophagy could be crucial for disease progression as much as that of inducing apoptosis in host plants [74,101]. The breakdown of autophagy gives the fungus time to grow within the plant, and when fungal biomass is optimal, the fungus switches to producing compounds that initiate apoptosis, causing cell death and necrotic tissue. In this way, instead of wildly killing the host, the pathogen pilots the plant to colonize it [74].

First, considering that the main cause of *B. cinerea* infections are conidia, it is reasonable to think that there is not a massive secretion of killing substances by the fungus in the early infection phase [91]. Germinating conidia are exposed to toxic substances produced by the host to kill fungal cells and to destroy most of the new hyphae (e.g., camalexin) [102]. During this critical phase, the fungus must counteract host-induced cell death and use an anti-apoptotic mechanism to do so. This allows the necrotroph to retain a small number of viable fungal cells within the necrotic zone [102,103].

During the early stages of infection, host plants activate the autophagy pathway as a controlled mechanism of cell death to avoid the spread of necrotic areas and produce phytoalexins that actively kill the pathogen. This is a local cell death strategy to avoid an extensive one [74,101,104]. *Autophagic-related* (*atg*) genes are expressed during the host response against *B. cinerea*, and their knockdown leads to a rapid spread of necrotic tissues. This kind of response occurs not only in cells surrounding the infected area but also in distal areas in the absence of mycelium [101,104].

The maintenance of the infectious process depends on the establishment of a complete necrotic region before the pathogen is killed by toxic host metabolites [91]. The onset of primary necrotic lesion formation concurs with the activation of the host defense system in neighboring tissues as a reaction to the death of invaded cells [50].

During infection, the host may also produce a whole series of molecules that are typically synthesized as a defense response to HR caused by a biotrophic pathogen. The host implements the deposition of lignin, the secretion of phytoalexins, and pathogenesis-related (PR) proteins [105,106,107]. These mechanisms are activated at the level of primary necrosis, where the fungus is temporarily confined. Therefore, all mechanisms activated by the plant may give rise to a period of quiescence for *B. cinerea* [21]. During quiescent infections, there are no visible disease symptoms, and this is the case, for instance, for non-green tissues of soft fruits such as strawberries or grapes [37]. The fungus resumes to grow in correspondence with fruit ripening [37], and an explanation was found in the decreasing level of resveratrol, which is gradually lower during the ripening stage of fruits [108]. Resveratrol is a stilbene phytoalexin, a class of molecules with fungitoxic or fungistatic action produced as a defense mechanism by the attacked host plant. In immature fruits, along with phytoalexin, it is also possible to find inhibitors of PG called PG Inhibiting Proteins (PGIPs), which contribute to the quiescence phase of *B. cinerea* [109]. Several approaches using plant and *B. cinerea* mutants as well as transgenic plants have identified processes that target the plant cell wall via enzymes produced by both the host and the fungus [110,111].

#### 2.3.3. Evasion of Chemical Defense and Infection Spreading

Phytopathogenic fungi have developed numerous mechanisms over time to overcome the chemical barriers posed by their hosts. The main strategy is the enzyme detoxification of toxic compounds [112], which plays a key role in the success of host colonization [113]. The most studied example is the use of a stilbene oxidase, which is a substrate-specific laccase used to detoxify the phytoalexins resveratrol and pterostilbene and which allows the fungus to oxidize these compounds into harmless configurations [114]. *B. cinerea* can also detoxify other compounds such as α-tomatine and other saponins such as digitonin, avenacin, and avenacosides that are deglycosylated using three different saponinases: a xylosidase (tomatinase, digitoninase) and two glucosidases (avenacinase/avenacosidase) [115,116,117].

Moreover, because at the host-pathogen interface, the oxidative burst can stress not only the plant but also the fungus, the latter needs to detoxify the ROS produced using an extracellular catalase [118].

Once the primary lesion is established, the fungus begins to extend it by killing neighboring cells. It can use all CWDEs: pectin lyase, pectin methylesterase, cellulase, and exo- and endo-polygalacturonase. In this way, the fungus can shred plant tissues and convert them into fungal biomass, leading to the spread of the disease [21,119].

### 2.4. Signaling in Botrytis cinerea Regulates the Infection Process

*B. cinerea* is well known for its wide host range, and this ability may depend on its evolved strategies to identify the proper host, penetrate and colonize its tissues, and overwhelm the host’s defense response. To do so, the fungus must pick up physical and chemical signals from the different hosts and, in the meantime, react with appropriate means that promote fungal development. The fungus undergoes a series of metabolic transformations that lead to adhesion of conidia to the host surface, differentiation of the germ-tube and its oriented growth, the formation of infectious structures, and the synthesis of enzymatic and phytotoxic compounds [120]. All these mechanisms need a network of signal transduction mechanisms, which includes G protein activity, cyclic adenosine monophosphate (cAMP) signaling, and the mitogen-activated protein kinase (MAPK) cascade pathway. The external signal activates the related genes in the fungal genome, aiming to realize the appropriate response and establish the infection [121,122,123].

#### 2.4.1. The Role of G Proteins in *Botrytis cinerea* Pathogenesis

Guanine nucleotide-binding proteins, or G proteins, are GTPases of the Ras superfamily and can be divided into monomeric and heterotrimeric. Monomeric G proteins are important regulators of many biochemical reactions that depend on their transition from an inactive form, with GDP-to-GTP exchange promoted by guanine nucleotide exchange factors (GEFs), to an activated configuration [124]. In their active form, they can interact with effector proteins by transmitting the signal to downstream pathways [124,125]. *B. cinerea* owns three RasGTPases involved in cell proliferation; in fact, knockout mutants of *Bcras1* or *Bcras2* are affected in hyphal growth and are impaired in radial growth rates, respectively [126].

The fungus also contains a protein belonging to the RhoGTPase family, usually involved in cell morphology/cytoskeleton dynamics, named BcRac. *Bcrac* mutants are quite such as *Bcras1*, perhaps because the two monomeric GTPases play a role in the same signaling mechanism [127]. The heterotrimeric G proteins can regulate multiple cellular functions. They are signal transducers that pair receptors from the cell surface to cytoplasmic ones, playing an essential role during pathogenic development [121]. These proteins were named by their ability to bind guanine nucleotides and contain three different subunits, α, β, and γ, where Gα is the nucleotide-binding one [128,129]. The Gα subunit can take on different conformations depending on whether it is bound to GDP or to GTP [121].

The G protein subunits, in their inactive configuration, are bound to the G protein-coupled receptor (GPCR), which crosses the membrane. When a ligand binds to the receptor, there is a dissociation between Gα-GTP and the Gβγ dimer for a GTP exchanged with a GDP in the Gα. At this point, the subunits Gα and Gβγ can form bonds with effectors localized on the membrane, such as adenylate cyclase, phosphodiesterases, phospholipases, and ion channels. The Gα subunit has intrinsic GTPase activity; consequently, after the hydrolysis of the bound GTP, there is a re-link of Gβγ, GPCR, and Gα-GDP [130].

*B. cinerea* has three genes coding for the Gα subunit named *Bcg1*, *Bcg2*, and *Bcg3*, and they are all involved in the infection process. Gα1 (*Bcg1*) appears to be involved in the colonization of plant tissues immediately after penetration, and when inactivated, infected leaves cannot be observed to spread lesions [131].

Gα3 (*Bcg3*) appears to be important both for conidia germination when carbon sources are available and for primary lesion formation. In fact, the penetration of ∆*bcg3* conidia was shown to be less efficient, and the reduced penetration rate could be due to defective host surface sensing, which resulted in fewer penetration attempts [132].

When *Bcg3* is inactivated, there is a lower conidiation, a higher sclerotia formation, and a delay in tomato leaf infection due to a delayed establishment of primary necrosis [132]. Moreover, Gα1 and Gα3 can elicit cAMP production by triggering the adenylate cyclase activity [132,133].

*B. cinerea* also has two Gβ and Gγ genes, *Bcgb1* and *Bcgg1*, working as functional units to maintain the inactive form of the Gα subunit [134].

G protein signaling can be directed by some regulators (RGSs) that trigger Gα subunits to behave as GTPase-activating proteins (GAPs). Among the possible RGSs for *B. cinerea*, phosducin-like proteins (PhLPs) can be found [130,135]. They are fundamental for correct G protein signaling since they work as chaperones in the association of Gβγ dimers [135]. *B. cinerea* possesses three PhLPs, and it appears that BcPhnA is essential for plant infection as it is involved in the production of sclerotia and conidia [23,124].

Among the possible G protein-coupled receptors (GPCRs) in *B. cinerea*, BcGpr3, belonging to the cAMP receptor-like class (BcGpr2-6), seems to be involved in giving resistance to the defensin VvAMP2 produced by grapevine [23,134,136].

#### 2.4.2. The Cyclic AMP (cAMP)-Dependent Pathway Affects *Botrytis cinerea* Pathogenesis

The cAMP-dependent signaling pathway can regulate many fungal processes in plant pathogenic fungi, such as virulence, differentiation, and morphogenesis. For example, in *M. grisea*, a cAMP signaling pathway is involved in appressorium formation; in fact, mutants for *adenylate cyclase* (*MAC1*) failed to penetrate becoming no more pathogenic [137]. The adenylate cyclase (AC), a membrane-associated enzyme, is the main effector protein of heterotrimeric G proteins, and it is activated by the interaction with Gα subunits after the messenger cAMP is formed by the ligand bound to the GPCR. Generally, PKA, a cAMP-dependent protein kinase, is the primary receptor for cAMP in eukaryotes [138]. *B. cinerea* contains genes coding the PKA (*BcPkaR*), two genes encoding its subunits (*BcPka1*, *BcPka2*), and the adenylate cyclase (*Bac*) [126].

In *B. cinerea*, the adenylate cyclase gene *Bac* is expressed from the onset of necrosis development until its spread from primary necrotic spots. A mutation in the *Bac* gene led to no conidia formation and reduced colony growth and virulence. Moreover, the interaction of *Bac* with Gα1 and Gα3 elevates cAMP at the intracellular level [132,139]. cAMP abundance is also regulated by the main catalytic PKA subunit, which is *BcPka1*, and, when it is deleted, there is remarkable growth retardation, virulence reduction, and a higher cAMP amount [126]. Δ*bac* and Δ*bcpka1* mutants showed quite similar reduced virulence, but the lack of germination and sclerotia formation induced by sugar are very unique features for Δ*bac* suggesting other possible effectors of *Bac* and cAMP signaling [124,126].

The cAMP pathway is also regulated by phosphodiesterases (PDEs), since they deteriorate the secondary messenger. Among PDEs, BcPde2 is important for the appropriate regulation of sclerotia formation, conidiation, colony growth, and virulence. Differently from other fungi, where the absence of PDE activity causes higher cAMP levels along with highly active PKA signaling, for *B. cinerea*, it leads to marginally lower cAMP and PKA activation [140].

#### 2.4.3. The Role of Mitogen-Activated Protein Kinases (MAPKs) in *Botrytis cinerea*

The mitogen-activated protein kinases (MAPKs) have a crucial role in the transduction of extracellular signals by using phosphorylation/dephosphorylation cycles to flow information [124].

**Figure 4:**
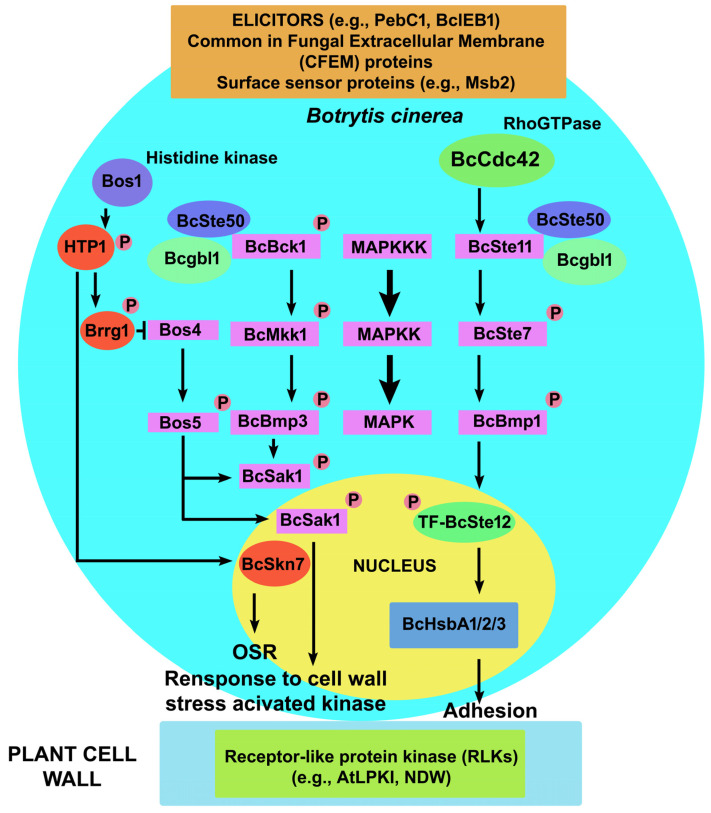
General outline of a MAPK pathway in *Botrytis cinerea*. Signals from *B*. *cinerea* elicitors, extracellular membrane (CFM) proteins, and surface sensors (Msb2) are transferred to the central component of the MAPK cascade. The MAPK pathway consists of MAP kinase kinase kinase (MAPKKK), MAP kinase kinase (MAPKK), and MAP kinase (MAPK). The MAPK pathway is operated by three modules via BcBmp1 (BcSte11-BcSte7-BcBmp1), BcBmp3 (BcBck1-BcMkk1-BcBmp3), and BcSak1 (Bos4-Bos5-BcSak1) in response to osmotic and oxidative stress. The signal transduction pathway functions through Bos1, which activates a His-phosphotransferase (HPT) protein, and two response regulators, Brrg1 and BcSkn7, involved together with BcSak1 in the osmotic stress response (OSR) pathway. Also indicated are the putative MAPK adaptor protein BcSte50 and a member of the acyl-coenzyme A synthetase family, Bcgbl1, that positively regulates BcSte50-mediated phosphorylation of BcBmp1. In the nucleus, the transcription factor BcSte12 activates the hydrophobic surface-binding protein BcHsbA1/2/3 for adhesion to host tissues and virulence. Kinases activated by cell wall stress and virulence signals are sensed through membrane receptor-like protein kinases (RLKs) of the host cells. Modified by Sharma and Kapoor [153] and Tang et al. [154].

MAPK cascade is formed by three interlaced protein kinases that are serially activated through phosphorylation of Ser, Thr, and Tyr residues: the MAP kinase kinase kinases (MAPKKKs), the MAP kinase kinases (MAPKKs), and the MAP kinases (Figure 4). After their activation, MAPKs phosphorylate the effector proteins located in the cytosol or in the nucleus. In this way, MAPKs trigger very specific downstream responses, such as the activation of the expression of gene arrays and transcription factors in response to environmental stimuli [141].

Sequence comparisons with MAPK modules present in *Saccharomyces cerevisiae*, which regulate cell wall integrity, hyperosmoregulation, invasive growth, mating, and ascospore formation, showed the presence of three MAPKs in *B. cinerea* [141,142].

The *Fus3p*/*Kss1p* ortholog is *BcBmp1*, a single-copy gene homologous to the *PMK1* of *M. grisea*, which is crucial for invasive growth and appressorium formation [143]. The *PMK1* homologue was also studied in *Colletotrichum lagenarium* and *Cochliobolus heterostrophus*, and it was demonstrated to be essential for pathogenesis [144,145]. For all these three phytopathogenic fungi that form appressorium during infection, their mutants for *PMK1* or its homologues, appressorium formation, were lost [143,144,145].

*BcBmp1* deletion mutants in *B. cinerea* were non-pathogenic in carnation flowers or on tomato leaves, and they were faulty in regulation of the penetration process and in eliciting plant cell death. Even if conidia did not show irregularity in germination and continued to grow on plant surfaces, they could not penetrate or cause necrotic lesions. Concerning fungal growth or conidiation, *Bmp1* seemed to be not essential but useful in maintaining appropriate mycelial growth rates. In fact, *B. cinerea bmp1* mutants did not show defective germination of conidia, but anyway, they showed reduced vegetative growth. In other fungi, the *PMK1* homologues play many different roles in fungal growth and differentiation, as well as in the regulation of plant infection [146].

Moreover, it was highlighted that *BcBmp1* regulates conidia’s ability to intercept different signals, such as chemical ones provided by nutrients and physical ones given by the host surface. *BcBmp1* is partially necessary for germination signaling triggered by carbon sources, along with cAMP and BcG3. In fact, source-induced germination is affected in the ∆*bmp1* mutant, even if the effect is not comparable to the ∆*bcg3* one. Furthermore, *BcBmp1* is crucial for signaling induced by hydrophobicity; in fact, ∆*bmp1* conidia had almost no germination on the hydrophobic surface [132].

Among the several effector proteins of *BcBmp1*, *BcSte12* was studied, and mutants with deletion of this transcription factor showed a delayed infection due to low penetration efficiencies, no sclerotia development, and intensified melanization. These findings support the hypothesis that during germination, *BcBmp1* and *BcSte12* regulate a wide number of genes [147].

The yeast *Slt2* ortholog is the *BcBmp3* gene [148] for *B. cinerea*. In filamentous fungi, *Slt2*-type MAPK seems to be involved in preserving cell-wall integrity and to be related to some aspects of saprotrophic and pathogenic growth [149,150,151,152]. In *M. grisea*, the *S. cerevisiae Slt2* homologue is *MPS1*, which is important for maintaining cell wall integrity and for appressorium penetration. *MPS1* is also involved in conidiation and aerial hyphae development [152]. In *C. lagenarium*, the ortholog *MAF1* is involved in many steps of the infection process and is important in the initial differentiation stage of appressorium formation [150].

*BcBmp3* was demonstrated to be involved in the saprotrophic growth of *B. cinerea*, and ∆*bmp3* mutants had only scant aerial mycelium with a reduced number of conidiophores and few macroconidia produced in favor of microconidia. Uniquely, compared to other fungi, there was no loss of cell wall integrity or increased sensitivity to CWDEs in *B. cinerea bmp3* mutants. On low-osmolarity media, radial growth is reduced, but the same was not observed in germlings, as this response was dependent on developmental stage [155]. *Slt2*/*Bmp3* seems to be involved in the stabilization of internal osmotic pressure, in a way complementary to the *Hog1* gene in *S. cerevisiae*, known for its role in the response to high osmolar conditions [155,156]. *BcBmp3* also seems to be involved in surface sensing of hyphae, which is an essential pre-penetration event. ∆*bmp3* mutant germlings had germ tubes with excessive elongation, and mostly superficially growing hyphae with a consequently reduced penetration rate [155]. This would explain why mutants showed growth retardation and a delay not only in primary lesion formation but also in lesion expansion. Moreover, Δ*bmp3* is defective in the formation of sclerotia with a similar trend of Δ*bcnoxA* mutants [79,155], consistent with the *BcBmp3* positive regulation of the *BcnoxA* gene. As suggested by Segmüller et al. [79], the MAPK cascades have an impact on the *BcNox* gene expression in *B. cinerea*. It seems that *BcBmp1* negatively modulates *BcNoxA* expression, and even greater is the control of *BcBmp3*, which is positive on *BcNoxA* expression and negative on *BcNoxB* expression. Thus, *BcBmp3* seems to play an essential function in the *bcnox* genes regulation at transcriptional level.

The ortholog of the yeast *Hog1* is *BcSak1* in *B. cinerea* [157]. In several pathogenic fungi, such as *C. lagenarium*, *M. grisea*, and *Cryphonectria parasitica*, *Hog1* homologues and their role in pathogenicity have also been studied, but they are generally involved in response pathways to oxidative and osmotic stresses [158,159,160]. *BcSak1* showed instead to have unique features since, in contrast to other studied fungi, it has a significant impact on pathogenesis for *B. cinerea*. In fact, it is important for conidia and sclerotia formation. The *bcsak1* mutants had a complete absence of conidiation and developed sclerotia earlier compared to the wild type. Mutants were defective in the early phases of penetration as they grew very slowly with long and mostly unbranched hyphae, without forming appressoria, and without penetrating the plant [157].

Among the regulating elements that are upstream of the MAPKs cascade, in *B. cinerea* there is a phosphotransfer histidine kinase (HK) acting as a response regulator (RR) and belonging to the family-III HK, called Bos1 [161]. The *Bos1* gene product is involved in osmoregulation pathways, negatively regulating the phosphorylation level of its downstream BcSak1 [161,162]. Deletion of *bos1* leads to constitutive activation of *BcSak1*, with a higher sensitivity to hyperosmotic conditions and oxidants and a lower one to fungicides. It also leads to impaired virulence and loss of conidiation [157,161,163].

*BcSak1* was shown to be involved in hyperosmotic stress in the presence of NaCl and in the oxidative stress response caused by H_2_O_2_, with visible effects on the mutants where no evident growth was detectable or it was significantly reduced, respectively. In fact, the protein is phosphorylated following hyperosmotic and oxidative stressful conditions as well as after treatment with calcofluor white (CFW) and fungicides, confirming a role in cell wall composition and integrity [157,161].

Mutants of *B. cinerea* lacking *BcSak1* showed higher contents of chitin and β-1-3 glucan, with a lower susceptibility to CWDEs and cell wall interfering agents, along with the resulting quantity of glycerol after fungicide and NaCl treatments. Furthermore, in the *Bcsak1* mutant, *BcBmp3* is hyperphosphorylated in the presence of oxidative stress, demonstrating that both MAPK pathways interact between them. In addition, all the MAPKs seem to regulate melanin biosynthesis: *BcSak1* and *BcBmp3* are indispensable for pigmentation, and *BcSak1* is particularly essential after light exposure, while *BcBmp1* is fundamental for pigmentation in dark conditions [164].

## 3. Further Aspects Related to Plant Immunity and an Overview of Possible Unconventional Defence Strategies against *Botrytis cinerea*

In plant immunity, two levels of responses are induced. In the first instance, plant trans-membrane pattern-recognition receptors (PRRs) can identify microbe-, pathogen-, and/or damage-associated molecular patterns (MAMPs, PAMPs, and/or DAMPs). This type is indicated as PAMP triggered immunity (PTI). In this case, very frequently is, for example, the generation of reactive oxygen species (ROS), the production of phytoalexins, and the activation of mitogen-activated protein kinase (MAPK) signaling pathways, with consequent induction of gene transcription providing protection against pathogens. When the level of PTI is exceeded, the host can activate a second level of cell-dependent response through specific resistance (R) proteins characterized by conspicuous leucine-binding nucleotide domains. R proteins recognize effectors, which are pathogen-delivered proteins called effectors. Hence, the second level of perception involves the recognition by intracellular receptors of pathogen virulence molecules called effectors; this recognition induces effector-triggered immunity (ETI) [165,166,167,168].

In general, the understanding of the molecular mechanisms underlying the control of plant responses to *B. cinerea* has great relevance from an application point of view in facilitating the development of environmentally low-impact technologies and solutions for the next crop defence strategy against *B. cinerea* attacks.

Between hosts and necrotrophic agents, a series of molecular responses have been refined over the course of evolution, including the action of a range of hormones, the synthesis of molecules with specific antimicrobial action, and extensive reprogramming of gene expression presided over by countless transcription factors [95,169].

Necrotrophic pathogens, among which *B. cinerea* constitutes one of the most fearsome representatives, are less easily counteracted by the host through R genes, while the endogenous balance of several phytohormones such as salicylic acid (SA), jasmonic acid (JA), ethylene (ET), brassinosteroid (BR), and others assumes a particularly important role [170,171]. We then come to analyze some experimental data on the role of some phytohormones in the interaction between *B. cinerea* and host plants (model and cultivated plants).

It has been found that *Arabidopsis* mutants characterized by altered JA content/sensitivity are more susceptible to different *B. cinerea* isolates [172]. Indeed, these genotypes exhibited a highly altered molecular response to infection compared with controls. JA has also been found to activate defences against necrotrophic diseases especially in the presence of ET [173]. In contrast, SA-related signaling primarily leads to activation of the host’s defensive response against biotrophic pathogens. So, increasing the level of SA in the host can have negative effects on the plant’s defences; in fact, it has been shown, for example, that in *Arabidopsis* plants mutant for the *B. cinerea* susceptibility gene, *AtPLC2*, there is a low production of ROS and an intensification of SA signaling [174].

El Oirdi et al. [175] found that *B. cinerea* produces an exopolysaccharide that works as an elicitor of SA signaling, which in turn goes against the activation of JA signaling with a positive effect on necrotrophic growth on tomatoes. This is therefore an excellent strategy for the pathogen to subvert the host’s defences. Recently, in *Arabidopsis*, *BFP1* has been shown to be a key host gene to reduce the rate of JA catabolism and counteract the virulence of *B. cinerea* [176]. In rose, two transcription factors (RhEFR005 and RhCCCH12) that respond to JA and ET signaling were found to exert control over endogenous cytokinin levels, which in turn is a positive element in reducing host damage by *B. cinerea* [177].

Regarding auxins, it is interesting to report that in the tomato-*B. cinerea* interaction, a molecular signal [Phytosulfokine (PSK), a disulfated pentapeptide] from the host has been characterized, which when activated leads to increased cytosolic calcium and activates an auxin-dependent pathway that enhances immunity to *B. cinerea* in tomato [178]. On the other hand, it is known that *Arabidopsis* mutants defective in the auxin-stimulated SCF ubiquitination pathway showed susceptibility to *B. cinerea* attack [179]. Similarly, it was found that exogenous treatments with indole-3-acetic acid (IAA) and gibberellic acid (GA_3_) have a promoting effect on *B. cinerea* resistance, while the opposite occurs if ET and abscisic acid (ABA) are administered [180]. As regards cultivated tree plants, it is noteworthy that the administration of 50 mg mL^−1^ IAA in *Actinidia* significantly improves the response to *B. cinerea* [181].

ET represents a hormone that is known to be involved in the response to a variety of abiotic stress sources but also has a considerable role in biotic interactions. Regarding the tomato-*B. cinerea* interaction, very interesting is the work carried out by Díaz and collaborators [107]. In fact, in addition to carrying out exogenous treatments, the authors appropriately used in their experimentation a series of mutants for the synthesis and/or perception of hormones (e.g., *Never ripe*, *Epinastic*, and *Defenseless*) concluding that ET is important for the expression of tomato resistance to *B. cinerea*. In addition, the *Defenseless* mutant, deficient in JA synthesis, showed a significant increase in susceptibility to *B. cinerea*.

As for ET response factors (ERFs), numerous members are often present in plants, and some of them play a role in immunity. For example, ERF5 and ERF6 transgenic plants showed an increased resistance level against *B. cinerea* [182]. In the interaction between tomato and *B. cinerea*, Deng et al. [183] studied the factor SlERF.C1. These authors obtained plants deleted or overexpressing the gene encoding for this ERF and showed that in tomato fruits, *B. cinerea* aggression triggers *SlERF.C1* expression and ET production, resulting in *SlMPK8* transcription. The subsequent step is the phosphorylation of SlERF.C1 at a specific residue to activate the expression of PR genes. Various elements of the molecular chain of events that govern the response of tomato fruits to *B. cinerea* have therefore been deciphered.

In PAMP-triggered immune responses, it is very important for the host to recognize molecular signals of the pathogen as necrosis- and ET-inducing-like proteins (NLPs). In *Arabidopsis*, Ono and colleagues [184] showed that a central role is played by the RLP23 receptor of NLP peptides. In fact, RLP23 is required for *Arabidopsis* immunity against *B. cinerea*, and indeed, the defective mutant of this receptor exhibits high susceptibility to gray mold. Moreover, the authors showed that the RLP23 role is active at the pre-invasive steps of interaction.

It is important to remember that for plants, a constitutive and repeated immune response has negative effects on growth, and therefore a fine hormonal balance must be established to respond effectively to pathogens but without excessive damage to host growth. These aspects are even more prominent when it comes to plants of agronomic interest [185]. To date, the molecular mechanisms that preside over this adjustment are still poorly characterized [186].

In the activation of the plant immune response, as expected, transcription factors play a key role. With reference to the plant-*B. cinerea* interaction, various data suggest that the role of WRY33 is very important. In *Arabidopsis*, it has been established that WRY33 is phosphorylated by MAP kinases following infection by *B. cinerea*, and this molecular step presides over the activation of the *PAD3* gene, which leads to the biosynthesis of camalexin [187]. In addition, WRY33 is also crucial in the host hormonal response [188]. In fact, the *wrky33* mutant of *Arabidopsis* is extremely susceptible to *B. cinerea* and shows a very high ABA endogenous level. The authors demonstrated that WRKY33 directly affects the activity of two essential genes for ABA biosynthesis: *NCED3* and *NCED5*. The crucial role of WRKY33 has also been investigated in crop species, such as tomato, where the reduction of cell death and ROS accumulation in the infected area appeared as key events [189,190].

As previously reported, characterizing the molecular mechanisms of immunity may facilitate the development of new solutions to combat *B. cinerea* in nature. For example, the patented elicitor AsES is a member of the subtilisin-related alkaline proteases, and it is precisely its proteolytic activity that triggers a systemic defence, response against pathogens in strawberries. Hael-Conrad et al. [191] demonstrated that AsES generates a defence response in a dose- and time-dependent manner against *B. cinerea*. Interestingly, hormones in SA-, JA-, and ET-induced signaling pathways are important for activating AsES-dependent responses. Therefore, the authors suggest that elicitors can be used to induce plant defenses and could represent an alternative to the use of fungicides.

An attractive strategy could be chemical interference with the mechanisms of pathogens that can detoxify, for example, ROS. In this regard, an interesting target gene could be *BcTol1*. Yang et al. [192] showed that *BcTol1* is differentially regulated to enhance BcCcp1 secretion during the early phase of infection. Inactivation of *BcTol1* or *BcCcp1* leads to dramatically reduced virulence of *B. cinerea*. Moreover, the authors identified two BcTol1-targeting small molecules with a role in the prevention of *B. cinerea* invasion.

It may also be useful to develop treatments to promote activation of the immune response. This is what has been done in lettuce by exogenous cellobiose treatments. This product of cellulose hydrolysis may actually function as a type of pathogen/damage-associated molecular pattern to induce plant innate immunity. He and colleagues [193] determined that below 60 mg L^−1^, cellobiose administered on lettuce can have positive effects on plant response to *B. cinerea* by activating high activities of β-1,3-glucanase and antioxidant enzymes at the early stage of pathogen infection. Interestingly, EDS1, PTI6, and WRKY70, which are cellobiose-activated core regulatory factors such as SA signaling, played a crucial role in modulating plant responses.

A recent and interesting experiment evaluated whether a natural, volatile, and sustainable metabolite can be used to control *B. cinerea* on crop plants. Felemban and colleagues [194] determined that a carotenoid degradation product, β-ionone (apocarotenoid), suggests interesting uses. In *Arabidopsis*, the authors showed that the exogenous supply of this apocarotenoid causes profound transcriptional reprogramming and changes on the endogenous level of hormones such as ABA, JA, and SA. These responses had a positive effect on plants when they were inoculated with *B. cinerea*. This remarkable increase in the level of resistance toward *B. cinerea* was also confirmed in tobacco and tomato plants. Moreover, even transgenic plants characterized by an endogenous level of β-ionone showed less damage from *B. cinerea* when inoculated.

An interesting natural substance to control *B. cinerea* may also be chitosan. This is a highly biocompatible biopolymer due to its biodegradability, bioadhesivity, and bioactivity. For these reasons, it displays a wide range of applications. Among these, chitosan can induce plant defence mechanisms (defence priming). De Vega et al. [195] focused their attention on defence priming by chitosan. The use of chitosan results in induced resistance (IR) in solanaceous and brassicaceous plants. In tomato plants, increased resistance has been related to priming callose deposition and JA accumulation. Data from large-scale transcriptomic analysis showed that chitosan triggers gene expression at early stages after infection. Moreover, two new tomato genes with a typical priming profile were found: Avr9/Cf-9 rapidly elicited proteins 75 (*ACRE75*) and 180 (*ACRE180*). Transient and stable overexpression of *ACRE75*, *ACRE180*, and their *Nicotiana benthamiana* homologs showed that they are positive regulators of plant resistance against *B. cinerea*. These findings provided helpful information for searching for strategies to protect *Solanaceae* plants against *B. cinerea*.

Further examples of natural substances effective against *B. cinerea* can also be provided by some essential oils. In apple fruit, treatments with thyme oil induced resistance against *B. cinerea* through the priming of defence responses. Data suggested that the *PR-8* gene in the host has a crucial role in the efficiency of antimicrobial effects to contrast gray mold [196]. Other essential oils (EOs) have been tested against *B. cinerea*. In a recent study, a strong reduction in the growth of *B. cinerea* was obtained using a very low dose (0.5 mg mL^−1^) of *Cinnamomum cassia*, *Litsea cubeba* var. *formosana*, and *O. vulgare* EOs [197]. Specifically, the active metabolites, carvacrol and thymol, completely inhibited the germination of *B. cinerea* spores at a concentration of 300 μg mL^−1^, while the same substances induced collapse and damage to the treated *B. cinerea* mycelia as observed by scanning electron microscope (SEM).

Natural allies to counteract *B. cinerea* devastation in plants can also be simple amino acids. In this regard, both the in vitro and in vivo experiments against gray mold conducted by Li and colleagues [198] clarified that the amino acids of interest may be L-methionine and L-arginine. Analysis of the effects induced on the mycelium showed disruption of the cell membrane, lipid peroxidation, and abnormal development of hyphae. The authors argue that L-methionine or L-arginine are useful natural substances to control gray mold postharvest in both fruit and vegetables.

Among the most interesting *B. cinerea* control strategies to be implemented in nature on crops is the use of biocontrol microorganisms [199,200,201,202,203]. In-depth analysis of this complex topic is beyond the scope of this review. However, here, we hope that in the future, innovative research topics such as transcriptomic analysis on key biocontrol agents will also be encouraged in order to evaluate the role of specific genes in enhancing (or worsening) their ability to counteract the action of the pathogens. In this context, Zheng et al. [204] analyzed the interaction between *Pantoea jilinensis* and *B. cinerea* to understand the molecular details of biocontrol mechanisms. The effective strategy has been to silence specific genes to better understand their role.

What if a waste to be disposed of at a specific cost became a weapon against *B. cinerea*? This question was answered by Zhao and colleagues [205] by evaluating whether an agro-industrial waste product such as sunflower receptacles can be effective against *B. cinerea*. Indeed, the sunflower receptacle has proven to be a source of interesting diterpenes for use in counteracting *B. cinerea* infection.

An alternative strategy to combat *B. cinerea* may be outlined in the future by the study of long non-coding RNAs (lncRNAs) that this pathogen induces in the host. Chen and colleagues [206] recently addressed this hypothesis. The authors identified, at the level of tomato fruit, nearly three hundred different lncRNAs that react to *B. cinerea.* Specifically, a greater amount of antisense lncRNAs was found to target genes enriched in hydrolase activity. The roles of these lncRNAs were further investigated by VIGS (Virus-Induced Gene Silencing) experiments with a relative knockout approach. Therefore, the collected data could support references for the role of specific lncRNAs in inhibiting *B. cinerea* by modulating the expression of defense-related genes or influencing hydrolase activity. Furthermore, in the same host-pathogen interaction, other authors have recently focused on the study of specific miRNAs and siRNAs [207].

## 4. RNA Interference (RNAi): First Evidence and an Overview of Molecular Mechanisms

The control of *B. cinerea* is often quite difficult due to its wide host range and its environmental persistence. The most common strategy to reduce the spread of *B. cinerea* is through chemical means, generating not only the problem of the development of resistance but also of human health and the environment [26]. The flexibility and plasticity of the pathogen in challenging the defense mechanisms of plants, along with its ability to evolve strategies for escaping chemicals used for its control, pushed research toward the development of new control strategies for a safer and more environmentally friendly management of gray mold diseases. In this context, RNAi-based technologies are arising for the development of new control strategies, and they could benefit from information provided by new genomic sequencing (NGS), which offers greater possibilities to combat the pathogen. In RNAi-based pathogen control, a specific nucleotide sequence of dsRNA could be simply applied to plants as a potential alternative to conventional fungicides [10,11,12].

RNAi is an ancient mechanism strongly conserved from yeasts to humans during the evolution process [208,209,210]. Its formal discovery dates to 1998, when Andrew Fire and colleagues discovered the function of dsRNA in gene silencing events. Even then, it was evident that RNA could be used to interfere with the expression of a gene [211]. Already in 1985, John C. Sandford and Stehpen A. Johnston, illustrating the applications of the parasite-derived resistance, suggested the use of RNA complementary to that of the parasites to block the infection through anti-sense strand interference due to the formation of an RNA-RNA duplex [212]. Anyway, it was previously thought that the RNAi was mainly due to an anti-sense hybridization mechanism between the inserted RNA and the target mRNA transcript. Soon after, in 1995, in a study by Su Guo and Kenneth J. Kemphues on *Caenorhabditis elegans*, it was shown that sense RNA was as efficient as antisense RNA in decreasing gene expression [213]. They were studying the *PAR-1* gene, which encodes a putative Ser/Thr kinase with similarity to kinases from yeasts and mammals and is necessary for directing the polarity of *C. elegans* embryos. When they injected sense or antisense RNA into the gonads of worms, an average of 50% of worms arrested their development, showing a phenotype close to the *par-1* mutant.

The breakthrough of Andrew Fire, Craig G. Mello, and colleagues was to test the synergy of sense and antisense RNAs. The *unc-22* gene was chosen for initial comparisons of activity. *unc22* encodes an abundant but nonessential myofilament protein [214]. They found that the dsRNA mixture produced interference much better than sense or antisense RNAs alone [211]. Notably, the results of this interference were evident in both the injected animals and their progeny. They also noticed that a few molecules of dsRNA were sufficient to have a huge interference, guessing a non-stoichiometric process due to an amplification element in the whole silencing process.

Starting from this crucial discovery, some previously conducted studies on homology-dependent gene silencing mechanisms have been reevaluated as they may share a common biological basis. In 1990, C. Napoli and colleagues were working with transgenic petunias with the goal of altering flower pigmentation. They introduced a chimeric *CHALCONE SYNTHASE* (*CHS*) gene to allow its overexpression, which instead led to blocking of anthocyanin biosynthesis, and flowers showed total or partial white phenotypes. They found that the transgene was as inactive as the endogenous gene and was therefore somehow able to suppress the expression not only of the homologous endogenous gene but also of itself in a phenomenon called co-suppression [215].

Co-suppression phenomena were not exclusive to plants, but similar events were also observed in the fungus *Neurospora crassa.* Studies have been conducted to overexpress the *albino-1* (*al-1*) gene required in carotenoid biosynthesis, which gives the typical orange pigmentation to the fungal colony. However, around 30% of *N. crassa* transformants showed an albino phenotype identical to that of *al-1* mutants. The observed phenomenon was termed ‘quelling’, understood as a PTGS event [216]. Co-suppression in the broad sense, as defined by Jorgensen [217], has also been observed in *Drosophila* through white-*alcohol dehydrogenase* (*adh*) transgenes, and, furthermore, a gradual reduction of expression in the transgenes and the endogenous gene was found to be related to an increased dosage of the transgene [218].

In the meantime, many laboratories were working on viral RNA. It was known that in plants, if there is sequence similarity between the virus and a transgene or an endogenous gene, Virus-Induced Gene Silencing (VIGS) can occur, and plants react to RNA viruses by targeting them for destruction [219,220,221,222,223]. By analogy with RNA interference in animals, this mechanism is thought to involve the transformation of dsRNA into siRNAs. An RNase complex is then guided by the base pairing of the siRNAs to specifically target single-stranded (ss) RNA [224,225]. After the discovery by A. Fire, C. G. Mello, and colleagues, it was clear that both transgene arrays and replicating viral RNA generate dsRNA, and a new term was coined: RNAi [211].

The RNAi features imply the existence of a mechanism capable of not only triggering but also amplifying the silencing signal as an active response to foreign RNA. Subsequently, comparative studies were conducted of the *C. elegans* genes required for RNAi with those required in other organisms such as *Drosophila*, plants, and fungi [226,227,228,229]. From these analyses, it emerged that the silencing process, variously referred to as PTGS, co-suppression, quelling, and RNAi, indicated a common underlying mechanism that echoed an ancient origin in a common ancestor of plants, animals, and fungi [215,216,230,231]. In addition to these important findings, after PTGS plant studies, small RNAs (sRNAs) were shown to be produced [232] and consecutively identified as the common key molecule that specifically drives RNA silencing events [233,234,235,236].

RNAi-based pathways are used by eukaryotic organisms for several regulatory mechanisms, such as control of genes and genome integrity, defense against viruses, and control of developmental factors. RNAi seems to be involved in the control of transposable elements (TEs) in plants and animals [226,237,238,239]. Mutations in RNAi pathway genes can lead to a loss of silencing effect along with increased activity of TEs [226,237,239,240]. In plants, RNAi is closely linked to viral defense; in fact, mutants of the gene related to the PTGS pathway have shown increased susceptibility to viruses, and, on the other hand, many plant viruses have developed genes to hinder the RNAi response in wounded cells [209,241,242,243,244,245,246,247].

RNA-dependent transcriptional silencing can also regulate heterochromatin formation involving histone H3 methylation for controlling gene expression [248]. In fact, silencing of RNAi genes can lead to a loss of heterochromatin and centromeric functions necessary during mitosis for chromosome segregation [249,250,251,252]. Moreover, its regulation of heterochromatin can interfere with meiotic genes regulated in turn by long terminal repeat (LTR) retrotransposons. When these LTRs are transcribed, silencing of the affected locus can occur [208,253]. Defects in the RNAi pathway or the loss of these LTRs can affect the expression of meiotic genes [254]. Regulation of meiosis can also take place through RNAi, for example, in *N. crassa* during meiosis from unpaired regions of chromosomes [255].

Furthermore, genes of the RNAi pathway appear closely linked to the regulation of developmental stages in different organisms. The *Arabidopsis* ARGONAUTE1 (AGO1) protein was first identified during a study of developmental mutants and was only later shown to play a role in RNAi [228,256]. Studies on *C. elegans* mutants for the *DCR-1* gene showed heterochronic phenotypes close to the *lin-4* and *let-7* mutations. *lin-4* and *let-7* are small temporal RNA (stRNA) genes that regulate stage-specific development [257]. In *Drosophila*, the *bantam* gene encodes a 21-nt microRNA, which controls development and promotes tissue growth by regulating the pro-apoptotic gene *hid* that recognizes complementary sequences in its 3′-UTR region [258]. In all the above-mentioned cases, what is clear is that RNAi is an important pathway between organisms that can be used for several crucial purposes by many different species [259,260].

**Figure 5:**
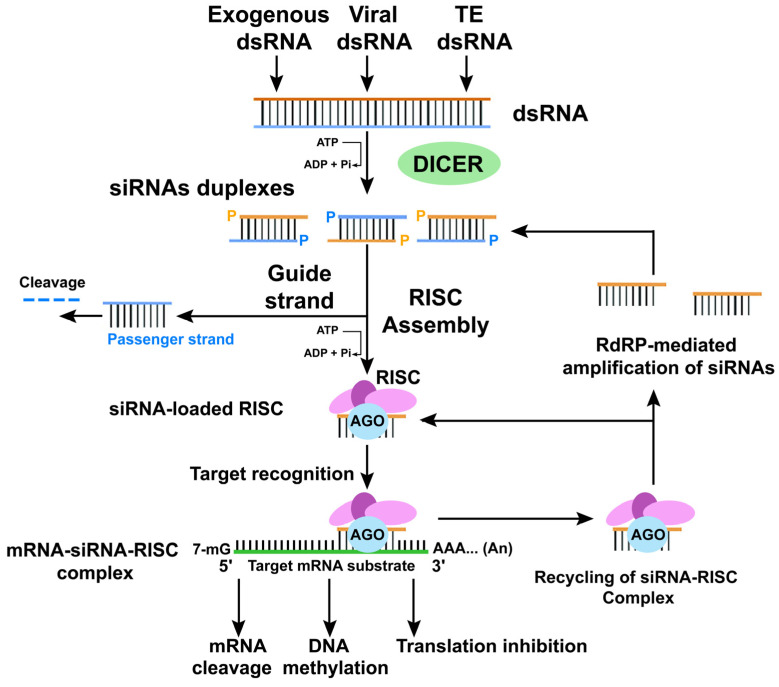
General scheme of a step model of the RNAi mechanism. TE: transposable element; siRNA: small interfering RNA; AGO: Argonaute; RISC: RNA-Induced Silencing Complex; RdRP: RNA-dependent RNA polymerase. Modified by Plasterk [270] and Limera et al. [271].

RNAi or RNA silencing are general terms for a peculiar collection of events in which short RNA molecules trigger the repression of homologous sequences (Figure 5).

Although they may vary in some details, RNAi in animals, PTGS in plants, and quelling in fungi follow all the same highly conserved pathway, referring to an ancient common origin [261,262,263,264]. The characterization of the phenomena underlying RNAi, following numerous studies, has made it possible to reveal the components and molecular pathways that regulate this mechanism [265]. The basic process involves dsRNA being cleaved into smaller sRNA duplexes, molecules of 21–25 nucleotides, first described in plants [232]. They are very specific for target sequences as they can guide recognition and address the cleavage of homologous mRNA.

The dsRNA molecule that triggers the pathway can originate in the nucleus or in the cytoplasm in several ways, such as viral replication, simultaneous synthesis of sense and antisense RNA strands, transcription through inverted DNA repeats, and by RNA-dependent RNA polymerases (RdRPs) [266,267]. The sRNAs can originate from endogenous or exogenous dsRNAs, and two important types of sRNAs can commonly be found: microRNAs (miRNAs) and small interfering RNAs (siRNAs) [265].

Subsequently, sRNA production is a stepwise process, including the activity of class II and class III RNase-III-type endonucleases, called DROSHA and DICER, respectively, and containing RNase III and dsRNA binding domains (dsR-BD) [234,268,269].

DROSHA specifically processes miRNA precursors but not long dsRNAs. The miRNAs are first transcribed as long and hair-pinned primary transcripts (pri-miRNAs) and then processed by DROSHA, which uses dsRNA-binding domains (dsRBDs) for pri-miRNA recognition [265]. This results in the formation of a miRNA precursor of approximately 70 nt (pre-miRNA) in the nucleus [272,273,274]. When DROSHA removes the folded miRNA precursor, a 5′-phosphate and a 2-nucleotide 3′ overhang stand at the base of the miRNA structure [273,275]. Later, the exportin-5 (XPO5)/RanGTP complex, a nuclear export receptor, delivers the pre-miRNA to the cytoplasm [276,277,278]. At this point, an enzyme of the DICER family processes pre-miRNAs and long dsRNA [274]. The miRNAs, after being processed by DICER, associate with the AGO in the RNA-Induced Silencing Complex (RISC), and one of the strands is eliminated. The remaining strand is linked to the AGO protein, which can recognize sequences in the 3′ UTR of target mRNAs. According to the homology with the target sequence, miRNAs can mainly regulate translation rather than degradation of mRNA [279]. In plants, because miRNAs are highly complementary to the target mRNA, cleavage is the primary mode of action. Only in recent years have translation inhibition pathways been discovered in plants [280].

DICER enzymes are evolutionarily conserved as they were identified in many organisms, including *C. elegans*, plants, *N. crassa*, *Drosophila*, and humans [269,281,282,283,284,285,286,287,288]. DICER triggers dsRNA cleavage, producing a 21–25-nt dsRNA ready for RISC loading [236]. The enzyme contains two RNase III domains, which can sometimes form a pseudo-dimer, and each cleaves one strand of the duplex [289,290]. A PIWI/AGO/ZWILLE (PAZ) domain allows RNA recognition, thus playing an important role in the biology of RNA silencing mechanisms [291]. The PAZ domain binds to the 3′ end [292], preferring 2-nt overhangs of dsRNA [293], and to the C-terminal dsRBD. A dual-pocket architecture has been suggested for the PAZ domain, enabling the stabilization of the 2-nt 3′ overhang along with the reorientation of the processed dsRNA to assist its loading on RISC [294]. The physical distance in the overall architecture of this enzyme between the PAZ and RNase III domains could lead to the production of different lengths of siRNA [295]. The complex also has an N-terminal RNA helicase for substrate unpacking [269]. It has also been suggested that the helicase domain may function to guide dsRNA substrates to PAZ-RNase III domains [296].

At this point, the siRNAs are incorporated into the RNA uploaded on RISC, the multiprotein complex assembled in the cytoplasm [233,297,298]. The first step is the unwinding of siRNA duplexes, in which only the antisense strand is charged into the RISC and targets the cognate mRNA for degradation [299,300]. This process is guided by highly precise base pairing of the antisense or guide strand and the target mRNA; in particular, the recognition site is represented by the so-called seed sequence (nt 2–8). It could be that the guide strand recognizes its targets within the seed-sequence nucleotides 2–5, and after undergoing conformational changes, confirms the target with nucleotides 6–7 and then with the remaining ones [301]. Thus, the mRNA is cleaved at the middle of the siRNA strand and subsequently degraded [299,302]. The main component of RISC is AGO proteins, which are characterized by four main domains: amino-terminal (N-terminal), PAZ, MIDDLE (MID), and PIWI in all organisms, from bacteria to humans [303,304].

A variable domain (ND) is present in the N-terminal region, which helps to separate the siRNA-target duplex after slicing [305]. The 5′-phosphate of the antisense strand is put in the right position by the MID domain, which interacts with the nucleotide specificity loop with the help of the PIWI domain [306]. The 3′ end of the RNA binds to the PAZ domain [292,307], which provides a binding pocket for the two-nucleotide overhanging [308]. The PIWI domain is such as ribonucleases H (RNaseH) and is the core of enzymatic cleavage activity in the RNA-mediated silencing complex, which requires Mg^2+^ for its activity [303,309]. The MID-PIWI section does not encounter considerable conformational changes to contain the target, while the PAZ domain seems to involve rearrangement and releases the guide related to target binding [310]. At the end of the process, the target mRNA is cut and degraded.

In addition to the main mechanism of RNAi, in the so-called transitive RNAi, an RdRP can use previously produced siRNAs as primers with the aim of amplifying the abundance of dsRNA, and DICER will subsequently cleave the new dsRNA into siRNA with a diffusion silencing signal [311]. RdRPs have been identified, for example, in *C. elegans*, plants, and filamentous fungi [311,312,313,314]. Another spreading signal pathway is systemic RNAi, in which an amplification mechanism allows the production of the RNAi signal, which is subsequently moved between cells [315].

In plants and fungi, sRNAs also mediate transcriptional gene silencing (TGS) through mechanisms involving DNA methylation, as observed in *Arabidopsis* [316,317], or histone methylation, as demonstrated in budding yeast for pericentric heterochromatin formation [318].

### 4.1. RNAi in the Fungal Kingdom

The RNAi mechanism has been fully investigated in fungi, and its role differs throughout the fungal kingdom, since it has evolved according to the peculiarities and requirements of each species. Fungi have exploited RNAi to regulate several functions, such as genome integrity, defense against exogenous DNA, virulence, and development, using it as a versatile evolutionary tool capable of enhancing the great fungal diversity. Fungi served as model organisms during the discovery of RNAi, particularly *N. crassa*, for which one of the first silencing events was reported. *N. crassa* has different RNAi mechanisms; the first described is quelling, which suppresses virus infections and TEs [216,312]. Quelling is the canonical RNAi pathway triggered by a transgene homologous to an endogenous gene. The quelling pathway consists of an RdRP (*QDE-1*), two DICER-like proteins (*DCL1* or *DCL2*), an AGO (*QDE-2*), and the RecQ helicase *QDE-3* [281,312,319,320].

Another important mechanism described for *N. crassa* is the meiotic silencing of unpaired DNA (MSUD). MSUD has a remarkable ability to scan homologous chromosomes for unpaired DNA during meiosis. After the unpaired DNA has been identified, MSUD silences all RNA from the unpaired DNA along with any RNA transcribed from homologous sequences elsewhere in the genome, regardless of their pairing status [321,322]. This mechanism was also found in *Gibberella zeae* operating during prophase I [323]. MSUD involves some genes of the canonical RNAi pathway (*Dcl1*) along with *SAD-1*, a specific RdRP, the AGO *SMS-2*, and the *SAD-3* helicase, and all form a multiprotein complex that generates MSUD-associated siRNAs (masiRNAs) in the perinuclear region [324].

The first described function of RNAi is that of defense against viral infections through the production of virus-derived small interfering RNAs (vsRNAs), and it has been fully studied in *Cryphonectria parasitica* since it is considered a model in virus-fungus interactions [325]. *C. parasitica* mutants for RNAi genes were strongly susceptible to mycovirus as they were impaired in the production of vsRNAs [325]. As a counter-defense, viruses can use RNAi suppressors; in fact, the mycovirus *Cryphonectria* hypovirus 1 (*CHV1*) expresses a Papain-like protease named p29, which blocks the expression of *dcl2* and *ago2* in the fungus, abolishing its immunity [326].

Similarly, in several *Aspergillus* species, RNAi appears to be related to virus-fungus interactions [327]. In *A. nidulans*, 341 virus-derived siRNAs indicate that RNAi acts as a defense mechanism against viruses [328]. On the other hand, viruses can also encode RNAi suppressors to block the RNAi viral defense response in *A. nidulans* [328]. The evolution in virus-host interaction has led to a fine-tuning of the RNAi mechanism so that both can benefit.

The RNAi mechanism can also control the spread of TEs, and its role in TEs repression has been illustrated in plants as well as in animals [226,329,330]. The role of RNAi in the control of TEs has also been studied in fungi. In *N. crassa*, a PTGS mechanism controls the LINE1-like retrotransposons without involving DNA methylation [331]. In *Schizosaccharomyces pombe*, siRNAs targeting the Tf2 retrotransposon were found, and the siRNAs appeared to be related to the methylation of histone H3 in lysine 9 (H3K9me) [332]. In *Magnaporthe oryzae*, approximately 10% of all observed sRNAs are mapped to LTR-retrotransposons [333]. In *Mucor circinelloides*, sRNAs produced by the canonical RNAi pathway regulate the TE located around the centromeric area (Grem-LINE1) [334]. Furthermore, another pathway called the non-canonical RNAi pathway (NCRIP) seems to play a role in silencing Grem-LINE1 by modulating the canonical pathway [335]. Similarly, *Puccinia graminis* f. sp. *tritici* produces a high amount of sRNAs in centromeric regions during infection, which induces silencing of TEs around centromeres [336].

The pathogenic fungus *Cryptococcus neoformans* has also been shown to use RNAi for the TEs control and maintenance of genome integrity, particularly during sexual reproduction, where an RNAi mechanism called sex-induced silencing (SIS) is used [337]. In *C. neoformans*, five genes (*Rde1–5*) are required for the suppression of HAR1 (DNA TE), and *Rde4*, involved in siRNA biogenesis, may have a role in TE control [338]. Moreover, along with siRNAs, miRNAs targeting TEs were detected in *C. neoformans*, suggesting their possible involvement in TE control [339].

Another important role of RNAi pathways is the regulation of endogenous genes related to metabolism or vegetative growth, as well as genes involved in sexual reproduction and pathogenesis [340,341].

In *M*. *circinelloides*, the RNAi mechanism goes beyond the canonical, epimutational, and non-canonical pathways. They all create a regulatory network for the control of endogenous genes [340,342]. Its basic RNAi mechanism works against transgenes, viruses, and transposons, but it also uses exonic siRNAs (ex-siRNAs) to modulate the expression of endogenous genes. In *M. circinelloides*, approximately 700 genes are regulated by ex-siRNAs, all of which play an important role in its development and physiology [343,344]. Mutants for ex-siRNA synthesis components show reduced growth, lower asexual sporulation, and altered hyphal morphology [345,346].

Similarly, *Trichoderma atroviride* produces sRNAs such as ex-siRNAs of *M. circinelloides*, related to development and growth [347], and *Fusarium graminearum* produces ex-siRNAs during ascosporogenesis [348].

Another RNAi mechanism shown in *M. circinelloides* is the non-canonical RNAi pathway (NCRIP), where the non-canonical features are the independence of DICER and AGO proteins. Here, another class of sRNAs called RdRP-dependent dicer-independent sRNAs (rdRNAs) is produced after mRNA degradation [349]. This pathway binds dsRNA but only cuts ssRNA. NCRIP shows a heterogeneity of functional roles. It reduces the epimutational pathway, and the loss of NCRIP increases the formation of drug-resistant strains. The NCRIP mechanism is involved in oxidative stress and sexual interaction and can also regulate a huge amount of genes mainly related to saprophytic growth (cellular metabolism, germination, and development) [335,349]. NCRIP also influences the control of retrotransposon expression, playing a key role in genome stability [350].

Moreover, in *M. circinelloides*, the epimutational RNAi pathway allows transient silencing of specific mRNA during a stress challenge [351,352]. This pathway is activated during exposure to antifungal compounds to enhance drug resistance through siRNA production [351]. The mechanism requires both canonical RNAi and NCRIP in a balance that triggers the activation of epimutation under stress factors and confers plasticity to the fungus under different conditions [351,353].

Epimutants were shown to grow during NCRIP inactivation as there was competition between the two RNAi pathways [349]. The three RNAi pathways are closely related, allowing *M. circinelloides* fine regulation in the control of mRNA levels to address environmental challenges for the fungus [335].

*N. crassa* produces, through at least four different mechanisms, microRNA-like RNAs (milRNAs) for the control of endogenous genes, which are produced starting from precursors with specific stem-loop RNA. milRNAs appear to silence endogenous targets with mismatches, as in animal miRNAs [354]. They can be involved in both the asexual and sexual phases, as has also been found for *F. graminearum* [355,356].

As shown above, a great diversity of RNAi-based mechanisms can control fungal gene expression, revealing its implications for several important functions connected with growth, morphology, and sexual development.

Along with the mentioned roles, RNAi can also affect heterochromatin formation, and *S. pombe* is considered a model for understanding the mechanism [357]. In *S. pombe*, heterochromatin formation depends on siRNAs from centromeric regions displaying numerous repeats [253,358]. These siRNAs link to the RNA-induced transcriptional silencing complex (RITS) and bind to the RNA transcripts of centromeric repeats [252,359]. RITS activity involves the histone methyltransferase Clr4, which catalyzes the lysine methylation in the histone H3 (H3K9me), inducing heterochromatin assembly [252,358,360]. Moreover, H3K9me, acting as a binding site, promotes chromatin-modifying proteins as well as RNAi components, thereby stimulating the amplification of siRNA and H3K9me domains in nearby centromeric regions [253,361]. RNAi-mediated heterochromatin formation and the H3K9me also outline a possible epigenetic inheritance pathway that includes the spreading of secondary siRNAs and H3K9me3 to the target gene and around it, where RNAi and H3K9me act synergistically to maintain silencing, achieving transgenerational epigenetic inheritance [362].

### 4.2. RNAi in Host-Pathogen Interaction

The growing interest in RNAi as an alternative strategy to control plant pathogens has led to a deep understanding of its involvement in host-pathogen interaction [363,364,365,366].

In *Colletotrichum gleosporioides*, DCLs have been shown to regulate several proteins directly related to or involved in fungal pathogenicity. Double-deletion mutants for *DCL1* and *DCL2* showed strongly reduced growth and conidiation, suggesting a role of the RNAi pathway in vegetative growth along with conidia formation. Furthermore, the double mutants were unable to cause lesions on *Hevea brasiliensis* leaves, showing a complete loss of pathogenicity. In fact, they presented a delay in germination and predominantly superficial hyphal growth on the host surface [367].

In *Magnaporthe oryzae*, sRNAs and the mRNA transcriptome of RNAi deletion mutants were analyzed to understand the role of the RNAi mechanism. The sRNA pathway seems involved in the preservation of the *M. oryzae* genome through transcriptional control of telomeric intergenic and repeated regions, supporting the role of RNAi in developmental processes, fungal growth, and virulence [368].

In *Sclerotinia sclerotiorum*, double-deletion mutants for *DCL1* and *DCL2* and mutants in the *agl-2* gene encoding the AGO protein showed markedly reduced growth and virulence before viral infection and even more severe devitalization after virus infections. This implied phenotypic changes such as reduced growth, decreased pigmentation, and slowed sclerotia formation [369,370].

In *Valsa mali*, AGO proteins have important roles in fungal pathogenicity and in environmental responses, in particular *VMAGO2* for H_2_O_2_ tolerance [371]. Similarly, *FoQde-2* deletion mutants for the AGO protein of *Fusarium oxysporum* f. sp. *lycopersici* showed reduced virulence on tomatoes; on the contrary, no effect on vegetative growth was demonstrated [372].

RNAi pathways in *F. graminearum* are involved in conidiation, ascosporogenesis, and pathogenicity. FgDcr1 and FgAgo2, which generate ex-siRNAs, can regulate the virulence of *F. graminearum* and could regulate the biogenesis of milRNAs by influencing gene expression, in particular perithecia-specific miRNA-like RNAs [355,373]. In addition, *F. graminearum* produces several mycotoxins to colonize host tissues. Analysis of the mutants showed that the amount of deoxynivalenol (DON) produced was strongly lower in wheat spikes when RNAi mutants infected them, suggesting that the RNAi may enhance mycotoxin production [373].

In the case of the citrus fruit pathogen *Penicillium italicum*, mutants for *Pit-DCL1* and *Pit-DCL2* were strongly less pathogenic, and critically stopped the biogenesis of milRNAs, which may be involved in the mechanism of cross-kingdom RNAi [374].

Cross-kingdom RNAi is an important feature of fungal pathogenesis as it consists of the trafficking of sRNAs between the host and the pathogen that promotes silencing in *trans* [375,376,377,378]. This mechanism was first illustrated in *B. cinerea*, which produced sRNAs to silence *Arabidopsis* and tomato immunity. It deviated from the host RNAi mechanism by inactivating host AGO1 proteins and suppressing immunity genes. The *Arabidopsis ago1* mutant showed resistance to *B. cinerea*, while the *B. cinerea dcl1-dcl2* double knockout mutant failed to produce these sRNAs, particularly Bc-siR3.1, Bc-siR3.2, and Bc-siR5, and showed reduced pathogenicity. This demonstrated that *B. cinerea* uses sRNAs as effectors to suppress plant immunity and successfully infect the host through a cross-kingdom sRNA trafficking mechanism [378].

Similarly, *Verticillium dahliae* depends on plant AGO1 for its pathogenicity [379]. The fungus produces sRNAs that are primarily directed at *Arabidopsis* AGO1-associated genes during infection, suggesting the use of sRNAs to silence host targets. Indeed, the *Arabidopsis ago1* mutant showed enhanced resistance to the pathogen [12].

The soybean pathogen *Phytophthora sojae* produces RNA silencing suppressors (PSR1 and PSR2) to repress host RNA silencing through the inhibition of sRNA biogenesis. In particular, PSR1 inhibits the synthesis of siRNAs and miRNAs, while PSR2 hinders the formation of siRNAs [380]. Furthermore, Hou et al. [381] found that PSR2 specifically reduces the accumulation of secondary siRNAs triggered by microRNAs miR161 and miR173. miR173 targets long non-coding RNAs of the *TAS1/TAS2* family, leading to *TAS1/TAS2*-derived secondary siRNAs. Both *TAS1/TAS2* secondary siRNAs and miR161 target protein-coding mRNAs from genes encoding the pentatricopeptide repeat (*PPR*), generating *PPR-*derived secondary siRNAs. *TAS*-and *PPR*-derived siRNAs are secondary siRNAs, and their biosynthesis depends on the activity of RDR6. RDR6 is involved in the production of bacterial-induced lsiRNA-1 and natural antisense transcript (NAT)-associated siRNAs (nat-siRNA, e.g., nat-siRNAATGB2). Knockout *rdr6* mutants are very susceptible to *Pseudomonas syringae* pv. *tomato*, *Agrobacterium tumefaciens*, and *Xanthomonas oryzae* pv. *oryzae* [382,383,384,385]. In addition*, rdr6* mutants of *Arabidopsis* showed hypersusceptibility to *B. cinerea*, *Phytophthora capsici*, and *V. dahliae* [379,381,386]. The contribution of RDR6 to plant immunity may be due to its key role in the production of secondary siRNAs that silence pathogen mRNAs [387].

*Puccinia striiformis* f. sp. *tritici* also uses this mechanism. In fact, it produces a microRNA-like RNA 1 (Pst-milR1) to suppress host defenses. Silencing of Pst-milR1 led to increased resistance of wheat to the pathogen [388]. Moreover, in *Blumeria graminis* f. sp. *hordei* and *B. graminis* f. sp. *tritici*, sRNA-seq analysis revealed the presence of specific sRNAs that target plant genes, altering important host functions in primary metabolism. This suggests a cross-kingdom RNA transference between these fungi and their hosts [389].

These examples support the role of cross-kingdom RNAi in pathogenesis in diverse species of fungi, bacteria, and Oomycota, suggesting its importance in the evolution of host-pathogen interaction.

## 5. RNAi-Based Technologies to Control Plant Pathogens: HIGS and VIGS

In host-pathogen interactions, hosts can also transport sRNAs into related pathogens to contain their virulence, as was recently discovered [12,390,391,392,393,394].

In *V. dahliae* infection, cotton plants respond by increasing the synthesis of miR166 and miR159 and delivering them to the fungal hyphae for specific silencing. In fact, they target genes that are fundamental to fungal virulence and which encode respectively *Clp-1* and *HiC-15*, a Ca^2+^-dependent cysteine protease, and an isotrichodermin C-15 hydroxylase contributing to fungal pathogenicity [394].

In the case of the root-knot nematode *Meloidogyne incognita*, when exposed to dsRNA targeting the signaling peptide 16D10 that promotes its root growth, a significant reduction in 16D10 mRNA was found. Moreover, in *M. incognita* infection of *Arabidopsis* producing 16D10 dsRNA, gall formation was reduced, indicating that the dsRNA produced in the plants was ingested by the nematode and then underwent the expected RNAi effect [395]. The plant engineering to produce dsRNA, called host-induced gene silencing (HIGS), demonstrates the delivery of dsRNAs from the host to interacting pathogens, supporting the evidence for cross-kingdom RNAi [390,396].

HIGS is a possible strategy for crop protection, as transgenic crops could produce customized dsRNA against the target pathogen, which will be processed into sRNAs against essential or pathogenicity genes [397].

The first report of HIGS against a fungal pathogen was conducted against *Blumeria graminis*. It is an obligate biotrophic phytopathogen tightly connected through the haustoria with the host cell for the supply of nutrients to the fungus. A biolistic bombardment of RNAi constructs was conducted in host plant cells and found that 16 out of 76 *B. graminis* target mRNAs led to a great reduction in haustoria production. These findings were confirmed by a VIGS approach using *Barley Stripe Mosaic Virus* (*BSMV*) as a proof of concept for the further development of transgenic plants engineered against *B. graminis* mRNAs related to genes involved in haustoria formation [396].

HIGS experiments have also been conducted on *Fusarium graminearum*. Transgenic plants expressing double-stranded RNA were produced, targeting all three *fungal sterol 14α-demethylase CYP51* genes, usually targets of DMI (demethylation inhibitor) fungicides such as azoles [398,399]. The resulting *Arabidopsis* and barley RNAi plants exhibited complete immunity to *F. graminearum* [399]. Similarly, RNAi constructs targeting different regions of *chitin synthase 3b* (*Chs3b*), a gene highly expressed during *F. graminearum* colonization, were used in wheat. This resulted in a notable reduction in fungal infection on both seedlings and ears [400].

In a HIGS study on *Puccinia triticina*, transformed wheat plants were used for the expression of hairpin RNAi constructs. They targeted a cyclophilin (*PtCYC1*) or a MAP-kinase (*PtMAPK1*) of the fungus, which led to a consistent reduction in their transcript abundance, resulting in reduced fungal growth in remarkably resistant plants [401]. Furthermore, transgenic wheat plants expressing a HIGS construct against *Puccinia striiformis* f. sp. *tritici* and targeting a *PsCPK1* subunit of the *protein kinase A* (*PKA*) gene showed a reduction in long infection hyphae and disease phenotype, resulting in increased resistance to the pathogen [402].

Plants previously infected with *Barley Stripe Mosaic Virus* (*BSMV*) strains with antisense sequences against *F. culmorum*, showed reduced levels of target transcripts in the pathogen and lower disease symptoms in wheat. Engineered wheat plants exhibiting an RNAi hairpin construct targeting the gene *FcGls1*, encoding a β-1, 3-glucan synthase, showed higher resistance to Fusarium Head Blight (FHB) in leaves and spikes as fungal hyphae presented severe cell wall deformities [403].

In *B. cinerea*, transgenic *Arabidopsis* plants expressing hairpin RNAs against DCL1 and DCL2 of the pathogen were analyzed. They showed smaller lesions and reduced fungal growth, demonstrating that RNAi signals moved from plant to fungal cells very efficiently, silencing target genes [12]. Furthermore, a VIGS approach was used [12,404]. The same *BcDCL1* and *BcDCL2* RNAi fragments were introduced into *Tobacco Rattle Virus* (*TRV*) to allow their expression in tomatoes. When tomato leaves were infected with *B. cinerea*, they showed reduced or no symptoms of the disease [12].

The above-mentioned studies are just a few examples of HIGS used against plant pathogens, and there are many other successful works conducted on insects, nematodes, Oomycota, fungi, and even parasitic plants [405,406].

As previously reported, in addition to the use of transgenic plants, recombinant viruses have been used to induce RNAi. A virus can be engineered to include mRNA sequences and infect a plant that will use its antiviral RNAi response against the invading virus RNA and the included sequence [221]. This RNAi-based technology called VIGS has been widely used to study the function of plant genes, including those related to plant defense against pathogens [221,397,407,408].

HIGS strategies against *Uromyces appendiculatus*, the causal agent of common bean rust, have proven difficult and inefficient because beans are recalcitrant to genetic transformation [409,410]. Therefore, *Bean Pod Mottle Virus* (*BPMV*) was used to express sRNAs corresponding to effector mRNAs of the fungus, and a reduction in rust disease symptoms was achieved [411].

In the necrotrophic pathogen *Sclerotinia sclerotiorum*, VIGS was used to target soybean respiratory burst oxidase homologues (RBOHs) by a *Bean Pod Mottle Virus* (*BPMV*), obtaining high resistance to *S. sclerotiorum* with greatly reduced ROS levels [412].

Genome-wide association (GWAS) studies for virulence analysis of *B. cinerea* on *Solanum lycopersicum* and *S. pimpinellifolium* indicated that the genetic architecture of virulence is highly quantitative [413]. This confirms the widely accepted idea that the virulence of *B. cinerea* is polygenic and depends on a complex and multiple set of pathogenicity factors that give the pathogen the ability to infect multiple host plants. Therefore, genetic improvement of crops against *B. cinerea* is complex because many different loci are often affected [414,415,416]. Deepening knowledge on this topic requires different levels of study, such as mapping of different quantitative trait loci (QTLs), use of multiple molecular markers, and molecular and physiological analysis of different aspects of plant-host interaction. For example, updated profiles exploring the broad genetic diversity of *B. cinerea* have provided indispensable insights into the genetic and molecular mechanisms employed by the fungus to attack its hosts [417]. In addition, the discovery of candidate genes for resistance to *B. cinerea* will provide, in the future, an important theoretical reference for the mechanisms of resistance to gray mold and the research and selection of gray mold-resistant plants [416]. The isolation of countless candidate genes for resistance and susceptibility against *B. cinerea* requires extensive validation work to reliably assess the role of individual genes. Without a doubt, it is at this level that RNAi methods could facilitate genetic improvement work, especially if the techniques are efficient and simple at the same time [418]. Clearly, validation work is also fundamental in order to then undertake the most appropriate genomic editing programs. RNAi methods, especially VIGS, are useful to understand how the level of damage caused by the pathogen can vary due to the silencing of a specific gene. Furthermore, VIGS is very important to assess whether this also has undesirable effects on the plant, such as variation in vigor and productivity [419]. Table 1 shows some recent studies on the role of putatively important genes in crop breeding that also used RNAi techniques.

**Table 1:** Examples of recent research in which the role of resistance and susceptibility genes has been validated through RNAi methods in different crops.

Host	Resistance Gene	Susceptibility Gene	Function of Protein	RNAi Method	Reference
*Rosa chinensis*		*RcbHLH112*	Basic/helix-loop-helix transcription factor	VIGS	[420]
*Rosa chinensis*	*RcbZIP17*		Transcription factor with multi-functionality roles	VIGS	[421]
*Rosa chinensis*		*RcTBL16*	Protein involved in the O-acetylation pathway of plant cell walls	VIGS	[422]
*Gerbera hybrida*		*ghPG1* and *ghsit*	Polygalacturonase enzyme and ABA-aldehyde oxidase enzyme	VIGS	[415]
*Solanum lycopersicum*		*SlRCAR9*, *SlRCAR11*, *SlRCAR12*, and *SlRCAR13*	Abscisic acid receptors	Genetic transformation	[423]
*Solanum lycopersicum*	*DEK*		Protein with DNA-binding domains with multi-functionality roles	VIGS	[424]
*Solanum lycopersicum*	*SlSKIP1b*		Protein acting as a component of the spliceosome and have roles in several signaling pathways	VIGS	[425]
*Morus atropurpurea*	*mno-miR164a*		microRNA with a silencing function on *MnNAC100* (a transcription factor gene)	Genetic transformation and VIGS	[426]
*Actinidia chinensis*	*Ac-miR160d*		microRNA with a role in increased antioxidant enzyme activities and phytohormone levels	VIGS	[427]
*Fragaria* × *ananassa*		*FaWRKY29* and *FaWRKY64*	WKRY transcription factors with a role in growth, development, and stress response	VIGS	[428]
*Fragaria* × *ananassa*	*FaWRKY11*		WKRY transcription factor with a role in growth, development, and stress response	VIGS	[429]
*Solanum tuberosum, S. lycopersicum*		*DND1*	A protein associated with a susceptibility trait	Genetic transformation	[430]
*Gossypium hirsutum*		*HDTF1*	Homeodomain transcription factor	VIGS	[431]

However, the scenario of genes given in Table 1 is not exhaustive of the information gained on the susceptibility and resistance traits of plants against *B. cinerea*, and, therefore, we refer to other, more specific reviews for this wide topic [432]. The complex genetics of resistance make it problematic to decode critical genetic regulators of resistance in crop plants. Key necrotrophic virulence effectors are gradually emerging, and it is important to note that the identification of interactions between effectors and their virulence targets represents a very relevant topic for future research [432]. The use of RNAi methods, for example, to validate putative virulence factors of pathogens and plant susceptibility genes may be of considerable interest.

Plant protection achieved by VIGS or HIGS leads to the development of Genetic Modified Organisms (GMOs). Indeed, these cross-kingdom RNA technologies require transgenic plants or plants infected with engineered viruses [397]. Moreover, the feasibility depends on the possibility of transforming the plants and the genetic stability of the resulting crop species. Even when the transformation is successfully integrated into the genome, it takes time to develop and release a commercial cultivar, considering the regulatory process and public opinion about GMOs [433].

## 6. Environmental RNAi and SIGS Development for Plant Pathogen Control

Environmental RNAi is the uptake of external dsRNAs by organisms, that triggers RNAi [434]. It is an attractive alternative for plant disease control because it does not require modifications in crop genomes and results in high compliance for multi-target strategies. Environmental RNAi does not take as long as HIGS or VIGS and can be directed to any pathogen or pest that proves sensitive to RNAi approaches [433,435]. Moreover, it is difficult for pathogens to generate mutations aiming to escape RNAi, and the mechanism remains active even with various mutations in dsRNA [436]. Topical applications of dsRNA do not release toxic substances into the environment, nor do they cause alterations in crop genes. Given these premises, a growing interest in the application of environmental RNAi is rising.

Uptake of environmental dsRNA was first demonstrated in nematodes by injecting dsRNA into adult animals or by feeding them with bacteria engineered to produce specific dsRNA segments [211,437].

Environmental RNAi has also been demonstrated in insects through ingestion of dsRNAs provided by an artificial diet, triggering RNA interference in *Diabrotica virgifera virgifera* Le Conte, the western corn rootworm (WCR) [438]. Similarly, external uptake of dsRNA was shown in *Myzus persicae* by feeding dsRNA produced by *Arabidopsis* expressing RNA hairpin against the *Eph* genes, by recombinant *Tobacco Rattle Virus* (*TRV*) infecting *Nicotiana benthamiana*, or also with dsRNA synthesized in vitro [439].

**Figure 6:**
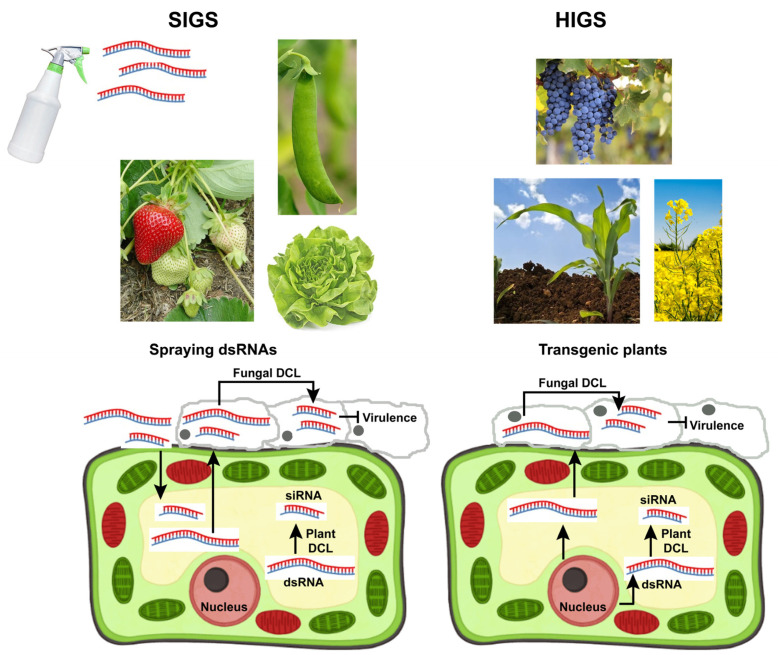
Schematic representation of SIGS and HIGS for the control of pathogenic fungi. DCL: DICER-Like Protein. Modified by Sang and Kim [442].

The mechanism of environmental RNAi was later discovered in fungi (Figure 6). Fungi can uptake sRNA duplexes and long dsRNAs ranging from 21 nt up to 800 nt [12,440].

When sRNAs or long dsRNAs were fluorescein-labeled and sprayed on *B. cinerea* and *F. graminearum* agar plates, they were later observed within the cells of both fungi, proving that fungal cells are able to obtain RNAs directly from the environment [12,400,440]. Accordingly, inhibition of gray mold disease was achieved by spraying long dsRNAs targeting DCL1 and DCL2 of *B. cinerea* directly onto the surface of vegetables, flowers, and fruits [12]. Furthermore, these RNAs can be processed by fungal cells or even accumulate in host cells and then delivered into fungal ones through a cross-kingdom RNAi [392,393,441].

In *F. graminearum*, spray application of a long dsRNA on barley plants at a concentration of 1–20 ng mL^−1^ and targeting the three cytochrome P450 *lanosterol C-14α-demethylases (CYP3-dsRNA)* was shown to limit fungal growth. The effectiveness of silencing was demonstrated not only in sprayed leaves but also in non-treated distal parts in a detached leaf assay. The effective control of fungal infections by spray application in distal tissues required movement of dsRNA through the plant vascular system as well as the processing of dsDNA into siRNAs by fungal DCL1, following its uptake by the fungus. *F. graminearum* mutant for DCL1 was unable to fully perform a CYP3RNA-mediated SIGS, implying that the fungus uses RNAi to produce hindering siRNAs against its host. Therefore, it is possible that the fungus can take up environmental RNAs directly or indirectly through the plant cells, the host plant cleaves dsRNAs, and sRNAs are released to damage the pathogen [440].

In a study on the application of exogenous dsRNA to *S. sclerotiorum*, RNA sequencing data were used to select the dsRNA molecules targeting a single fungal gene [10]. Target genes were knocked down by topical applications of dsRNA, leading to reduced lesions on *Brassica napus*. In particular, the effect was evaluated using variable doses of dsRNA ranging from 100 to 1000 ng mL^−1^, and at least 200 ng mL^−1^ were required to induce significant gene silencing. It was shown that high doses failed to trigger transcript reduction, as previously demonstrated in other studies on flatworms and insects [443,444,445]. A high dose could saturate the RNAi silencing machinery, making it unable to process all the molecules at the same time [10]. Moreover, to achieve effective control of RNAi, the expression level of target genes was evaluated, since a high level of gene regulation may affect RNAi-based application because the dose of dsRNA may not be enough to eliminate all the transcripts of the target genes [10,446,447].

The first foliar application of dsRNA dates back to 2001, in a work by Tenllado et al. [448]. They used in vitro-produced dsRNA to test the feasibility of its interference in plant viral infections. They targeted three different viruses: the *Tobacco Etch Virus* (*TEV*), the *Pepper Mild Mottle Virus* (*PMMoV*), and the *Alfalfa Mosaic Virus* (*AMV*). In this study, the authors hypothesized that topical application of in vitro-produced dsRNA could be commercially applied in crop protection if dsRNA production was found to be inexpensive. Afterwards, they tried to reduce the cost of dsRNA production by using a crude extract of *E. coli* HT115. dsRNAs expressed by bacteria have been shown to be equally effective in protecting against plant viruses when sprayed on host plant surfaces [449].

One of the crucial points for the success of the RNAi tool is the choice of pathogenic genes to inactivate, with the aim of also minimizing off-target problems. Until now, some genes have been targeted for RNAi studies on *B. cinerea*, including effector genes, cell wall elongation genes, ergosterol and chitinase biosynthesis genes, genes of the vesicle trafficking pathway, and virulence genes involved in signal transduction or the secretory pathway [10,11,12,450,451,452]. Considering the sequence-dependent nature of RNAi, dsRNA molecules can be built with the aim of reducing possible side effects on non-target species. Moreover, the use of highly specific genes of the target pathogen is much less likely to negatively affect non-target organisms. In particular, targeting the virulence genes of the pathogen could be less risky and much more pathogen-specifically oriented than using essential pathogen genes.

### A Case Study: Down Regulation of Botrytis cinerea Virulence Genes Using Topical dsRNA Treatments to Control Gray Mold on Lettuce Leaves

According to a recent classification, virulence genes in *B. cinerea* can be divided into three classes: (i) the *sensu lato* virulence genes associated with the appressorium formation; (ii) the *sensu stricto* virulence genes according to the definition of Choquer et al. [453], and (iii) the plant cell wall disassembly genes (CAZyme genes) [23,453,454,455].

Spada and colleagues [36,63,456] selected three *sensu stricto B. cinerea* virulence genes: the two MAP kinase genes *BcBmp1* and *BcBmp3* involved in fungal pathogenesis, and the *BcPls1* tetraspanin gene related to appressorium penetration. The authors demonstrated that these genes were efficient and novel targets for RNAi against *B. cinerea*, and here some of the main results are presented.

In addition, in silico analysis using the si-Fi v21 software [457] confirmed that *BcBmp1*-, *BcBmp3*-, and *BcPls1*-dsRNA were highly specific for *B. cinerea*. Nevertheless, the best approach to reducing risks is to combine bioinformatics analyses with biological data [458]. In fact, when *BcBmp1*-, *BcBmp3*-, and *BcPls1*-dsRNA molecules were tested in silico and in vivo against *T. harzianum* (biocontrol agent) and *F. oxysporum* (endophyte), they did not give any negative results, and their high specificity was confirmed, in accordance with the prediction analysis [36,63].

The topical application of *BcBmp1*-dsRNA, *BcBmp3*-dsRNA, and *BcPls1*-dsRNA was shown to mediate both in vitro and in vivo the knockdown of their transcripts.

To validate the selected virulence genes, the *BcBmp1*-, *BcBmp3*-, and *BcPls1*-dsRNA molecules were in vitro applied to liquid cultures of *B. cinerea* grown in 96-well microtiter plates. This kind of assay is simple and cost-effective for the analysis of the inhibitory effects of molecules [459]. Furthermore, it allowed a small amount of dsRNA to be used for a preliminary evaluation of its outcome on fungal vegetative growth and conidia germination [460,461].

Fungal growth was not significantly delayed in the in vitro assay by *BcBmp1*-dsRNA treatments. By contrast, fungal growth was significantly delayed in the treatment with *BcBmp3*-dsRNA and *BcPls1*-dsRNA at 24, 48, and 72 h compared to the controls, and was subsequently restored at 96 h. The presence of gene knockdown does not appear to be directly linked to growth retardation. Indeed, the transcript levels at 48 h were remarkably reduced compared to controls [36,63].

Germination of *B. cinerea* conidia was reduced by approximately 50% after 6 h of incubation in the presence of *BcBmp3*-dsRNA compared to controls. Furthermore, some germination defects after 9 h of incubation were also observed [36].

Gray mold caused by *B. cinerea* is considered one of the main diseases in greenhouse-grown lettuce. The romaine lettuce variety and some iceberg lettuces are susceptible to *B. cinerea*, both in greenhouses and in the field [462].

**Figure 7:**
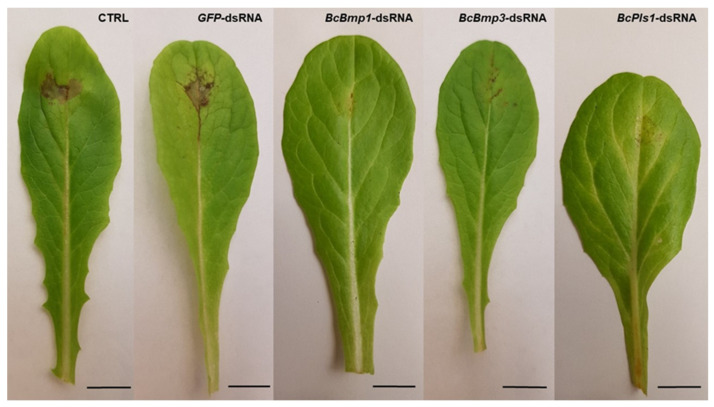
Infection symptoms of *B. cinerea* B05.10 on leaves of *Lactuca sativa* cv. Romana at 5 dpi. Leaves were treated with water + TE (CTRL), *GFP*-dsRNA, *BcBmp1*-dsRNA, *BcBmp3*-dsRNA, or *BcPls1*-dsRNA and then artificially inoculated with 5 µL of a conidial suspension (500 conidia) of the pathogen. Scale bars = 1 cm. Modified by Spada et al. [36,63].

**Figure 8:**
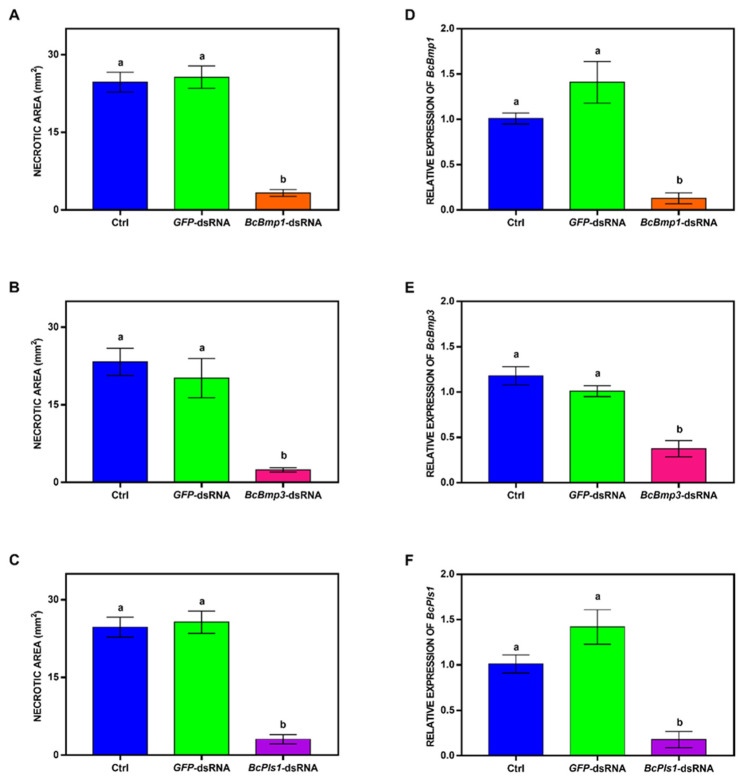
Necrotic areas (mm^2^) at 5 dpi caused by *B. cinerea* B05.10 were measured using the ImageJ software (version 1.53a). Leaves were treated with water + TE (CTRL, blue), *GFP*-dsRNA (green), or (**A**) *BcBmp1*-dsRNA (orange), (**B**) *BcBmp3*-dsRNA (fuchsia), and (**C**) *BcPls1*-dsRNA (purple) and were then artificially inoculated with a conidial suspension (5 × 10^2^ spores) of the pathogen. The graph shows the mean (±SE) values of two independent experiments. Relative transcript values of (**D**) *BcBmp1*-dsRNA (orange), (**E**) *BcBmp3*-dsRNA (fuchsia), and (**F**) *BcPls1*-dsRNA (purple) were calculated at 5 dpi by qRT-PCR using *GFP*-dsRNA (green) and CTRL (blue) as reference samples and normalized to the *GAPDH* gene of *Lactuca sativa* (*LsGAPDHR1*). The graph shows the mean (±SE) values. The same letters above the bars indicate no significant differences from each other (ANOVA) according to Tukey’s HSD test (*p* ≤ 0.05). Modified by Spada et al. [36,63].

In *Lactuca sativa* cv. Romana, Spada et al. [36,63] performed a detached leaf assay by applying a conidial suspension of the pathogen as inoculum after locally treating lettuce leaves with dsRNAs. At 5 dpi, topical application of *BcBmp1*-, *BcBmp3*-, and *BcPls1*-dsRNA molecules reduced lesion areas approximately eight to ten times compared to the controls (Figure 7). This strong decrease in necrotic areas (Figure 8A–C) was linked to a drastically reduced level of *Bmp1*, *Bmp3*, and *Pls1* transcripts in the infected lettuce leaves (Figure 8D–F).

From the perspective of a practical application, the instability of naked dsRNA sprayed on plants is an important point, and nanocarriers for dsRNA delivery can be used to extend its stability and durability. In line with this aim, Spada et al. [456] loaded *BcBmp3*-dsRNA on non-toxic, degradable, layered double hydroxide clay nanosheets (LDH) for a sustained release on leaf surface under in vivo conditions. Lettuce plants were sprayed with water, LDH, or *BcBmp3*-dsRNA/LDH complex on day 0. *B. cinerea* was inoculated using a conidial suspension 7 days after spray treatment. A significant reduction in gray mold severity was observed as assessed by a rating scale and by calculating the McKinney index.

Therefore, all *BcBmp1-*dsRNA, *BcBmp3-*dsRNA, and *BcPls1-*dsRNA showed high efficacy in RNAi against the corresponding genes by exogenous application of in vitro-produced dsRNA molecules [36,63]. These dsRNAs are highly specific for *B. cinerea* and are related to functions involved in the pathogenicity/virulence of the fungus, showing the potential for being used in SIGS experiments on whole lettuce plants.

## 7. Perspectives on Spray-Induced Gene Silencing (SIGS) as a New Strategy for Plant Disease Control

In recent years, the use of topical application of dsRNA has been investigated for its applications in plant protection. In particular, the dsRNA spraying technique has proven to be a valid delivery method as demonstrated in many studies concerning RNAi-based crop protection strategies [6,9,12,440,450,463,464,465,466,467,468,469,470].

However, this biotechnological approach needs to overcome some aspects before being transformed into practical applications. Indeed, dsRNA molecules are susceptible to degradation when exposed to the environment by applying them on the surface of plants or fruits. One of the studied approaches to increase the stability and longevity of naked dsRNAs for topical applications is to complex them with biocompatible nanoparticles [471,472].

SIGS-based disease management strategies require systems that can rapidly produce large quantities of dsRNA molecules and are cost-effective. Classic strategies for dsRNA production based on chemical synthesis or in vitro transcription are not feasible on large-scale applications due to high costs and low yields.

Furthermore, products resulting from this technology are currently undergoing risk assessment studies to verify their safety for the environment and the consumer [458].

### 7.1. dsRNA Production

The production of dsRNAs can be performed in vitro or in vivo [12,440,449,473,474].

In vitro production can be carried out by enzymatic transcription or chemical synthesis. Enzymatic transcription allows the synthesis of pure dsRNA and is based on the annealing of sense and antisense single-stranded RNAs. Enzymatic transcription is the principle on which commercially available kits are based and involves the use of DNA templates and PCR-generated ones, for the production of dsRNA [8]. This method has been widely used to target several *B. cinerea* genes through topical applications (Table 2). However, when a huge amount of dsRNA is needed for a large-scale application, these kits are too expensive [440,475].

Chemical synthesis can allow the production of a large amount of dsRNA, but it is much more expensive and also considerably longer as the length of the dsRNA produced increases [476]. However, chemical synthesis of siRNA allows not only to control the quantity and purity but also improves the stability of sRNAs through chemical modifications or labeling of sRNAs for localization by fluorescence microscopy [477].

The most suitable alternative is the production of dsRNAs using bacteria and yeasts as biofactories. In recent years, this option has lowered production costs, making the RNAi technique competitive on the market.

The in vivo production of dsRNA involves the use of yeast such as *Yarrowia lipolytica* or bacteria such as *Pseudomonas syringae* or *Escherichia coli* [478,479]. These methods allow for the synthesis of larger quantities of dsRNA at lower costs when a large amount is needed for field or greenhouse experiments [449,480,481].

RNase III-deficient *E. coli* strain HT115 (DE3) has been widely used in exogenous dsRNA application studies (Table 2). This strain contains the pro-phage λDE3, which encodes for the isopropyl β-D-1-thiogalactopyranoside (IPTG) inducible T7 polymerase gene required for dsRNA transcription [449,474]. Bacteria-expressed dsRNAs have been successfully used in fungi [11], viruses [482], worms [483], and insects [484].

In *B. cinerea*, dsRNA production mediated by *E. coli* HT115 (DE3) was used to target the *BcSas1* gene involved in secretory pathways and virulence of *B. cinerea*, resulting in a remarkable reduction in the size of lesions caused by the pathogen [449]. Similarly, *E. coli* strain HT115 (DE3) has been used to successfully produce dsRNA against *B. cinerea* in different delivery methods such as petiole adsorption, high-pressure spraying, and post-harvest spraying [11].

However, the use of *E. coli* lysate containing the dsRNA still poses controversy as it may contain bacterial residues with possible damage to the environment or human health [485,486]. Alternatively, new protocols for the isolation and purification of bacterially expressed dsRNA are needed to overcome health or pollution concerns. In fact, the dsRNA produced using bacteria as a biofactory cannot be secreted directly outside the cell. Therefore, many lysis, extraction, and purification steps are required to obtain dsRNA. Anyway, these processes are quite cumbersome and need to be further optimized [487]. In this regard, platforms have recently been developed to produce high quantities of purified dsRNA at low costs for use in gene silencing experiments [488,489].

The bacterium *P. syringae*, which contains the bacteriophage phi6 RNA-dependent RNA polymerase complex, seems a profitable organism to use in dsRNA production for large-scale application targeting different fungal and viral pathogens, as well as insect pests [490,491,492].

The yeast *Y. lipolytica* is gaining interest for its use in the production of dsRNA in vivo since it does not produce toxins and poses no risks to either health or the environment [478].

### 7.2. dsRNA Formulation

Another important aspect to take into consideration, along with the amount of dsRNA produced, is the stability of RNA molecules in environmental conditions, especially from the perspective of greenhouse and/or field trial applications. To prolong dsRNA stability, it can be incorporated into nanoparticles designed for its delivery to target organisms.

Chitosan, for instance, has been exploited to generate nanoparticles for dsRNA and siRNA delivery [493], and formulations including chitosan showed enhanced stability of sRNAs in diverse insects [494] and fungi such as *B. cinerea* [495] (Table 2) and *Rhizoctonia solani* [496].

Another tool has been proposed, which consists of using layered double hydroxide (LDH) clay nanosheets. These nanoparticles, being positively charged, can electrostatically bind oppositely charged dsRNA and protect it from nucleases and environmental conditions [497]. It has been shown that it is possible to load dsRNAs into LDH clay nanosheets to form a complex called BioClay^TM^, and when this is applied to the plant surface, there is a controlled release of the dsRNA sustained for up to 20 days. In the study, the efficacy of a topical spray of BioClay^TM^ was compared with that of naked dsRNA in providing plant protection against viruses [497].

The potential of this strategy was also evaluated in *B. cinerea* (Table 2). In particular, the dsRNA-LDH complex has been used to control postharvest decay caused by *B. cinerea*. First, it was shown that when fruits were treated with LDH alone, there was reduced decay development, which may have been due to the physical barrier produced by LDH itself [451]. Furthermore, LDH clay nanosheets prolonged the efficiency of dsRNA in the dsRNA-LDH complex for six weeks on fruits [451] and up to 3–4 weeks on tomato leaves and chickpea plants [498]. Conversely, naked dsRNA showed a marked weakening during fruit storage along with a reduced ability to control gray mold development [451,498]. In any case, when *B. cinerea* infection was achieved one week after treatment, the naked dsRNA showed better results compared to the LDH-dsRNA complex. This suggests that the ready availability of free dsRNA early in infection enhances protection against the pathogen compared to non-readily available dsRNA in the LDH-dsRNA complex [451,497].

In a recent study, lipid-based nanovesicles can be synthesized and used to deliver dsRNA against *B. cinerea* [499] (Table 2), as their use for drug delivery in human fungal pathogens has been previously demonstrated. These lipid-based nanovesicles increase the durability of dsRNA protection against *B. cinerea* in different plant products [500,501].

The great potential of SIGS in providing protection to plants against pathogens requires, as its counterpart, the fine-tuning of many aspects. First, a crucial prerequisite is the susceptibility of the target organism to environmental RNAi and therefore its uptake efficiency, since not all organisms behave in the same way [502]. Thus, in the setup of a large-scale application, the availability of a wide amount of interfering RNA is required [449,480] along with a formulation capable of ensuring a longer shelf life of the produced RNAi molecules [497,499]. Finally, the delivery method used is another important aspect, and different means of application have been adopted, such as trunk injection, high-pressure spraying, petiole absorption, soil application, root soaking, postharvest spraying, etc., and they may differ depending on the system studied [8]. To develop dsRNA-based products, all these factors must be taken into consideration, and in the near future, it is expected that the use of topical applications of dsRNA complexed with nanoparticles will be widely used in crop protection, as demonstrated by the growing interest both in academia and in the commercial sector [480]. In parallel, the risk assessment of this technology needs to be undertaken along with the implementation of existing legislation to favor the approval of these new dsRNA-based plant protection products [503].

**Table 2:** Silencing of *Botrytis cinerea* genes using dsRNA for plant disease control.

Target Gene(s)	Gene Function(s)	Host(s)	Production of dsRNA	Application of dsRNA	Delivery of dsRNA	Silencing Results	References
*DCL1*, *DCL2*, *DCL1+DCL2*	Endoribonucleas involved in the RNAi process	Tomato, Strawberry, Grape berries, Lettuce, Onion, Rose	In vitro ^a^	Dropped onto the surface of detached plant/fruit samples	Naked	Reduction in lesion size	[12,450]
BC1G_04955, 04775, 01592, 07805, 10306		Canola	In vitro ^a^	Dropped onto the surface of detached leaves	Naked	Reduction in lesion size	[10]
*β2-tubulin*	Fungal growth	Cucumber	In vitro ^a^	Sprayed onto the surface of micro-wounded plant leaves	Naked	Inhibition of spore germination and mycelial growth	[504]
*TOR*	Ser/Thr protein kinase	Potato, tomato	In transgenic plants (HIGS)		Transgenic leaves or fruits expressing dsRNA	Reduction in lesion size	[505]
*CYP51*+*chs1*+*EF2*	Lanosterol 14α demethylase, chitin synthase, elongation factor 2	Grape	In vivo ^b^	High pressure spraying on plant leaves and detached berry bunches, Leaf petioles adsorption	Naked	Reduction of disease symptoms	[11]
*Chs3a*, *Chs3b*, *DCL1*, *DCL2*, *Chs3a+Chs3b*, *DCL1+DCL2*	Chitin synthase, endoribonuclease is involved in the RNAi process	Strawberry	In *Escherichia coli* minicells (ME-dsRNA)	Sprayed onto the surface of fruits in greenhouse conditions	Encapsulated in minicells	Reduction in lesion size	[7]
*VPS51+DCTN1+SAC1* *DCL1+DCL2*	Vesicle trafficking pathway, Endoribonuclease is involved in the RNAi process	Grape berries, Tomato, Lettuce, Rose, Tomato	In vitro ^a^	Dropped onto the surface of detached plant/fruit samples, Sprayed on of intact plants	Naked	Reduction in lesion size	[502]
*sas1*	Rab/GTPase involved in secretory pathways	*Nicotiana benthamiana*	In vivo ^b^	Dropped onto the surface of detached leaves	Naked (living or lysed bacterial cells)	Reduction in lesion size	[452]
*erg11+erg1+erg13*	Ergosterol biosynthesis	Bell pepper, Cherry, Mango, Grape berries	In vitro ^a^	Sprayed or dropped onto the surface of wounded, detached fruits	Naked	Reduction in lesion size and AUDPC	[450]
*BcBmp1*, *BcBmp3*, *BcPls1*	MAP Kinases, Tetraspanin	Lettuce	In vitro ^a^	Dropped onto the surface of detached leaves	Naked	Reduction in lesion size	[36,63]
*BcBmp3*	MAP Kinase	Lettuce	In vitro ^a^	Sprayed on intact plants	MgAl LDH nanoparticles	Reduction of disease severity	[456]
*erg11+erg1+erg13*	Ergosterol biosynthesis	Cherry, Grape berries	In vitro ^a^	Sprayed onto the surface of wounded, detached fruits	MgAl LDH nanoparticles	Reduction of decay severity	[451]
*DCL1*+*DCL2*, *VPS51*+*DCTN1*+*SAC1*	Endoribonucleasinvolved in the RNAi process, Vesicle trafficking pathway	Chickpea plants, Tomato plants, Tomato fruits	Genolution (Korea) Agriculture Grade 2 service	Sprayed on intact plants, Sprayed on intact plants and detached leaves inoculated, Dropped onto the surface of detached fruits	MgAl and MgFe LDH nanoparticles (BioClay™)	Reduction of disease severity, Reduction in lesion size	[498]
*VPS51*/*DCTN1*/*SAC1*	Vesicle trafficking pathway	Lettuce, Rose, Tomato, Grape berries, *Arabidopsis thaliana*, Grape	In vitro ^a^	Dropped onto the surface of detached plant/fruit samplesSprayed onto the surface of detached leaves	Artificial nanovesicles (AVs)	Reduction in lesion size	[499]

^a^ MEGA script^®^RNAi Kit, ^b^ HT115 (DE3) *E. coli* cells.

### 7.3. Potential Risks of Treatments with dsRNA-Based Products

The risks associated with the application of dsRNA for crop protection are essentially of two types: impacts on human health and on the environment.

As regards human health, it includes both the end consumers of the treated products and the operators who distribute dsRNA topically. Exposure can occur primarily through ingestion and, to a lesser extent, through dermal exposure and inhalation. Humans have always consumed significant amounts of dsRNA virus-infected plant material without any indication of detectable effects. This is probably due to the rapid degradation of nucleic acids by many human biological barriers [506,507]. Accidental exposure through the skin or by inhalation can be minimized by using personal protective equipment [507].

Furthermore, the applied dsRNA sequence or siRNAs derived from it must have sufficient homology with endogenous transcripts to induce their degradation. The hybridization-dependent off-target gene suppression seems to be several orders of magnitude lower than the on-target gene suppression. Changes in transcriptional profiles have not been shown to impact in vivo safety in preclinical studies [508].

Regarding the environment in its broadest sense, the situation seems to be more complex and less studied, probably because scientific acquisitions in this area of research are very recent. In this case, the choice of the target sequence for silencing is a crucial issue. RNAi-based technologies require the fine-tuning of many factors to achieve effective pathogen control. In the design phase, a crucial step is the choice of the target gene to be silenced, to specifically target the pathogen of interest while avoiding the problems of non-target organisms. If the dsRNA sequence of a key gene is sufficiently homologous to non-target organisms, they could suffer the effects of silencing. As target sequences become less conserved, the likelihood of inducing deleterious off-target effects is reduced, owing to an inability to produce sufficient off-target homologous siRNAs [458].

dsRNA-based products are not considered GMOs unless they contain genetically modified organisms (e.g., bacteria transformed for the in vivo production of dsRNA). There is not yet a specific regulatory category for dsRNA-based products. However, it is interesting to point out that in the EU, dsRNAs are considered chemical pesticides; in the USA, they are biochemical pesticides; and in Australia, they are considered agricultural chemicals. Recently, the Organization for Economic Co-operation and Development (OECD) has produced guidance documents on the risk assessment of dsRNA products related to the environment and human health [485,509]. Well-defined, perhaps tailor-made, risk assessment protocols may be needed to comprehensively evaluate several factors (e.g., formulations, epigenetic modifications, fate of dsRNA in soil, surface water and sediment, leaf tissues, trophic chain exposure, off-target and non-target organisms) involved in the use of dsRNA-based products [509].

## 8. Conclusions

In this review, we have examined different aspects of *Botrytis cinerea*, which poses a serious threat to plants. The great capacity of the pathogen to cause serious product losses in countless crop species stimulates progress in the study of the interaction between *B. cinerea* and its hosts at the level of morphological, physiological, biochemical, and molecular analyses. The resulting picture is complex, and we emphasize that the availability of mutants for both the pathogen and the host could be the key to arriving at clearer results. For example, mutant characterization can facilitate clarification on the specific role of a virulence gene on the one hand or, conversely, on the role of a particular susceptibility gene on the other.

In line with this, very important hormonal aspects inherent to plant immunity have often been deciphered/validated using mutants for biosynthesis or susceptibility. Undoubtedly, today modern genome editing tools can facilitate the obtaining of ad hoc mutants [510,511].

As recently highlighted by Singh et al. [512], it is important to note that there is still a lot of work to be done if it is true that the function of 95% of *B. cinerea* genes is unknown. Furthermore, the polygenic nature of virulence and host specificity represent a further complication, and we must consider other important aspects: this necrotroph has a wide genetic diversity [512], and the levels and mechanisms of virulence may be influenced by the specific isolate analyzed.

As regards the applicative aspects, it is interesting that the study of the host-pathogen interaction has contributed to delineating a series of defence strategies against *B*. *cinerea*, which must necessarily be as least dependent as possible on the more traditional synthetic chemical products. The more we know the pathogenesis mechanisms implemented by *B. cinerea*, the more we understand that the attack occurs through sophisticated and diverse mechanisms [25].

Fundamental data have been collected over the last 10 years from the work of Weiberg and colleagues [378]. They found that sRNAs (RNA effectors) derived from *B. cinerea* are transmitted to host plants during infection to disrupt host immunity. Soon after, it became clear that RNA exchange was bidirectional because the host could also send molecules to the pathogen to interfere against virulence genes [513]. Therefore, trans-kingdom RNA silencing in plant-fungal pathogen interactions has provided the general framework within which to develop a new category of RNA-based fungicides [376]. This perspective, in fact, has aroused great interest from scholars, as demonstrated by the numerous reviews published on this topic in recent years [364,433,467,468,470,514,515,516,517,518].

In this context, our contribution aimed to limit the specific interest in gene silencing strategies to counteract the aggression of *B. cinerea* on plants. *B*. *cinerea* is a pathogen that is among the species that can be affected by exogenous dsRNA, precisely because this fungus is particularly efficient at absorbing exogenous dsRNA [502]. From the results summarized in Table 2, it is clear that dsRNAs (mostly delivered as naked molecules) were designed for *B*. *cinerea* genes involved in different metabolic pathways. Only in a few cases have disease control experiments been carried out using dsRNA treatments on whole plants or, better yet on plants directly in the field. Furthermore, the dsRNA production system almost exclusively involves in vitro synthesis using molecular biology kits, and to date, this aspect represents a limitation because their cost is not sustainable for in vivo applications on plants. At present, no specific studies have been conducted to evaluate whether the level of crop protection obtainable with the use of dsRNA is comparable/better than that obtainable with modern synthetic fungicides, and, in this regard, the preliminary results obtained by Duanis-Assaf et al. [450] are valuable. In any case, the numerous proof-of-concept studies are demonstrating the great potential of RNAi-based plant protection against *B. cinerea*, especially GMO-free RNA sprays. These results indicate that the way is open to acquiring new knowledge that can advance research with field applications and the formulation of dsRNA-based products.

## 9. Perspectives

In light of the above considerations, the development of RNAi-based fungicides will require further studies to achieve broad applicability, and in the meantime, it is desirable to progress on the validation of other unconventional strategies such as autophagy [519], defence priming [520], nanomaterials [521], hormonal treatments [522], amino acids [523], and antimicrobial peptides [524].

In a recent review of the mechanisms underlying plant defence responses to *B. cinerea*, Li and Cheng [525], identified transcriptional regulation and hormone transduction pathways as key aspects of plant immunity to counteract the pathogen. The authors identified specific proteins, hoping for advances in selection for resistance in the host as well as the use of elicitors and microorganisms. The effectiveness of these options will need to be verified soon.

Furthermore, unexpected molecules for the control of *B*. *cinerea* could become an additional weapon in the near future, as recent findings regarding carotenoids suggest [194,526]. Likewise, the spectrum of beneficial microorganisms against *B. cinerea* will also need to be investigated more thoroughly [527].

Among the most important challenges for the future, it is of fundamental interest to maintain the silencing effect on the target gene over time for as long as possible. Probably, to achieve the goal, the identification and characterization of the best possible material for complex dsRNAs could represent a very interesting area of research, and interdisciplinary collaboration among different scientists could be useful [472,528].

Moreover, it is very intriguing to point out that an innovative strategy for dsRNA delivery could be the use of mycorrhizae, especially in a forest environment (reviewed in [470]). Obviously, if these scenarios of unconventional solutions for dsRNA delivery are developed in the coming years, more and more attention will have to be paid to testing for unwanted silencing effects.

On the other hand, although techniques such as RNAi tend to be more politically acceptable than the direct use of GMOs, the ecological and health aspects of dsRNAs released into the environment require further study [529].

Technical advances in the production and formulation of dsRNAs to improve their efficacy, stability, and persistence could allow us to really consider their use as “RNAi-based biofungicides” of high commercial interest [6,8,479,480]. In the near future, RNAi can be considered a promising strategy due to its potential for environmentally friendly control of *B. cinerea* as well as other economically important plant pathogenic fungi and pests.

Finally, regarding RNAi strategies, a question that would be interesting to answer is whether these solutions can be implemented in the integrated management of the disease by evaluating any advantages or disadvantages of this approach.

## Data Availability

Data are contained within the article.

## References

[1] Moore D., Robson G.D., Trinci A.P.J. (2011). 21st Century Guidebook to Fungi.

[2] Eskola M., Kos G., Elliott C.T., Hajslova J., Mayar S., Krska R. (2020). Worldwide contamination of food-crops with mycotoxins: Validity of the widely cited ‘FAO estimate’ of 25%. Crit. Rev. Food Sci. Nutr..

[3] Chakraborty S., Newton A.C. (2011). Climate change, plant diseases and food security: An overview. Plant Pathol..

[4] Droby S., Wisniewski M., Macarisin D., Wilson C. (2009). Twenty years of postharvest biocontrol research: Is it time for a new paradigm?. Postharvest Biol. Technol..

[5] Dean R., van Kan J.A.L., Pretorius Z.A., Hammond-Kosack K.E., Di Pietro A., Spanu P.D., Rudd J.J., Dickman M., Kahmann R., Ellis J. (2012). The top 10 fungal pathogens in molecular plant pathology. Mol. Plant Pathol..

[6] Islam T.M., Sherif S. (2020). RNAi-based biofungicides as a promising next-generation strategy for controlling devastating gray mold diseases. Int. J. Mol. Sci..

[7] Islam M.T., Davis Z., Chen L., Englaender J., Zomorodi S., Frank J., Bartlett K., Somers E., Carballo S.M., Kester M. (2021). Minicell-based fungal RNAi delivery for sustainable crop protection. Microbial. Biotechnol..

[8] Gebremichael D.E., Haile Z.M., Negrini F., Sabbadini S., Capriotti L., Mezzetti B., Baraldi E. (2021). RNA interference strategies for future management of plant pathogenic fungi: Prospects and challenges. Plants.

[9] Chakraborty P., Ghosh A. (2022). Topical spray of dsRNA induces mortality and inhibits Chilli Leaf Curl Virus transmission by *Bemisia tabaci* Asia II 1. Cells.

[10] McLoughlin A.G., Wytinck N., Walker P.L., Girard I.J., Rashid K.Y., de Kievit T., Fernando W.G.D., Whyard S., Belmonte M.F. (2018). Identification and application of exogenous dsRNA confers plant protection against *Sclerotinia sclerotiorum* and *Botrytis cinerea*. Sci. Rep..

[11] Nerva L., Sandrini M., Gambino G., Chitarra W. (2020). Double-stranded RNAs (dsRNAs) as a sustainable tool against gray mold (*Botrytis cinerea*) in grapevine: Effectiveness of different application methods in an open-air environment. Biomolecules.

[12] Wang M., Weiberg A., Lin F.M., Thomma B.P., Huang H.D., Jin H. (2016). Bidirectional cross-kingdom RNAi and fungal uptake of external RNAs confer plant protection. Nat. Plants.

[13] Persoon C.H. (1794). Dispositio Methodica Fungorum in Classes, Ordines, Familias et Genera.

[14] de Bary A. (1866). Morphologie und Physiologie der Pilze, Flechten und Myxomyceten.

[15] Gregory P.H. (1949). Studies on *Sclerotinia* and *Botrytis*: II. De Bary’s description and specimens of *Peziza fuckeliana*. Trans. Br. Mycol. Soc..

[16] Mirzaei S., Goltapeh E.M., Shams-Bakhsh M., Safaie N. (2008). Identification of *Botrytis* spp. on plants grown in Iran. J. Phytopathol..

[17] Elad Y., Fillinger S., Elad Y. (2016). Cultural and Integrated Control of *Botrytis* spp.. Botrytis—The Fungus, the Pathogen and Its Management in Agricultural Systems.

[18] Zhong S., Zhang J., Zhang G.Z. (2019). *Botrytis polyphyllae*: A new *Botrytis* species causing gray mold on *Paris polyphylla*. Plant Dis..

[19] Elad Y., Pertot I., Cotes Prado A.M., Stewart A., Fillinger S., Elad Y. (2016). Plant hosts of *Botrytis* Spp.. Botrytis—The Fungus, the Pathogen and Its Management in Agricultural Systems.

[20] Elad Y., Vivier M., Fillinger S., Fillinger S., Elad Y. (2016). *Botrytis*, the good, the bad and the ugly. Botrytis—The Fungus, the Pathogen and Its Management in Agricultural Systems.

[21] van Kan J.A.L. (2003). Infection strategies of *Botrytis cinerea*. VIII International Symposium on Postharvest Physiology of Ornamental Plants.

[22] Williamson B., Tudzynski B., Tudzynski P., van Kan J.A.L. (2007). *Botrytis cinerea*: The cause of grey mould disease. Mol. Plant Pathol..

[23] Amselem J., Cuomo C.A., van Kan J.A.L., Viaud M., Benito E.P., Couloux A., Coutinho P.M., de Vries R.P., Dyer P.S., Fillinger S. (2011). Genomic analysis of the necrotrophic fungal pathogens *Sclerotinia sclerotiorum* and *Botrytis cinerea*. PLoS Genet..

[24] Cheung N., Tian L., Liu X., Li X. (2020). The destructive fungal pathogen *Botrytis cinerea*-insights from genes studied with mutant analysis. Pathogens.

[25] Bi K., Liang Y., Mengiste T., Sharon A. (2023). Killing softly: A roadmap of *Botrytis cinerea* pathogenicity. Trends Plant Sci..

[26] Fillinger S., Walker A.S., Fillinger S., Elad Y. (2016). Chemical control and resistance management of *Botrytis* diseases. Botrytis—The Fungus, the Pathogen and Its Management in Agricultural Systems.

[27] Nishimoto R. (2019). Global trends in the crop protection industry. J. Pestic. Sci..

[28] van Kan J.A.L., Stassen J.H., Mosbach A., Van Der Lee T.A., Faino L., Farmer A.D., Papasotiriou D.G., Zhou S., Seidl M.F., Cottam E. (2017). A gapless genome sequence of the fungus *Botrytis cinerea*. Mol. Plant Pathol..

[29] Beever R.E., Weeds P.L., Elad Y., Williamson B., Tudzynski P., Delen N. (2007). Taxonomy and genetic variation of *Botrytis* and *Botryotinia*. Botrytis: Biology, Pathology and Control.

[30] Da Silva Ripardo-Filho H., Coca Ruíz V., Suárez I., Moraga J., Aleu J., Collado I.G. (2023). From genes to molecules, secondary metabolism in *Botrytis cinerea*: New insights into anamorphic and teleomorphic stages. Plants.

[31] De Miccolis Angelini R.M., Pollastro S., Faretra F., Fillinger S., Elad Y. (2016). Genetics of *Botrytis cinerea*. Botrytis—The Fungus, the Pathogen and Its Management in Agricultural System.

[32] Fukumori Y., Nakajima M., Akutsu K. (2004). Microconidia act the role as spermatia in the sexual reproduction of *Botrytis cinerea*. J. Gen. Plant Pathol..

[33] Holz G., Coertze S., Williamson B., Elad Y., Williamson B., Tudzynski P., Delen N. (2007). The ecology of *Botrytis* on plant surfaces. Botrytis: Biology, Pathology and Control.

[34] Prins T.W., Tudzynski P., Von Tiedemann A., Tudzynski B., ten Have A., Hansen M.E., Tenberge K., van Kan J.A.L., Kronstad J.W. (2000). Infection strategies of *Botrytis cinerea* and related necrotrophic pathogens. Fungal Pathology.

[35] Petrasch S., Knapp S.J., van Kan J.A., Blanco-Ulate B. (2019). Grey mould of strawberry, a devastating disease caused by the ubiquitous necrotrophic fungal pathogen *Botrytis cinerea*. Mol. Plant Pathol..

[36] Spada M., Pugliesi C., Fambrini M., Pecchia S. (2021). Silencing of the Slt2-type MAP kinase *Bmp3* in *Botrytis cinerea* by application of exogenous dsRNA affects fungal growth and virulence on *Lactuca sativa*. Int. J. Mol. Sci..

[37] Prusky D. (1996). Pathogen quiescence in post-harvest diseases. Annu. Rev. Phytopathol..

[38] Jarvis W.R. (1962). The dispersal of spores of *Botrytis cinerea* Fr. in a raspberry plantation. Trans. Br. Mycol. Soc..

[39] Fermaud M., Gaunt R.E. (1995). *Thrips obscuratus* as a potential vector of *Botrytis cinerea* in kiwifruit. Mycol. Res..

[40] Louis C., Girard M., Kuhl G., Lopez Ferber M. (1996). Persistence of *Botrytis cinerea* in its vector *Drosophila melanogaster*. Phytopathology.

[41] Doss R.P., Potter S.W., Chastagner G.A., Christian J.K. (1993). Adhesion of nongerminated *Botrytis cinerea* conidia to several substrata. Appl. Environ. Microbiol..

[42] Doss R.P., Potter S.W., Soeldner A.H., Christian J.K., Fukunaga L.E. (1995). Adhesion of germlings of *Botrytis cinerea*. Appl. Environ. Microbiol..

[43] Snow D. (1949). The germination of mould spores at controlled humidities. Ann. Appl. Biol..

[44] Williamson B., Duncan G.H., Harrison J.G., Harding L.A., Elad Y., Zimand G. (1995). Effect of humidity on infection of rose petals by dry-inoculated conidia of *Botrytis cinerea*. Mycol. Res..

[45] Cole L., Dewey F.M., Hawes C.R. (1996). Infection mechanisms of *Botrytis* species: Pre-penetration and pre-infection processes of dry and wet conidia. Mycol. Res..

[46] Edlich W., Lorenz G., Lyr H., Nega E., Pommer E.H. (1989). New aspects on the infection mechanism of *Botrytis cinerea* Pers. Neth. J. Plant Pathol..

[47] Govrin E.M., Levine A. (2000). The hypersensitive reaction facilitates plant infection by the necrotrophic fungus *Botrytis cinerea*. Curr. Biol..

[48] Akagi A., Dandekar A.M., Stotz H.U. (2011). Resistance of *Malus domestica* fruit to *Botrytis cinerea* depends on endogenous ethylene biosynthesis. Phytopathology.

[49] Fourie J.F., Holz G. (1995). Initial infection processes by *Botrytis cinerea* on nectarine and plum fruit and the development of decay. Phytopathology.

[50] Tenberge K.B., Elad Y., Williamson B., Tudzynski P., Delen N. (2007). Morphology and cellular organisation in *Botrytis* interactions with plants. Botrytis: Biology, Pathology and Control.

[51] Van den Heuvel J., Waterreus L.P. (1983). Conidial concentration as an important factor determining the type of prepenetration structures formed by *Botrytis cinerea* on leaves of French bean (*Phaseolus vulgaris*). Plant Pathol..

[52] Backhouse D., Willetts H.J. (1987). Development and infection cushions of *Botrytis cinerea*. Trans. Br. Mycol. Soc..

[53] Choquer M., Rascle C., Gonçalves I.R., de Vallée A., Ribot C., Loisel E., Smilevski P., Ferria J., Savadogo M., Souibgui E. (2021). The infection cushion: A fungal “weapon” of plant-biomass destruction. Environ. Microbiol..

[54] Gourgues M., Clergeot P.-H., Veneault C., Cots J., Sibuet S., Brunet-Simon A., Levis C., Langin T., Lebrun M.H. (2002). A new class of tetraspanins in fungi. Biochem. Biophys. Res. Commun..

[55] Clergeot P.H., Gourgues M., Cots J., Laurans F., Latorse M.P., Pépin R., Tharreau D., Notteghem J.L., Lebrun M.H. (2001). *PLS1*, a gene encoding a tetraspanin-like protein, is required for penetration of rice leaf by the fungal pathogen *Magnaporthe grisea*. Proc. Natl. Acad. Sci. USA.

[56] Hořejši V., Vlček Č. (1991). Novel structurally distinct family of leucocyte surface glycoproteins including CD9, CD37, CD53 and CD63. FEBS Lett..

[57] Boucheix C., Rubinstein E. (2001). Tetraspanins. Cell. Mol. Life Sci..

[58] Hemler M.E. (2001). Specific tetraspanin functions. J. Cell Biol..

[59] Yanez-Mo M., Mittelbrunn M., Sanchez-Madrid F. (2001). Tetraspanins and intercellular interactions. Microcirculation.

[60] Gourgues M., Brunet-Simon A., Lebrun M.H., Levis C. (2004). The tetraspanin BcPls1 is required for appressorium-mediated penetration of *Botrytis cinerea* into host plant leaves. Mol. Microbiol..

[61] Veneault-Fourrey C., Lambou K., Lebrun M.H. (2006). Fungal Pls1 tetraspanins as key factors of penetration into host plants: A role in re-establishing polarized growth in the appressorium?. FEMS Microbiol. Lett..

[62] Kars I., van Kan J.A.L., Elad Y., Williamson B., Tudzynski P., Delen N. (2007). Extracellular enzymes and metabolites involved in pathogenesis of *Botrytis*. Botrytis: Biology, Pathology and Control.

[63] Spada M., Pugliesi C., Fambrini M., Palpacelli D., Pecchia S. (2023). Knockdown of *Bmp1* and *Pls1* virulence genes by exogenous application of RNAi-inducing dsRNA in *Botrytis cinerea*. Int. J. Mol. Sci..

[64] Hou J., Feng H.-Q., Chang H.-W., Liu Y., Li G.-H., Yang S., Sun C.-H., Zhang M.-Z., Yuan Y., Sun J. (2020). The H3K4 demethylase Jar1 orchestrates ROS production and expression of pathogenesis-related genes to facilitate *Botrytis cinerea* virulence. New Phytol..

[65] Isenegger D.A., Ford R., Taylor P.W.J. (2011). Disease reaction of chickpea (*Cicer* spp.) genotypes to *Botrytis* grey mould (*Botrytis cinerea*). Australas. Plant Pathol..

[66] Doss R.P., Deisenhofer J., von Nidda H.A.K., Soeldner A.H., McGuire R.P. (2003). Melanin in the extracellular matrix of germlings of *Botrytis cinerea*. Phytochemistry.

[67] Rolke Y., Liu S., Quidde T., Williamson B., Schouten S., Weltring K.-M., Siewers V., Tenberge K.B., Tudzynski B., Tudzynski P. (2004). Functional analysis of H_2_O_2_-generating systems in *Botrytis cinerea*: The major Cu-Zn-superoxide dismutase (BCSOD1) contributes to virulence on French bean, whereas a glucose oxidase (BCGOD1) is dispensable. Mol. Plant Pathol..

[68] Mansfield J.W., Richardson A. (1981). The ultrastructure of interactions between *Botrytis* species and broad bean leaves. Physiol. Plant Pathol..

[69] Kars I., Krooshof G.H., Wagemakers L., Joosten R., Benen J.A., van Kan J.A.L. (2005). Necrotizing activity of five *Botrytis cinerea* endopolygalacturonases produced in *Pichia pastoris*. Plant J..

[70] Veloso J., van Kan J.A.L. (2018). Many shades of grey in *Botrytis*-host plant interactions. Trends Plant Sci..

[71] Cui F., Li X., Wu W., Luo W., Wu Y., Brosché M., Overmyer K. (2022). Ectopic expression of *BOTRYTIS SUSCEPTIBLE1* reveals its function as a positive regulator of wound-induced cell death and plant susceptibility to *Botrytis*. Plant Cell.

[72] Colmenares A.J., Aleu J., Duran-Patron R., Collado I.G., Hernandez-Galan R. (2002). The putative role of botrydial and related metabolites in the infection mechanism of *Botrytis cinerea*. J. Chem. Ecol..

[73] Cutler H.G., Jacyno J.M., Harwood J.S., Dulik D., Goodrich P.D., Roberts R.G. (1993). Botcinolide: A biologically active natural product from *Botrytis cinerea*. Biosci. Biotechnol. Biochem..

[74] Reino J.L., Hernandez-Galan R., Duran-Patron R., Collado I.G. (2004). Virulence-toxin production relationship in isolates of the plant pathogenic fungus *Botrytis cinerea*. J. Phytopathol..

[75] Manteau S., Abouna S., Lambert B., Legendre L. (2003). Differential regulation by ambient pH of putative virulence factor secretion by the phytopathogenic fungus *Botrytis cinerea*. FEMS Microbiol. Ecol..

[76] Tenberge K.B., Beckedorf M., Hoppe B., Schouten A., Solf M., von den Driesch M. (2002). In situ localization of AOS in host-pathogen interactions. Microsc. Microanal..

[77] Rivas S., Thomas C.M. (2005). Molecular interactions between tomato and the leaf mold pathogen *Cladosporium fulvum*. Annu. Rev. Phytopathol..

[78] Lyon G.D., Goodman B.A., Williamson B., Elad Y., Williamson B., Tudzynski P., Delen N. (2004). Botrytis cinerea perturbs redox processes as an attack strategy in plants. Botrytis: Biology, Pathology and Control.

[79] Segmüller N., Kokkelink L., Giesbert S., Odinius D., van Kan J.A.L., Tudzynski P. (2008). NADPH oxidases are involved in differentiation and pathogenicity in *Botrytis cinerea*. Mol. Plant Microbe Interact..

[80] Schouten A., van Baarlen P., van Kan J.A.L. (2008). Phytotoxic Nep1-like proteins from the necrotrophic fungus *Botrytis cinerea* associate with membranes and the nucleus of plant cells. New Phytol..

[81] Gijzen M., Nürnberger T. (2006). Nep1-like proteins from plant pathogens: Recruitment and diversification of the NPP1 domain across taxa. Phytochemistry.

[82] Frías M., González C., Brito N. (2011). BcSpl1, a cerato-platanin family protein, contributes to *Botrytis cinerea* virulence and elicits the hypersensitive response in the host. New Phytol..

[83] Leone G., Schoffelmeer E.A.M., Heuvel J.V.D. (1998). Purification and characterization of a constitutive polygalacturonase associated with the infection process of French bean leaves by *Botrytis cinerea*. Can. J. Bot..

[84] Movahedi S., Heale J.B. (1990). The roles of aspartic proteinase and endo-pectin lyase enzymes in the primary stages of infection and pathogenesis of various host tissues by different isolates of *Botrytis cinerea* Pers ex. Pers. Physiol. Mol. Plant Pathol..

[85] Reignault P., Mercier M., Bompeix G., Boccara M. (1994). Pectin methylesterase from *Botrytis cinerea*: Physiological, biochemical and immunochemical studies. Microbiology.

[86] Kars I., McCalman M., Wagemakers L., van Kan J.A.L. (2005). Functional analysis of *Botrytis cinerea* pectin methylesterase genes by PCR-based targeted mutagenesis: *Bcpme1* and *Bcpme2* are dispensable for virulence of strain B05.10. Mol. Plant Pathol..

[87] Valette-Collet O., Cimerman A., Reignault P., Levis C., Boccara M. (2003). Disruption of *Botrytis cinerea* pectin methylesterase gene *Bcpme1* reduces virulence on several host plants. Mol. Plant Microbe Interact..

[88] Wubben J.P., Mulder W., ten Have A., van Kan J.A., Visser J. (1999). Cloning and partial characterization of endopolygalacturonase genes from *Botrytis cinerea*. Appl. Environ. Microbiol..

[89] Johnston D.J., Williamson B. (1992). Purification and characterization of four polygalacturonases from *Botrytis cinerea*. Mycol. Res..

[90] Espino J.J., Brito N., Noda J., González C. (2005). *Botrytis cinerea* endo-β-1, 4-glucanase Cel5A is expressed during infection but is not required for pathogenesis. Physiol. Mol. Plant Pathol..

[91] González C., Brito N., Sharon A., Fillinger S., Elad Y. (2016). Infection process and fungal virulence factors. Botrytis—The Fungus, the Pathogen and Its Management in Agricultural Systems.

[92] Espino J.J., Gutiérrez-Sánchez G., Brito N., Shah P., Orlando R., González C. (2010). The *Botrytis cinerea* early secretome. Proteomics.

[93] ten Have A., Espino J.J., Dekkers E., Van Sluyter S.C., Brito N., Kay J., González C., van Kan J.A.L. (2010). The *Botrytis cinerea* aspartic proteinase family. Fungal Genet. Biol..

[94] Weiller F., Schückel J., Willats W.G.T., Driouich A., Vivier M.A., Moore J.P. (2021). Tracking cell wall changes in wine and table grapes undergoing *Botrytis cinerea* infection using glycan microarrays. Ann. Bot..

[95] AbuQamar S., Moustafa K., Tran L.S. (2017). Mechanisms and strategies of plant defense against *Botrytis cinerea*. Crit. Rev. Biotechnol..

[96] Castillo L., Plaza V., Larrondo L.F., Canessa P. (2017). Recent advances in the study of the plant pathogenic fungus *Botrytis cinerea* and its interaction with the environment. Curr. Protein Pept. Sci..

[97] Underwood W. (2012). The plant cell wall: A dynamic barrier against pathogen invasion. Front. Plant Sci..

[98] Blanco-Ulate B., Labavitch J.M., Vincenti E., Powell A.A.T., Conta D., Fillinger S., Elad Y. (2016). Hitting the Wall: Plant Cell Walls during *Botrytis cinerea* Infections. Botrytis—The Fungus, the Pathogen and Its Management in Agricultural Systems.

[99] De Leeuw G. (1985). Deposition of lignin, suberin and callose in relation to the restriction of infection by *B. cinerea* in ghost spots of tomato fruits. J. Phytopathol..

[100] German L., Yeshvekar R., Benitez-Alfonso Y. (2023). Callose metabolism and the regulation of cell walls and plasmodesmata during plant mutualistic and pathogenic interactions. Plant Cell Environ..

[101] Kabbage M., Williams B., Dickman M.B. (2013). Cell death control: The interplay of apoptosis and autophagy in the pathogenicity of *Sclerotinia sclerotiorum*. PLoS Pathog..

[102] Shlezinger N., Minz A., Gur Y., Hatam I., Dagdas Y.F., Talbot N.J., Sharon A. (2011). Anti-apoptotic machinery protects the necrotrophic fungus *Botrytis cinerea* from host-induced apoptotic-like cell death during plant infection. PLoS Pathog..

[103] Sharon A., Shlezinger N. (2013). Fungi infecting plants and animals: Killers, non-killers, and cell death. PLoS Pathog..

[104] Lai Z., Wang F., Zheng Z., Fan B., Chen Z. (2011). A critical role of autophagy in plant resistance to necrotrophic fungal pathogens. Plant J..

[105] Maule A.J., Ride J.P. (1976). Ammonia-lyase and O-methyl transferase activities related to lignification in wheat leaves infected with *Botrytis*. Phytochemistry.

[106] Bennett M.H., Gallagher M.D.S., Bestwick C.S., Rossiter J.T., Mansfield J.W. (1994). The phytoalexin response of lettuce to challenge by *Botrytis cinerea*, *Bremia lactucae* and *Pseudomonas syringae* pv. phaseolicola. Physiol. Mol. Plant Pathol..

[107] Díaz J., ten Have A., van Kan J.A.L. (2002). The role of ethylene and wound signalling in resistance of tomato to *Botrytis cinerea*. Plant Physiol..

[108] Langcake P. (1981). Disease resistance of *Vitis* spp. and the production of the stress metabolites resveratrol, ε-viniferin, α-viniferin and pterostilbene. Physiol. Plant Pathol..

[109] De Lorenzo G., D’Ovidio R., Cervone F. (2001). The role of polygalacturonase-inhibiting proteins (PGIPs) in defense against pathogenic fungi. Annu. Rev. Phytopathol..

[110] Cantu D., Vicente A.R., Labavitch J.M., Bennett A.B., Powell A.L. (2008). Strangers in the matrix: Plant cell walls and pathogen susceptibility. Trends Plant Sci..

[111] Manabe Y., Nafisi M., Verhertbruggen Y., Orfila C., Gille S., Rautengarten C., Cherk C., Marcus S.E., Somerville S., Pauly M. (2011). Loss of function mutation of *REDUCED WALL ACETYLATION2* in *Arabidopsis* leads to reduced cell wall acetylation and increased resistance to *Botrytis cinerea*. Plant Physiol..

[112] Osbourn A.E., Melton R.E., Wubben J.P., Flegg L.M., Oliver R.P., Daniels M.J., Kohmoto K., Yoder O.C. (1998). Saponin detoxification and fungal pathogenesis. Molecular Genetics of Host-Specific Toxins in Plant Disease.

[113] Tudzynski P., Tudzynski B. (1998). Genetics of plant pathogenic fungi. Progr. Bot..

[114] Pezet R., Pont V., Hoang-Van K. (1991). Evidence for oxidative detoxication of pterostilbene and resveratrol by a laccase-like stilbene oxidase produced by *Botrytis cinerea*. Physiol. Mol. Plant Pathol..

[115] Verhoeff K., Liem J.I. (1975). Toxicity of tomatine to *Botrytis cinerea*, in relation to latency. J. Phytopathol..

[116] Quidde T., Osbourn A.E., Tudzynski P. (1998). Detoxification of alpha-tomatine by *Botrytis cinerea*. Physiol. Mol. Plant Pathol..

[117] Quidde T., Büttner P., Tudzynski P. (1999). Evidence for three different specific saponin-detoxifying activities in *Botrytis cinerea* and cloning and functional analysis of a gene coding for a putative avenacinase. Eur. J. Plant Pathol..

[118] Schouten A., Tenberge K.B., Vermeer J., Stewart J., Wagemakers L., Williamson B., van Kan J.A.L. (2002). Functional analysis of an extracellular catalase of *Botrytis cinerea*. Mol. Plant Pathol..

[119] van Kan J.A.L. (2006). Licensed to kill: The lifestyle of a necrotrophic plant pathogen. Trends Plant Sci..

[120] Knogge W. (1996). Fungal infection of plants. Plant Cell.

[121] Bölker M. (1998). Sex and crime: Heterotrimeric G proteins in fungal mating and pathogenesis. Fungal Genet. Biol..

[122] Mitchell T.K., Dean R.A. (1995). The cAMP-dependent protein kinase catalytic subunit is required for appressorium formation and pathogenesis by the rice blast pathogen *Magnaporthe grisea*. Plant Cell.

[123] Xu X.-M., Harris D.C., Berrie A.M. (2000). Modeling infection of strawberry flowers by *Botrytis cinerea* using field data. Phytopathology.

[124] Schumacher J., Fillinger S., Elad Y. (2016). Signal transduction cascades regulating differentiation and virulence. Botrytis—The Fungus, the Pathogen and Its Management in Agricultural Systems.

[125] Wennerberg K., Rossman K.L., Der C.J. (2005). The Ras superfamily at a glance. J. Cell Sci..

[126] Schumacher J., Kokkelink L., Huesmann C., Jimenez-Teja D., Collado I.G., Barakat R., Tudzynski P., Tudzynski B. (2008). The cAMP-dependent signaling pathway and its role in conidial germination, growth, and virulence of the gray mold *Botrytis cinerea*. Mol. Plant Microbe Interact..

[127] Minz Dub A., Kokkelink L., Tudzynski B., Tudzynski P., Sharon A. (2013). Involvement of *Botrytis cinerea* small GTPases BcRAS1 and BcRAC in differentiation, virulence, and the cell cycle. Eukaryot. Cell.

[128] Gilman A.G. (1987). G proteins: Transducers of receptor-generated signals. Annu. Rev. Biochem..

[129] Hamm H.E., Gilchrist A. (1996). Heterotrimeric G proteins. Curr. Opin. Cell Biol..

[130] Cabrera-Vera T.M., Vanhauwe J., Thomas T.O., Medkova M., Preininger A., Mazzoni M.R., Hamm H.E. (2003). Insights into G protein structure, function, and regulation. Endocr. Rev..

[131] Liu S., Dean R.A. (1997). G protein α subunit genes control growth development and pathogenicity of *Magnaporthe grisea*. Mol. Plant Microbe Interact..

[132] Doehlemann G., Berndt P., Hahn M. (2006). Different signalling pathways involving a Gα protein, cAMP and a MAP kinase control germination of *Botrytis cinerea* conidia. Mol. Microbiol..

[133] Gronover C.S., Schumacher J., Hantsch P., Tudzynski B. (2005). A novel seven-helix transmembrane protein BTP1 of *Botrytis cinerea* controls the expression of GST-encoding genes, but is not essential for pathogenicity. Mol. Plant Pathol..

[134] Li L., Wright S.J., Krystofova S., Park G., Borkovich K.A. (2007). Heterotrimeric G protein signaling in filamentous fungi. Annu. Rev. Microbiol..

[135] Willardson B.M., Howlett A.C. (2007). Function of phosducin-like proteins in G protein signaling and chaperone-assisted protein folding. Cell. Signal..

[136] Nanni V., Schumacher J., Giacomelli L., Brazzale D., Sbolci L., Moser C., Tudzynski P., Baraldi E. (2014). VvAMP2, a grapevine flower-specific defensin capable of inhibiting *Botrytis cinerea* growth: Insights into its mode of action. Plant Pathol..

[137] Choi W., Dean R.A. (1997). The adenylate cyclase gene MAC1 of *Magnaporthe grisea* controls appressorium formation and other aspects of growth and development. Plant Cell.

[138] Sassone-Corsi P. (2012). The cyclic AMP pathway. Cold Spring Harb. Perspect. Biol..

[139] Klimpel A., Gronover C.S., Williamson B., Stewart J.A., Tudzynski B. (2002). The adenylate cyclase (BAC) in *Botrytis cinerea* is required for full pathogenicity. Mol. Plant Pathol..

[140] Harren K., Brandhoff B., Knödler M., Tudzynski B. (2013). The high-affinity phosphodiesterase BcPde2 has impact on growth, differentiation and virulence of the phytopathogenic ascomycete *Botrytis cinerea*. PLoS ONE.

[141] Zhao X., Mehrabi R., Xu J.R. (2007). Mitogen-activated protein kinase pathways and fungal pathogenesis. Eukaryot. Cell.

[142] Gustin M.C., Albertyn J., Alexander M., Davenport K. (1998). MAP kinase pathways in the yeast *Saccharomyces cerevisiae*. Microbiol. Mol. Biol. Rev..

[143] Xu J.R., Hamer J.E. (1996). MAP kinase and cAMP signaling regulate infection structure formation and pathogenic growth in the rice blast fungus *Magnaporthe grisea*. Genes Dev..

[144] Lev S., Sharon A., Hadar R., Ma H., Horwitz B.A. (1999). A mitogen-activated protein kinase of the corn leaf pathogen *Cochliobolus heterostrophus* is involved in conidiation, appressorium formation, and pathogenicity: Diverse roles for mitogen-activated protein kinase homologs in foliar pathogens. Proc. Natl Acad. Sci. USA.

[145] Takano Y., Kikuchi T., Kubo Y., Hamer J.E., Mise K., Furusawa I. (2000). The *Colletotrichum lagenarium* MAP kinase gene *CMK1* regulates diverse aspects of fungal pathogenesis. Mol. Plant Microbe Interact..

[146] Zheng L., Campbell M., Murphy J., Lam S., Xu J.R. (2000). The *BMP1* gene is essential for pathogenicity in the gray mold fungus *Botrytis cinerea*. Mol. Plant Microbe Interact..

[147] Schamber A., Leroch M., Diwo J., Mendgen K., Hahn M. (2010). The role of mitogen-activated protein (MAP) kinase signalling components and the Ste12 transcription factor in germination and pathogenicity of *Botrytis cinerea*. Mol. Plant Pathol..

[148] Levin D.E. (2005). Cell wall integrity signaling in *Saccharomyces cerevisiae*. Microbiol. Mol. Biol. Rev..

[149] Hou Z., Xue C., Peng Y., Katan T., Kistler H.C., Xu J.R. (2002). A mitogen-activated protein kinase gene (*MGV1*) in *Fusarium graminearum* is required for female fertility, heterokaryon formation, and plant infection. Mol. Plant Microbe Interact..

[150] Kojima K., Kikuchi T., Takano Y., Oshiro E., Okuno T. (2002). The mitogen-activated protein kinase gene *MAF1* is essential for the early differentiation phase of appressorium formation in *Colletotrichum lagenarium*. Mol. Plant Microbe Interact..

[151] Mehrabi R., van der Lee T., Waalwijk C., Kema G.H. (2006). *MgSlt2*, a cellular integrity MAP kinase gene of the fungal wheat pathogen *Mycosphaerella graminicola*, is dispensable for penetration but essential for invasive growth. Mol. Plant Microbe Interact..

[152] Xu J.R., Staiger C.J., Hamer J.E. (1998). Inactivation of the mitogen-activated protein kinase Mps1 from the rice blast fungus prevents penetration of host cells but allows activation of plant defense responses. Proc. Natl. Acad. Sci. USA.

[153] Sharma E., Kapoor R. (2017). Insights into the molecular interplay of virulence factors in *Botrytis cinerea*. Australas. Plant Pathol..

[154] Tang J., Sui Z., Li R., Xu Y., Xiang L., Fu S., Wei J., Cai X., Wu M., Zhang J. (2023). The Gβ-like protein Bcgbl1 regulates development and pathogenicity of the gray mold *Botrytis cinerea* via modulating two MAP kinase signaling pathways. PLoS Pathog..

[155] Rui O., Hahn M. (2007). The *Slt2*-type MAP kinase *Bmp3* of *Botrytis cinerea* is required for normal saprotrophic growth, conidiation, plant surface sensing and host tissue colonization. Mol. Plant Pathol..

[156] Hohmann S. (2002). Osmotic stress signaling and osmoadaptation in yeasts. Microbiol. Mol. Biol. Rev..

[157] Segmüller N., Ellendorf U., Tudzynski B., Tudzynski P. (2007). BcSAK1, a stress-activated mitogen-activated protein kinase, is involved in vegetative differentiation and pathogenicity in *Botrytis cinerea*. Eukaryot. Cell.

[158] Dixon K.P., Xu J.R., Smirnoff N., Talbot N.J. (1999). Independent signaling pathways regulate cellular turgor during hyperosmotic stress and appressorium-mediated plant infection by *Magnaporthe grisea*. Plant Cell.

[159] Kojima K., Takano Y., Yoshimi A., Tanaka C., Kikuchi T., Okuno T. (2004). Fungicide activity through activation of a fungal signalling pathway. Mol. Microbiol..

[160] Park S.M., Choi E.S., Kim M.J., Cha B.J., Yang M.S., Kim D.H. (2004). Characterization of HOG1 homologue, CpMK1, from *Cryphonectria parasitica* and evidence for hypovirus-mediated perturbation of its phosphorylation in response to hypertonic stress. Mol. Microbiol..

[161] Liu W., Leroux P., Fillinger S. (2008). The HOG1-like MAP kinase *Sak1* of *Botrytis cinerea* is negatively regulated by the upstream histidine kinase *Bos1* and is not involved in dicarboximide-and phenylpyrrole-resistance. Fungal Genet. Biol..

[162] Bahn Y.S. (2008). Master and commander in fungal pathogens: The two-component system and the *HOG* signaling pathway. Eukaryot. Cell.

[163] Viaud M., Fillinger S., Liu W., Polepalli J.S., Le Pêcheur P., Kunduru A.R., Leroux P., Legendre L. (2006). A class III histidine kinase acts as a novel virulence factor in *Botrytis cinerea*. Mol. Plant Microbe Interact..

[164] Liu W., Soulié M.C., Perrino C., Fillinger S. (2011). The osmosensing signal transduction pathway from *Botrytis cinerea* regulates cell wall integrity and MAP kinase pathways control melanin biosynthesis with influence of light. Fungal Genet. Biol..

[165] Dodds P.N., Rathjen J.P. (2010). Plant immunity: Towards an integrated view of plant–pathogen interactions. Nat. Rev. Genet..

[166] Zhou J.-M., Zhang Y. (2020). Plant immunity: Danger perception and signaling. Cell.

[167] Yuan M., Ngou B.P.M., Ding P., Xin X.-F. (2021). PTI-ETI crosstalk: An integrative view of plant immunity. Curr. Opin. Plant Biol..

[168] Ngou B.P.M., Jones J.D.G., Ding P. (2022). Plant immune networks. Trends Plant Sci..

[169] Mengiste T. (2012). Plant immunity to necrotrophs. Ann. Rev. Phytopathol..

[170] Wang W., Wang Z.-Y. (2014). At the intersection of plant growth and immunity. Cell Host Microbe.

[171] Ghozlan M.H., El-Argawy E., Tokgöz S., Lakshman D.K., Mitra A. (2020). Plant defense against necrotrophic pathogens. Am. J. Plant Sci..

[172] Rowe H.C., Walley J.W., Corwin J., Chan E.K., Dehesh K., Kliebenstein D.J. (2010). Deficiencies in jasmonate-mediated plant defense reveal quantitative variation in *Botrytis cinerea* pathogenesis. PLoS Pathog..

[173] Grant M.R., Jones D.G. (2009). Hormone (dis)harmony moulds plant health and disease. Science.

[174] Robuschi L., Mariani O., Perk E.A., Cerrudo I., Villareal F., Laxalt A.M. (2024). *Arabidopsis thaliana* phosphoinositide-specific phospholipase C 2 is required for *Botrytis cinerea* proliferation. Plant Sci..

[175] El Oirdi M., El Rahman T.A., Rigano L., El Hadrami A., Rodriguez M.C., Daayf F., Vojnov A., Bouarab K. (2011). *Botrytis cinerea* manipulates the antagonistic effects between immune pathways to promote disease development in tomato. Plant Cell.

[176] Zhang M., Li W., Zhang T., Liu Y., Liu L. (2024). *Botrytis cinerea*-induced F-box protein 1 enhances disease resistance by inhibiting JAO/JOX-mediated jasmonic acid catabolism in *Arabidopsis*. Mol. Plant.

[177] Liu X., Cao X., Chen M., Li D., Zhang Z. (2024). Two transcription factors RhERF005/RhCCCH12 regulate rose resistance to *Botrytis cinerea* by modulating cytokinin levels. J. Exp. Bot..

[178] Zhang H., Hu Z., Lei C., Zheng C., Wang J., Shao S., Li X., Xia X., Cai X., Zhou J. (2018). A plant phytosulfokine peptide initiates auxin-dependent immunity through cytosolic Ca^2+^ signaling in tomato. Plant Cell.

[179] Llorente F., Muskett P., Sánchez-Vallet A., López G., Ramos B., Sánchez-Rodríguez C., Jordá L., Parker J., Molina A. (2008). Repression of the auxin response pathway increases *Arabidopsis* susceptibility to necrotrophic fungi. Mol. Plant.

[180] Sharon A., Elad Y., Barakat R., Tudzynski P., Elad Y., Williamson P., Tudznski P., Delen N. (2004). Phytohormones in *Botrytis*-plant interactions. Botrytis: Biology, Pathology and Control.

[181] Li Z.-X., Yang S., Wang X., Liao W.-H., Zhang W.-L., Liu J., Liu G.-H., Tang J.-M. (2023). Widely targeted metabolomics analysis reveals the effect of exogenous auxin on postharvest resistance to *Botrytis cinerea* in kiwifruit (*Actinidia chinensis* L.). Postharvest Biol..

[182] Moffat C.S., Ingle R.A., Wathugala D.L., Saunders N.J., Knight H., Knight M.R. (2012). ERF5 and ERF6 play redundant roles as positive regulators of JA/Et-mediated defense against *Botrytis cinerea* in Arabidopsis. PLoS ONE.

[183] Deng H., Pei Y., Xu X., Du X., Xue Q., Gao Z., Shu P., Wu Y., Liu Z., Jian Y. (2024). Ethylene-MPK8-ERF.C1-PR module confers resistance against *Botrytis cinerea* in tomato fruit without compromising ripening. New Phytol..

[184] Ono E., Mise K., Takano Y. (2020). RLP23 is required for *Arabidopsis* immunity against the grey mould pathogen *Botrytis cinerea*. Sci. Rep..

[185] Denancé N., Sánchez-Vallet A., Goffner D., Molina A. (2013). Disease resistance or growth: The role of plant hormones in balancing immune responses and fitness costs. Front. Plant Sci..

[186] Liu N., Xu Y., Li Q., Cao Y., Yang D., Liu S., Wang X., Mi Y., Liu Y., Ding C. (2022). A lncRNA fine-tunes salicylic acid biosynthesis to balance plant immunity and growth. Cell Host Microbe.

[187] Mao G., Meng X., Liu Y., Zheng Z., Chen Z., Zhang S. (2011). Phosphorylation of a WRKY transcription factor by two pathogen-responsive MAPKs drives phytoalexin biosynthesis in *Arabidopsis*. Plant Cell.

[188] Birkenbihl R.P., Diezel C., Somssich I.E. (2012). Arabidopsis WRKY33 is a key transcriptional regulator of hormonal and metabolic responses toward *Botrytis cinerea* infection. Plant Physiol..

[189] Ding L., Wu Z., Xiang J., Cao X., Xu S., Zhang Y., Zhang D., Teng N. (2024). A LlWRKY33-LlHSFA4-LlCAT2 module confers resistance to *Botrytis cinerea* in lily. Hort. Res..

[190] Liu S., Kracher B., Ziegler J., Birkenbihl R.P., Somssich I.E. (2015). Negative regulation of ABA signaling by WRKY33 is critical for *Arabidopsis* immunity towards *Botrytis cinerea* 2100. eLife.

[191] Hael-Conrad H., Abou-Mansour E., Díaz-Ricci J.-C., Métraux J.-P., Serrano M. (2015). The novel elicitor AsES triggers a defense response against *Botrytis cinerea* in *Arabidopsis thaliana*. Plant Sci..

[192] Yang Q., Yang J., Wang Y., Du J., Zhang J., Luisi B.F., Liang W. (2022). Broad-spectrum chemicals block ROS detoxification to prevent plant fungal invasion. Curr. Biol..

[193] He J., Kong M., Qian Y., Gong M., Lv G., Song J. (2023). Cellobiose elicits immunity in lettuce conferring resistance to *Botrytis cinerea*. J. Exp. Bot..

[194] Felemban A., Moreno J.C., Mi J., Ali S., Sham A., Abuqamar S.F., Al-Babili S. (2024). The apocarotenoid β-ionone regulates the transcriptome of *Arabidopsis thaliana* and increases its resistance against *Botrytis cinerea*. Plant J..

[195] De Vega D., Holden N., Hedley P.E., Morris J., Luna E., Newton A. (2021). Chitosan primes plant defence mechanisms against *Botrytis cinerea*, including expression of Avr9/Cf-9 rapidly elicited genes. Plant Cell Environ..

[196] Banani H., Olivieri L., Santoro K., Garibaldi A., Gullino M.L., Spadaro D. (2018). Thyme and savory essential oil efficacy and induction of resistance against *Botrytis cinerea* through priming of defense responses in apple. Foods.

[197] Hou H., Zhang X., Zhao T., Zhou L. (2020). Effects of *Origanum vulgare* essential oil and its two main components, carvacrol and thymol, on the plant pathogen *Botrytis cinerea*. PeerJ..

[198] Li S., Yu Y., Xie P., Zhu X., Yang C., Wang L., Zhang S. (2024). Antifungal activities of l-methionine and l-arginine treatment in vitro and in vivo against *Botrytis cinerea*. Microorganisms.

[199] Iqbal M., Jutzeler M., Franca S.C., Wackers F., Andreasson E., Stenberg J.A. (2022). Bee-vectored *Aureobasidium pullulans* for biological control of gray mold in strawberry. Phytopathology.

[200] Poveda J., Barquero M., González-Andrés F. (2020). Insight into the microbiological control strategies against *Botrytis cinerea* using systemic plant resistance activation. Agronomy.

[201] Roca-Couso R., Flores-Félix J.D., Rivas R. (2021). Mechanisms of action of microbial biocontrol agents against *Botrytis cinerea*. J. Fungi.

[202] Meng F., Ly R., Cheng M., Mo F., Zhang N., Qi H., Liu J., Chen X., Liu Y., Ghanizadeh H. (2022). Insights into the molecular basis of biocontrol of *Botrytis cinerea* by *Clonostachys rosea* in tomato. Sci. Hortic..

[203] Altieri V., Rossi V., Fedele G. (2023). Efficacy of preharvest application of biocontrol agents against gray mold in grapevine. Front. Plant Sci..

[204] Zheng L., Han Z., Wang S., Gao A., Liu L., Pan H., Zhang H. (2024). Transcriptomic analysis and knockout experiments reveal the role of *suhB* in the biocontrol effects of *Pantoea jilinensis* D25 on *Botrytis cinerea*. Sci. Total Environ..

[205] Zhao Y., Wang Z.-J., Wang C.-B., Tan B.-Y., Luo X.-D. (2023). New and antifungal diterpenoids of sunflower against gray mold. J. Agric. Food Chem..

[206] Chen D., Zhang Z., Chen Y., Li B., Chen T., Tian S. (2024). Transcriptional landscape of pathogen-responsive lncRNAs in tomato unveils the role of hydrolase encoding genes in response to *Botrytis cinerea* invasion. Plant Cell Environ..

[207] Wu F., Huang Y., Jiang W., Jin W. (2023). Genome-wide identification and validation of tomato encoded sRNA as the cross species antifungal factors targeting the virulence genes of *Botrytis cinerea*. Front. Plant Sci..

[208] Geley S., Müller C. (2004). RNAi: Ancient mechanism with a promising future. Exp. Gerontol..

[209] Muhammad T., Zhang F., Zhang Y., Liang Y. (2019). RNA interference: A natural immune system of plants to counteract biotic stressors. Cells.

[210] Halder K., Chaudhuri A., Abdin M.Z., Majee M., Datta A. (2022). RNA interference for improving disease resistance in plants and its relevance in this clustered regularly interspaced short palindromic repeats-dominated era in terms of dsRNA-based biopesticides. Front. Plant Sci..

[211] Fire A., Xu S., Montgomery M.K., Kostas S.A., Driver S.E., Mello C.C. (1998). Potent and specific genetic interference by double-stranded RNA in *Caenorhabditis elegans*. Nature.

[212] Sanford J.C., Johnston S.A. (1985). The concept of parasite-derived resistance—Deriving resistance genes from the parasite’s own genome. J. Theor. Biol..

[213] Guo S., Kemphues K.J. (1995). *par-1*, a gene required for establishing polarity in *C. elegans* embryos, encodes a putative Ser/Thr kinase that is asymmetrically distributed. Cell.

[214] Moerman D., Baillie D. (1979). Genetic organization in *Caenorhabditis elegans*: Fine structure analysis of the *unc-22* gene. Genetics.

[215] Napoli C., Lemieux C., Jorgensen R. (1990). Introduction of a chimeric chalcone synthase gene into petunia results in reversible co-suppression of homologous genes *in trans*. Plant Cell.

[216] Romano N., Macino G. (1992). Quelling: Transient inactivation of gene expression in *Neurospora crassa* by transformation with homologous sequences. Mol. Microbiol..

[217] Jorgensen R.A. (1995). Cosuppression, flower color patterns, and metastable gene expression states. Science.

[218] Pal-Bhadra M., Bhadra U., Birchler J.A. (1997). Cosuppression in *Drosophila*: Gene silencing of *Alcohol dehydrogenase* by *white-Adh* transgenes is *Polycomb* dependent. Cell.

[219] Angell S.M., Baulcombe D.C. (1997). Consistent gene silencing in transgenic plants expressing a replicating *potato virus X* RNA. EMBO J..

[220] Dougherty W.G., Lindbo J.A., Smith H.A., Parks T.D., Swaney S., Proebsting W.M. (1994). RNA-mediated virus resistance in transgenic plants: Exploitation of a cellular pathway possibly involved in RNA degradation. Mol. Plant Microbe Interact..

[221] Kumagai M.H., Donson J., Della-Cioppa G., Harvey D., Hanley K., Grill L. (1995). Cytoplasmic inhibition of carotenoid biosynthesis with virus-derived RNA. Proc. Natl. Acad. Sci. USA.

[222] Lindbo J.A., Silva-Rosales L., Proebsting W.M., Dougherty W.G. (1993). Induction of a highly specific antiviral state in transgenic plants: Implications for regulation of gene expression and virus resistance. Plant Cell.

[223] Ruiz M.T., Voinnet O., Baulcombe D.C. (1998). Initiation and maintenance of virus-induced gene silencing. Plant Cell.

[224] Lu P.Y., Xie F.Y., Woodle M.C. (2003). siRNA-mediated antitumorgenesis for drug target validation and therapeutics. Curr. Opin. Mol. Ther..

[225] Lu P.Y., Xie F.Y., Woodle M.C. (2005). Modulation of angiogenesis with siRNA inhibitors for novel therapeutics. Trends Mol. Med..

[226] Aravin A.A., Naumova N.M., Tulin A.V., Vagin V.V., Rozovsky Y.M., Gvozdev V.A. (2001). Double-stranded RNA-mediated silencing of genomic tandem repeats and transposable elements in the *D. melanogaster* germline. Curr. Biol..

[227] Catalanotto C., Azzalin G., Macino G., Cogoni C. (2000). Gene silencing in worms and fungi. Nature.

[228] Fagard M., Boutet S., Morel J.B., Bellini C., Vaucheret H. (2000). AGO1, QDE-2, and RDE-1 are related proteins required for post-transcriptional gene silencing in plants, quelling in fungi, and RNA interference in animals. Proc. Natl. Acad. Sci. USA.

[229] Schmidt A., Palumbo G., Bozzetti M.P., Tritto P., Pimpinelli S., Schäfer U. (1999). Genetic and molecular characterization of *sting*, a gene involved in crystal formation and meiotic drive in the male germ line of *Drosophila melanogaster*. Genetics.

[230] De Carvalho F., Gheysen G., Kushnir S., Van Montagu M., Inze D., Castresana C. (1992). Suppression of *β*-1,3-glucanase transgene expression in homozygous plants. EMBO J..

[231] Mello C.C., Conte D. (2004). Revealing the world of RNA interference. Nature.

[232] Hamilton A.J., Baulcombe D.C. (1999). A species of small antisense RNA in posttranscriptional gene silencing in plants. Science.

[233] Hammond S.M., Bernstein E., Beach D., Hannon G.J. (2000). An RNA-directed nuclease mediates post-transcriptional gene silencing in *Drosophila* cells. Nature.

[234] Parrish S., Fleenor J., Xu S., Mello C., Fire A. (2000). Functional anatomy of a dsRNA trigger: Differential requirement for the two trigger strands in RNA interference. Mol. Cell.

[235] Parrish S., Fire A. (2001). Distinct roles for RDE-1 and RDE-4 during RNA interference in *Caenorhabditis elegans*. RNA.

[236] Zamore P.D., Tuschl T., Sharp P.A., Bartel D.P. (2000). RNAi: Double-stranded RNA directs the ATP-dependent cleavage of mRNA at 21 to 23 nucleotide intervals. Cell.

[237] Ketting R.F., Haverkamp T.H., van Luenen H.G., Plasterk R.H. (1999). *Mut-7* of *C. elegans*, required for transposon silencing and RNA interference, is a homolog of Werner syndrome helicase and RNaseD. Cell.

[238] Ito H. (2012). Small RNAs and transposon silencing in plants. Dev. Growth Differ..

[239] Cornec A., Poirier E.Z. (2023). Interplay between RNA interference and transposable elements in mammals. Front. Immunol..

[240] Wallis D.C., Nguyen D.A.H., Uebel C.J., Phillips C.M. (2019). Visualization and quantification of transposon activity in *Caenorhabditis elegans* RNAi pathway mutants. G3-Genes Genom. Genet..

[241] Anandalakshmi R., Pruss G.J., Ge X., Marathe R., Mallory A.C., Smith T.H., Vance V.B. (1998). A viral suppressor of gene silencing in plants. Proc. Natl. Acad. Sci. USA.

[242] Akbar S., Wei Y., Zhang  M.Q. (2022). RNA interference: Promising approach to combat plant viruses. Int. J. Mol. Sci..

[243] Mourrain P., Béclin C., Elmayan T., Feuerbach F., Godon C., Morel J.B., Jouette D., Lacombe A.-M., Nikic S., Picault N. (2000). *Arabidopsis SGS2* and *SGS3* genes are required for posttranscriptional gene silencing and natural virus resistance. Cell.

[244] Reed J.C., Kasschau K.D., Prokhnevsky A.I., Gopinath K., Pogue G.P., Carrington J.C., Dolja V.V. (2023). Suppressor of RNA silencing encoded by *Beet yellows virus*. Virology.

[245] Vance V., Vaucheret H. (2001). RNA silencing in plants–defense and counterdefense. Science.

[246] Voinnet O. (2001). RNA silencing as a plant immune system against viruses. Trends Genet..

[247] Kang H., Ga Y.J., Kim S.H., Cho Y.H., Kim J.W., Kim C., Yeh J.Y. (2023). Small interfering RNA (siRNA)-based therapeutic applications against viruses: Principles, potential, and challenges. J. Biomed. Sci..

[248] Stevenson D.S., Jarvis P. (2003). Chromatin silencing: RNA in the driving seat. Curr. Biol..

[249] Bernard P., Maure J.F., Partridge J.F., Genier S., Javerzat J.P., Allshire R.C. (2001). Requirement of heterochromatin for cohesion at centromeres. Science.

[250] Bernard P., Allshire R.C. (2002). Centromeres become unstuck without heterochromatin. Trends Cell Biol..

[251] Volpe T., Schramke V., Hamilton G.L., White S.A., Teng G., Martienssen R.A., Allshire R.C. (2003). RNA interference is required for normal centromere function in fission yeast. Chromosome Res..

[252] Bhattacharjee S., Roche B., Martienssen R.A. (2019). RNA-induced initiation of transcriptional silencing (RITS) complex structure and function. RNA Biol..

[253] Martienssen R., Moazed D. (2015). RNAi and heterochromatin assembly. Cold Spring Harb. Perspect. Biol..

[254] Chalamcharla V.R., Folco H.D., Dhakshnamoorthy J., Grewal S.I. (2015). Conserved factor Dhp1/Rat1/Xrn2 triggers premature transcription and nucleates heterochromatin to promote gene silencing. Proc. Natl. Acad. Sci. USA.

[255] Hynes M.J., Todd R.B. (2003). Detection of unpaired DNA at meiosis results in RNA-mediated silencing. Bioessays.

[256] Wu J., Yang J., Cho W.C., Zheng Y. (2020). Argonaute proteins: Structural features, functions and emerging roles. J. Adv. Res..

[257] Grishok A., Pasquinelli A.E., Conte D., Li N., Parrish S., Ha I., Baillie D.L., Fire A., Ruvkun G., Mello C.C. (2001). Genes and mechanisms related to RNA interference regulate expression of the small temporal RNAs that control *C. elegans* developmental timing. Cell.

[258] Brennecke J., Hipfner D.R., Stark A., Russell R.B., Cohen S.M. (2003). *bantam* encodes a developmentally regulated microRNA that controls cell proliferation and regulates the proapoptotic gene *hid* in *Drosophila*. Cell.

[259] Svoboda P. (2020). Key mechanistic principles and considerations concerning RNA interference. Front. Plant Sci..

[260] Lata H., Sharma A., Chadha S., Kaur M., Kumar P. (2022). RNA interference (RNAi) mechanism and application in vegetable crops. J. Hort. Sci. Biotech..

[261] Carthew R.W. (2001). Gene silencing by double-stranded RNA. Curr. Opin. Cell Biol..

[262] Cogoni C., Macino G. (2000). Post-transcriptional gene silencing across kingdoms. Curr. Opin. Genet. Dev..

[263] Tuschl T. (2001). RNA interference and small interfering RNAs. ChemBioChem..

[264] Torri A., Jaeger J., Pradeu T., Saleh M.-C. (2022). The origin of RNA interference: Adaptive or neutral evolution?. PLoS Biol..

[265] Ipsaro J.J., Joshua-Tor L. (2015). From guide to target: Molecular insights into eukaryotic RNA-interference machinery. Nat. Struct. Mol. Biol..

[266] Matzke M., Matzke A.J., Kooter J.M. (2001). RNA: Guiding gene silencing. Science.

[267] Marker S., Le Mouël A., Meyer E., Simon M. (2010). Distinct RNA-dependent RNA polymerases are required for RNAi triggered by double-stranded RNA versus truncated transgenes in *Paramecium tetraurelia*. Nucleic Acids Res..

[268] Filippov V., Solovyev V., Filippova M., Gill S.S. (2000). A novel type of RNase III family proteins in eukaryotes. Gene.

[269] Bernstein E., Caudy A.A., Hammond S.M., Hannon G.J. (2001). Role for a bidentate ribonuclease in the initiation step of RNA interference. Nature.

[270] Plasterk R.H. (2002). RNA silencing: The genome’s immune system. Science.

[271] Limera C., Sabbadini S., Sweet J.B., Mezzetti B. (2017). New biotechnological tools for the genetic improvement of major woody fruit species. Front. Plant Sci..

[272] Lee Y., Jeon K., Lee J.T., Kim S., Kim V.N. (2002). MicroRNA maturation: Stepwise processing and subcellular localization. EMBO J..

[273] Lee Y., Ahn C., Han J., Choi H., Kim J., Yim J., Lee J., Provost P., Rådmark O., Kim S. (2003). The nuclear RNase III Drosha initiates microRNA processing. Nature.

[274] Zeng Y. (2006). Principles of micro-RNA production and maturation. Oncogene.

[275] Basyuk E., Suavet F., Doglio A., Bordonné R., Bertrand E. (2003). Human let-7 stem–loop precursors harbor features of RNase III cleavage products. Nucleic Acids Res..

[276] Bohnsack M.T., Czaplinski K., Görlich D. (2004). Exportin 5 is a RanGTP-dependent dsRNA-binding protein that mediates nuclear export of pre-miRNAs. RNA.

[277] Lund E., Guttinger S., Calado A., Dahlberg J.E., Kutay U. (2004). Nuclear export of microRNA precursors. Science.

[278] Yi R., Qin Y., Macara I.G., Cullen B.R. (2003). Exportin-5 mediates the nuclear export of pre-microRNAs and short hairpin RNAs. Genes Dev..

[279] Carrington J.C., Ambros V. (2003). Role of microRNAs in plant and animal development. Science.

[280] Dong Q., Hu B., Zhang C. (2022). microRNAs and their roles in plant development. Front. Plant Sci..

[281] Catalanotto C., Pallotta M., ReFalo P., Sachs M.S., Vayssie L., Macino G., Cogoni C. (2004). Redundancy of the two dicer genes in transgene-induced posttranscriptional gene silencing in *Neurospora crassa*. Mol. Cell. Biol..

[282] Ketting R.F., Fischer S.E., Bernstein E., Sijen T., Hannon G.J., Plasterk R.H. (2001). Dicer functions in RNA interference and in synthesis of small RNA involved in developmental timing in *C. elegans*. Genes Dev..

[283] Golden T.A., Schauer S.E., Lang J.D., Pien S., Mushegian A.R., Grossniklaus U., Meinke D.W., Ray A. (2002). *SHORT INTEGUMENTS1/suspensor1/Carpel Factory*, a Dicer homolog, is a maternal effect gene required for embryo development in *Arabidopsis*. Plant Physiol..

[284] Knight S.W., Bass B.L. (2011). A role for the RNase III enzyme DCR-1 in RNA interference and germ line development in *Caenorhabditis elegans*. Science.

[285] Park W., Li J., Song R., Messing J., Chen X. (2002). CARPEL FACTORY, a Dicer homolog, and HEN1, a novel protein, act in microRNA metabolism in *Arabidopsis thaliana*. Curr. Biol..

[286] Provost P., Dishart D., Doucet J., Frendewey D., Samuelsson B., Rådmark O. (2002). Ribonuclease activity and RNA binding of recombinant human Dicer. EMBO J..

[287] Zhang H., Kolb F.A., Brondani V., Billy E., Filipowicz W. (2002). Human Dicer preferentially cleaves dsRNAs at their termini without a requirement for ATP. EMBO J..

[288] Paturi S., Deshmukh M.V. (2021). A glimpse of “Dicer Biology” through the structural and functional perspective. Front. Mol. Biosci..

[289] Zhang H., Kolb F.A., Jaskiewicz L., Westhof E., Filipowicz W. (2004). Single processing center models for human Dicer and bacterial RNase III. Cell.

[290] MacRae I.J., Doudna J.A. (2007). Ribonuclease revisited: Structural insights into ribonuclease III family enzymes. Curr. Opin. Struct. Biol..

[291] Murzin A.G. (1993). OB (oligonucleotide/oligosaccharide binding)-fold: Common structural and functional solution for non-homologous sequences. EMBO J..

[292] Ma J.B., Ye K., Patel D.J. (2004). Structural basis for overhang-specific small interfering RNA recognition by the PAZ domain. Nature.

[293] Vermeulen A., Behlen L., Reynolds A., Wolfson A., Marshall W.S., Karpilow J.O.N., Khvorova A. (2005). The contributions of dsRNA structure to Dicer specificity and efficiency. RNA.

[294] Tian Y., Simanshu D.K., Ma J.B., Park J.E., Heo I., Kim V.N., Patel D.J. (2014). A phosphate-binding pocket within the platform-PAZ-connector helix cassette of human Dicer. Mol. Cell.

[295] MacRae I.J., Zhou K., Li F., Repic A., Brooks A.N., Cande W.Z., Adams P.D., Doudna J.A. (2006). Structural basis for double-stranded RNA processing by Dicer. Science.

[296] Lau P.W., Guiley K.Z., De N., Potter C.S., Carragher B., MacRae I.J. (2012). The molecular architecture of human Dicer. Nat. Struct. Mol. Biol..

[297] Billy E., Brondani V., Zhang H., Müller U., Filipowicz W. (2001). Specific interference with gene expression induced by long, double-stranded RNA in mouse embryonal teratocarcinoma cell lines. Proc. Natl. Acad. Sci. USA.

[298] Nykänen A., Haley B., Zamore P.D. (2001). ATP requirements and small interfering RNA structure in the RNA interference pathway. Cell.

[299] Martinez J., Patkaniowska A., Urlaub H., Lührmann R., Tuschl T. (2002). Single-stranded antisense siRNAs guide target RNA cleavage in RNAi. Cell.

[300] Schwarz D.S., Hutvágner G., Haley B., Zamore P.D. (2002). Evidence that siRNAs function as guides, not primers, in the *Drosophila* and human RNAi pathways. Mol. Cell.

[301] Winter J., Diederichs S. (2011). Argonaute proteins regulate microRNA stability: Increased microRNA abundance by Argonaute proteins is due to microRNA stabilization. RNA Biol..

[302] Elbashir S.M., Lendeckel W., Tuschl T. (2001). RNA interference is mediated by 21- and 22-nucleotide RNAs. Genes Dev..

[303] Song J.J., Smith S.K., Hannon G.J., Joshua-Tor L. (2004). Crystal structure of Argonaute and its implications for RISC slicer activity. Science.

[304] Iwakawa H.O., Tomari Y. (2022). Life of RISC: Formation, action, and degradation of RNA-induced silencing complex. Mol. Cell.

[305] Rosa C., Kuo Y.W., Wuriyanghan H., Falk B.W. (2018). RNA interference mechanisms and applications in plant pathology. Annu. Rev. Phytopathol..

[306] Frank F., Hauver J., Sonenberg N., Nagar B. (2012). *Arabidopsis* Argonaute MID domains use their nucleotide specificity loop to sort small RNAs. EMBO J..

[307] Lingel A., Simon B., Izaurralde E., Sattler M. (2003). Structure and nucleic-acid binding of the *Drosophila* Argonaute 2 PAZ domain. Nature.

[308] Lingel A., Simon B., Izaurralde E., Sattler M. (2004). Nucleic acid 3′-end recognition by the Argonaute2 PAZ domain. Nat. Struct. Mol. Biol..

[309] Tolia N.H., Joshua-Tor L. (2007). Slicer and the argonautes. Nat. Chem. Biol..

[310] Kuhn C.D., Joshua-Tor L. (2013). Eukaryotic Argonautes come into focus. Trends Biochem. Sci..

[311] Sijen T., Fleenor J., Simmer F., Thijssen K.L., Parrish S., Timmons L., Plasterk R.H.A., Fire A. (2001). On the role of RNA amplification in dsRNA-triggered gene silencing. Cell.

[312] Cogoni C., Macino G. (1999). Gene silencing in *Neurospora crassa* requires a protein homologous to RNA-dependent RNA polymerase. Nature.

[313] Dalmay T., Hamilton A., Rudd S., Angell S., Baulcombe D.C. (2000). An RNA-dependent RNA polymerase gene in *Arabidopsis* is required for posttranscriptional gene silencing mediated by a transgene but not by a virus. Cell.

[314] Smardon A., Spoerke J.M., Stacey S.C., Klein M.E., Mackin N., Maine E.M. (2000). EGO-1 is related to RNA-directed RNA polymerase and functions in germ-line development and RNA interference in *C. elegans*. Curr. Biol..

[315] Mlotshwa S., Voinnet O., Mette M.F., Matzke M., Vaucheret H., Ding S.W., Pruss G., Vance V.B. (2002). RNA silencing and the mobile silencing signal. Plant Cell.

[316] Zilberman D., Cao X., Jacobsen S.E. (2003). *ARGONAUTE4* control of locus-specific siRNA accumulation and DNA and histone methylation. Science.

[317] Xie Z., Johansen L.K., Gustafson A.M., Kasschau K.D., Lellis A.D., Zilberman D., Jacobsen S.E., Carrington J.C. (2004). Genetic and functional diversification of small RNA pathways in plants. PLoS Biol..

[318] Martienssen R.A., Zaratiegui M., Goto D.B. (2005). RNA interference and heterochromatin in the fission yeast *Schizosaccharomyces pombe*. Trends Genet..

[319] Catalanotto C., Azzalin G., Macino G., Cogoni C. (2002). Involvement of small RNAs and role of the *qde* genes in the gene silencing pathway in *Neurospora*. Genes Dev..

[320] Cogoni C., Macino G. (1999). Posttranscriptional gene silencing in *Neurospora* by a RecQ DNA helicase. Science.

[321] Nakayashiki H. (2005). RNA silencing in fungi: Mechanisms and applications. FEBS Lett..

[322] Hammond T.M. (2017). Sixteen years of meiotic silencing by unpaired DNA. Adv. Genet..

[323] Son H., Min K., Lee J., Raju N.B., Lee Y.W. (2011). Meiotic silencing in the homothallic fungus *Gibberella zeae*. Fungal Biol..

[324] Decker L.M., Boone E.C., Xiao H., Shanker B.S., Boone S.F., Kingston S.L., Lee S.A., Hammond T.M., Shiu P.K.T. (2015). Complex formation of RNA silencing proteins in the perinuclear region of *Neurospora crassa*. Genetics.

[325] Sun Q., Choi G.H., Nuss D.L. (2009). A single Argonaute gene is required for induction of RNA silencing antiviral defense and promotes viral RNA recombination. Proc. Natl. Acad. Sci. USA.

[326] Segers G.C., Van Wezel R., Zhang X., Hong Y., Nuss D.L. (2006). Hypovirus papain-like protease p29 suppresses RNA silencing in the natural fungal host and in a heterologous plant system. Eukaryot. Cell.

[327] Kotta-Loizou I., Coutts R.H. (2017). Mycoviruses in *Aspergilli*: A comprehensive review. Front. Microbiol..

[328] Hammond T.M., Andrewski M.D., Roossinck M.J., Keller N.P. (2008). *Aspergillus* mycoviruses are targets and suppressors of RNA silencing. Eukaryot. Cell.

[329] Bourc’his D., Voinnet O. (2010). A small-RNA perspective on gametogenesis, fertilization, and early zygotic development. Science.

[330] Sijen T., Plasterk R.H.A. (2003). Transposon silencing in the *Caenorhabditis elegans* germ line by natural RNAi. Nature.

[331] Nolan T., Braccini L., Azzalin G., De Toni A., Macino G., Cogoni C. (2005). The post-transcriptional gene silencing machinery functions independently of DNA methylation to repress a LINE1-like retrotransposon in *Neurospora crassa*. Nucleic Acids Res..

[332] Yamanaka S., Mehta S., Reyes-Turcu F.E., Zhuang F., Fuchs R.T., Rong Y., Robb G.B., Grewal S.I. (2013). RNAi triggered by specialized machinery silences developmental genes and retrotransposons. Nature.

[333] Nunes C.C., Gowda M., Sailsbery J., Xue M., Chen F., Brown D.E., Oh Y.Y., Mitchell T.K., Dean R.A. (2011). Diverse and tissue-enriched small RNAs in the plant pathogenic fungus, *Magnaporthe oryzae*. BMC Genom..

[334] Navarro-Mendoza M.I., Pérez-Arques C., Panchal S., Nicolás F.E., Mondo S.J., Ganguly P., Pangilinan J., Grigoriev I.V., Heitman J., Sanyal K. (2019). Early diverging fungus *Mucor circinelloides* lacks centromeric histone CENP-A and displays a mosaic of point and regional centromeres. Curr. Biol..

[335] Pérez-Arques C., Navarro-Mendoza M.I., Murcia L., Navarro E., Garre V., Nicolás F.E. (2020). A non-canonical RNAi pathway controls virulence and genome stability in *Mucorales*. PLoS Genet..

[336] Sperschneider J., Jones A.W., Nasim J., Xu B., Jacques S., Upadhyaya N.M., Mago R., Figueroa M., Singh K.B., Stone E.A. (2021). The stem rust fungus *Puccinia graminis* f. sp.. tritici induces centromeric small RNAs during late infection that direct genome-wide DNA methylation. BMC Biol..

[337] Wang X., Hsueh Y.P., Li W., Floyd A., Skalsky R., Heitman J. (2010). Sex-induced silencing defends the genome of *Cryptococcus neoformans* via RNAi. Genes Dev..

[338] Burke J.E., Longhurst A.D., Natarajan P., Rao B., Liu J., Sales-Lee J., Mortensen Y., Moresco J.J., Diedrich J.K., Yates J.R. (2019). A non-dicer RNase III and four other novel factors required for RNAi-mediated transposon suppression in the human pathogenic yeast *Cryptococcus neoformans*. G3-Genes Genom. Genet..

[339] Jiang N., Yang Y., Janbon G., Pan J., Zhu X. (2012). Identification and functional demonstration of miRNAs in the fungus *Cryptococcus neoformans*. PLoS ONE.

[340] Lax C., Pérez-Arques C., Navarro-Mendoza M.I., Cánovas-Márquez J.T., Tahiri G., Pérez-Ruiz J.A., Osorio-Concepción M., Murcia-Flores L., Navarro E., Garre V. (2020). Genes, pathways, and mechanisms involved in the virulence of *mucorales*. Genes.

[341] Torres-Martínez S., Ruiz-Vázquez R.M. (2017). The RNAi universe in fungi: A varied landscape of small RNAs and biological functions. Annu. Rev. Microbiol..

[342] Wagner L., De Hoog S., Alastruey-Izquierdo A., Voigt K., Kurzai O., Walther G. (2019). A revised species concept for opportunistic *Mucor* species reveals species-specific antifungal susceptibility profiles. Antimicrob. Agents Chemoth..

[343] Nicolás F.E., Moxon S., de Haro J.P., Calo S., Grigoriev I.V., Torres-MartÍnez S., Moulton V., Ruiz-Vázquez R.M., Dalmay T. (2010). Endogenous short RNAs generated by Dicer 2 and RNA-dependent RNA polymerase 1 regulate mRNAs in the basal fungus *Mucor circinelloides*. Nucleic Acids Res..

[344] Nicolás F.E., Vila A., Moxon S., Cascales M.D., Torres-Martínez S., Ruiz-Vázquez R.M., Garre V. (2015). The RNAi machinery controls distinct responses to environmental signals in the basal fungus *Mucor circinelloides*. BMC Genom..

[345] De Haro J.P., Calo S., Cervantes M., Nicolás F.E., Torres-Martinez S., Ruiz-Vázquez R.M. (2009). A single dicer gene is required for efficient gene silencing associated with two classes of small antisense RNAs in *Mucor circinelloides*. Eukaryot. Cell.

[346] Nicolás F.E., De Haro J.P., Torres-Martinez S., Ruiz-Vazquez R.M. (2007). Mutants defective in a *Mucor circinelloides* dicer-like gene are not compromised in siRNA silencing but display developmental defects. Fungal Genet. Biol..

[347] Carreras-Villaseñor N., Esquivel-Naranjo E.U., Villalobos-Escobedo J.M., Abreu-Goodger C., Herrera-Estrella A. (2013). The RNAi machinery regulates growth and development in the filamentous fungus *Trichoderma atroviride*. Mol. Microbiol..

[348] Son H., Park A.R., Lim J.Y., Shin C., Lee Y.W. (2017). Genome-wide exonic small interference RNA-mediated gene silencing regulates sexual reproduction in the homothallic fungus *Fusarium graminearum*. PLoS Genet..

[349] Trieu T.A., Calo S., Nicolás F.E., Vila A., Moxon S., Dalmay T., Torres-Martínez S., Garre V., Ruiz-Vázquez R.M. (2015). A non-canonical RNA silencing pathway promotes mRNA degradation in basal fungi. PLoS Genet..

[350] Cánovas-Márquez J.T., Navarro-Mendoza M.I., Pérez-Arques C., Lax C., Tahiri G., Pérez-Ruiz J.A., Lorenzo-Gutiérrez D., Calo S., López-García S., Navarro E. (2021). Role of the non-canonical RNAi pathway in the antifungal resistance and virulence of mucorales. Genes.

[351] Calo S., Shertz-Wall C., Lee S.C., Bastidas R.J., Nicolás F.E., Granek J.A., Mieczkowski P., Torres-Martínez S., Ruiz-Vázquez R.M., Cardenas M.E. (2014). Antifungal drug resistance evoked via RNAi-dependent epimutations. Nature.

[352] Chang Z., Billmyre R.B., Lee S.C., Heitman J. (2019). Broad antifungal resistance mediated by RNAi-dependent epimutation in the basal human fungal pathogen *Mucor circinelloides*. PLoS Genet..

[353] Calo S., Nicolás F.E., Lee S.C., Vila A., Cervantes M., Torres-Martinez S., Ruiz-Vazquez R.M., Cardenas M.E., Heitman J. (2017). A non-canonical RNA degradation pathway suppresses RNAi-dependent epimutations in the human fungal pathogen *Mucor circinelloides*. PLoS Genet..

[354] Lee H.C., Li L., Gu W., Xue Z., Crosthwaite S.K., Pertsemlidis A., Lewis Z.A., Freitag M., Selker E.U., Mello C.C. (2010). Diverse pathways generate microRNA-like RNAs and Dicer-independent small interfering RNAs in fungi. Mol. Cell.

[355] Chen Y., Gao Q., Huang M., Liu Y., Liu Z., Liu X., Ma Z. (2015). Characterization of RNA silencing components in the plant pathogenic fungus *Fusarium graminearum*. Sci. Rep..

[356] Zeng W., Wang J., Wang Y., Lin J., Fu Y., Xie J., Jiang D., Chen T., Liu H., Cheng J. (2018). Dicer-like proteins regulate sexual development via the biogenesis of perithecium-specific microRNAs in a plant pathogenic fungus *Fusarium graminearum*. Front. Microbiol..

[357] Hirano Y., Asakawa H., Sakuno T., Haraguchi T., Hiraoka Y. (2020). Nuclear envelope proteins modulating the heterochromatin formation and functions in fission yeast. Cells.

[358] Volpe T.A., Kidner C., Hall I.M., Teng G., Grewal S.I., Martienssen R.A. (2002). Regulation of heterochromatic silencing and histone H3 lysine-9 methylation by RNAi. Science.

[359] Verdel A., Jia S., Gerber S., Sugiyama T., Gygi S., Grewal S.I., Moazed D. (2004). RNAi-mediated targeting of heterochromatin by the RITS complex. Science.

[360] Duempelmann L., Skribbe M., Bühler M. (2020). Small RNAs in the transgenerational inheritance of epigenetic information. Trends Genet..

[361] Hall I.M., Shankaranarayana G.D., Noma K., Ayoub N., Cohen A., Grewal S.I. (2002). Establishment and maintenance of a heterochromatin domain. Science.

[362] Yu R., Wang X., Moazed D. (2018). Epigenetic inheritance mediated by coupling of RNAi and histone H3K9 methylation. Nature.

[363] Bocos-Asenjo I.T., Niño-Sánchez J., Ginésy M., Diez J.J. (2022). New insights on the integrated management of plant diseases by RNA strategies: Mycoviruses and RNA interference. Int. J. Mol. Sci..

[364] Hoang B.T.L., Fletcher S.J., Brosnan C.A., Ghodke A.B., Manzie N., Mitter N. (2022). RNAi as a foliar spray: Efficiency and challenges to field applications. Int. J. Mol. Sci..

[365] Kong X., Yang M., Le B.H., He W., Hou Y. (2022). The master role of siRNAs in plant immunity. Mol. Plant Pathol..

[366] Degnan R.M., McTaggart A.R., Shuey L.S., Pame L.J.S., Smith G.R., Gardiner D.M., Nock V., Soffe R., Sale S., Garrill A. (2023). Exogenous double-stranded RNA inhibits the infection physiology of rust fungi to reduce symptoms in planta. Mol. Plant Pathol..

[367] Wang Q., An B., Hou X., Guo Y., Luo H., He C. (2018). Dicer-like proteins regulate the growth, conidiation, and pathogenicity of *Colletotrichum gloeosporioides* from *Hevea brasiliensis*. Front. Microbiol..

[368] Raman V., Simon S.A., Demirci F., Nakano M., Meyers B.C., Donofrio N.M. (2017). Small RNA functions are required for growth and development of *Magnaporthe oryzae*. Mol. Plant Microbe Interact..

[369] Mochama P., Jadhav P., Neupane A., Lee Marzano S.Y. (2018). Mycoviruses as triggers and targets of RNA silencing in white mold fungus *Sclerotinia sclerotiorum*. Viruses.

[370] Neupane A., Feng C., Mochama P.K., Saleem H., Lee Marzano S.Y. (2019). Roles of argonautes and dicers on *Sclerotinia sclerotiorum* antiviral RNA silencing. Front. Plant Sci..

[371] Feng H., Xu M., Liu Y., Gao X., Yin Z., Voegele R.T., Huang L. (2017). The distinct roles of Argonaute protein 2 in the growth, stress responses and pathogenicity of the apple tree canker pathogen. Forest Pathol..

[372] Jo S.M., Ayukawa Y., Yun S.H., Komatsu K., Arie T. (2018). A putative RNA silencing component protein FoQde-2 is involved in virulence of the tomato wilt fungus *Fusarium oxysporum* f. sp.. lycopersici. J. Gen. Plant Pathol..

[373] Gaffar F.Y., Imani J., Karlovsky P., Koch A., Kogel K.H. (2019). Different components of the RNA interference machinery are required for conidiation, ascosporogenesis, virulence, deoxynivalenol production, and fungal inhibition by exogenous double-stranded RNA in the head blight pathogen *Fusarium graminearum*. Front. Microbiol..

[374] Yin C., Zhu H., Jiang Y., Shan Y., Gong L. (2020). Silencing dicer-like genes reduces virulence and sRNA generation in *Penicillium italicum*, the cause of citrus blue mold. Cells.

[375] Asgari S. (2023). Cross-kingdom RNAi to enhance the efficacy of insect pathogens. Trends Parasitol..

[376] Hua C., Zhao J.H., Guo H.S. (2018). Trans-kingdom RNA silencing in plant-fungal pathogen interactions. Mol. Plant.

[377] Pradhan M., Requena N. (2022). Distinguishing friends from foes: Can smRNAs modulate plant interactions with beneficial and pathogenic organisms?. Curr. Opin. Plant Biol..

[378] Weiberg A., Wang M., Lin F.M., Zhao H., Zhang Z., Kaloshian I., Huang H.D., Jin H. (2013). Fungal small RNAs suppress plant immunity by hijacking host RNA interference pathways. Science.

[379] Ellendorff U., Fradin E.F., de Jonge R., Thomma B.P. (2009). RNA silencing is required for *Arabidopsis* defence against *Verticillium* wilt disease. J. Exp. Bot..

[380] Qiao Y., Liu L., Xiong Q., Flores C., Wong J., Shi J., Wang X., Liu X., Xiang Q., Jiang S. (2013). Oomycete pathogens encode RNA silencing suppressors. Nat. Genet..

[381] Hou Y., Zhai Y., Feng L., Karimi H.Z., Rutter B.D., Zeng L., Choi D.S., Zhang B., Gu W., Chen X. (2019). A *Phytophthora* effector suppresses trans-kingdom RNAi to promote disease susceptibility. Cell Host Microbe.

[382] Dunoyer P., Himber C., Voinnet O. (2006). Induction, suppression and requirement of RNA silencing pathways in virulent *Agrobacterium tumefaciens* infections. Nat. Genet..

[383] Katiyar-Agarwal S., Gao S., Vivian-Smith A., Jin H. (2007). A novel class of bacteria-induced small RNAs in *Arabidopsis*. Genes Dev..

[384] Katiyar-Agarwal S., Morgan R., Dahlbeck D., Borsani O., Villegas A., Zhu J.-K., Staskawicz B.J., Jin H. (2006). A pathogen-inducible endogenous siRNA in plant immunity. Proc. Natl. Acad. Sci. USA.

[385] Wagh S.G., Alam M.M., Kobayashi K., Yaeno T., Yamaoka N., Toriba T., Hirano H.Y., Nishiguchi M. (2016). Analysis of rice RNA-dependent RNA polymerase 6 (*OsRDR6*) gene in response to viral, bacterial and fungal pathogens. J. Gen. Plant Pathol..

[386] Cai Q., Qiao L., Wang M., He B., Lin F.M., Palmquist J., Huang S.D., Jin H. (2018). Plants send small RNAs in extracellular vesicles to fungal pathogen to silence virulence genes. Science.

[387] Hudzik C., Hou Y., Ma W., Axtell M.J. (2020). Exchange of small regulatory RNAs between plants and their pests. Plant Physiol..

[388] Wang B., Sun Y., Song N., Zhao M., Liu R., Feng H., Wang X., Kang Z. (2017). *Puccinia striiformis* f. sp. *tritici* microRNA-like RNA 1 (*Pst*-milR1), an important pathogenicity factor of *Pst*, impairs wheat resistance to *Pst* by suppressing the wheat pathogenesis-related 2 gene. New Phytol..

[389] Kusch S., Frantzeskakis L., Thieron H., Panstruga R. (2018). Small RNAs from cereal powdery mildew pathogens may target host plant genes. Fungal Biol..

[390] Nunes C.C., Dean R.A. (2012). Host-induced gene silencing: A tool for understanding fungal host interaction and for developing novel disease control strategies. Mol. Plant Pathol..

[391] Knip M., Constantin M.E., Thordal-Christensen H. (2014). Trans-kingdom cross-talk: Small RNAs on the move. PLoS Genet..

[392] Wang M., Jin H. (2017). Spray-induced gene silencing: A powerful innovative strategy for crop protection. Trends Microbiol..

[393] Wang M., Thomas N., Jin H. (2017). Cross-kingdom RNA trafficking and environmental RNAi for powerful innovative pre-and post-harvest plant protection. Curr. Opin. Plant Biol..

[394] Zhang T., Zhao Y.L., Zhao J.H., Wang S., Jin Y., Chen Z.Q., Fang Y.Y., Hua C.L., Ding S.W., Guo H.S. (2016). Cotton plants export microRNAs to inhibit virulence gene expression in a fungal pathogen. Nat. Plants.

[395] Huang G., Dong R., Allen R., Davis E.L., Baum T.J., Hussey R.S. (2006). A root-knot nematode secretory peptide functions as a ligand for a plant transcription factor. Mol. Plant Microbe Interact..

[396] Nowara D., Gay A., Lacomme C., Shaw J., Ridout C., Douchkov D., Hensel G., Kumlehn J., Schweizer P. (2010). HIGS: Host-induced gene silencing in the obligate biotrophic fungal pathogen *Blumeria graminis*. Plant Cell.

[397] Baulcombe D.C. (2015). VIGS, HIGS and FIGS: Small RNA silencing in the interactions of viruses or filamentous organisms with their plant hosts. Curr. Opin. Plant Biol..

[398] Kuck K.H., Stenzel K., Vors J.P., Krämer W., Schirmer U., Jeschke P., Witschel M. (2012). Sterol biosynthesis inhibitors. Modern Crop Protection Compounds.

[399] Koch A., Kumar N., Weber L., Keller H., Imani J., Kogel K.H. (2013). Host-induced gene silencing of cytochrome P450 lanosterol C14α-demethylase–encoding genes confers strong resistance to *Fusarium* species. Proc. Natl. Acad. Sci. USA.

[400] Cheng W., Song X.S., Li H.P., Cao L.H., Sun K., Qiu X.L., Xu Y.B., Yang P., Huang T., Zhang J.B. (2015). Host-induced gene silencing of an essential chitin synthase gene confers durable resistance to *Fusarium* head blight and seedling blight in wheat. Plant Biotechn. J..

[401] Panwar V., Jordan M., McCallum B., Bakkeren G. (2018). Host-induced silencing of essential genes in *Puccinia triticina* through transgenic expression of RNAi sequences reduces severity of leaf rust infection in wheat. Plant Biotechnol. J..

[402] Qi T., Zhu X., Tan C., Liu P., Guo J., Kang Z., Guo J. (2018). Host-induced gene silencing of an important pathogenicity factor *PsCPK1* in *Puccinia striiformis* f. sp. *tritici* enhances resistance of wheat to stripe rust. Plant Biotechnol. J..

[403] Chen W., Kastner C., Nowara D., Oliveira-Garcia E., Rutten T., Zhao Y., Deising H.B., Kumlehn J., Schweizer P. (2016). Host-induced silencing of *Fusarium culmorum* genes protects wheat from infection. J. Exp. Bot..

[404] Liu Y., Schiff M., Dinesh-Kumar S.P. (2002). Virus-induced gene silencing in tomato. Plant J..

[405] Ghag S.B. (2017). Host induced gene silencing, an emerging science to engineer crop resistance against harmful plant pathogens. Physiol. Mol. Plant Pathol..

[406] Tomilov A.A., Tomilova N.B., Wroblewski T., Michelmore R., Yoder J.I. (2008). Trans-specific gene silencing between host and parasitic plants. Plant J..

[407] Burch-Smith T.M., Anderson J.C., Martin G.B., Dinesh-Kumar S.P. (2004). Applications and advantages of virus-induced gene silencing for gene function studies in plants. Plant J..

[408] Scofield S.R., Huang L., Brandt A.S., Gill B.S. (2005). Development of a virus-induced gene-silencing system for hexaploid wheat and its use in functional analysis of the *Lr21*-mediated leaf rust resistance pathway. Plant Physiol..

[409] Cooper B., Campbell K.B. (2017). Protection against common bean rust conferred by a gene-silencing method. Phytopathology.

[410] Hnatuszko-Konka K., Kowalczyk T., Gerszberg A., Wiktorek-Smagur A., Kononowicz A.K. (2014). *Phaseolus vulgaris*—Recalcitrant potential. Biotechnol. Adv..

[411] Zhang C., Bradshaw J.D., Whitham S.A., Hill J.H. (2010). The development of an efficient multipurpose bean pod mottle virus viral vector set for foreign gene expression and RNA silencing. Plant Physiol..

[412] Ranjan A., Jayaraman D., Grau C., Hill J.H., Whitham S.A., Ané J.M., Smith D.L., Kabbage M. (2018). The pathogenic development of *Sclerotinia sclerotiorum* in soybean requires specific host NADPH oxidases. Mol. Plant Pathol..

[413] Soltis N.E., Atwell S., Shi G., Fordyce R., Gwinner R., Gao D., Shafi A., Kliebenstein D.J. (2019). Interactions of tomato and *Botrytis cinerea* genetic diversity: Parsing the contributions of host differentiation, domestication, and pathogen variation. Plant Cell.

[414] Davis J., Yu D., Evans W., Gokirmak T., Chetelat R.T., Stotz H.U. (2009). Mapping of loci from *Solanum lycopersicoides* conferring resistance or susceptibility to *Botrytis cinerea* in tomato. Theor. Appl. Genet..

[415] Fu Y., Song Y., van Tuyl J.M., Visser R.G.F., Arens P. (2023). The use of a candidate gene approach to study *Botrytis cinerea* resistance in *Gerbera hybrida*. Front. Plant Sci..

[416] Su K., Zhao W., Lin H., Jiang C., Zhao Y., Guo Y. (2023). Candidate gene discovery of *Botrytis cinerea* resistance in grapevine based on QTL mapping and RNA-seq. Front. Plant Sci..

[417] Caseys C., Shi G., Soltis N., Gwinner R., Corwin J., Atwell S., Kliebenstein D.J. (2021). Quantitative interactions: The disease outcome of *Botrytis cinerea* across the plant kingdom. G3 (Bethesda).

[418] Mekapogu M., Jung J.-A., Kwon O.-K., Ahn M.-S., Song H.-Y., Jang S. (2021). Recent progress in enhancing fungal disease resistence in ornamental plants. Int. J. Mol. Sci..

[419] Xiang J., Lei X., Wu Z., Cao X., Zhang D., Teng N. (2022). An efficient and novel method to screen *Botrytis cinerea* resistance genes based on TRV-induced gene silencing with lily petal discs. Physiol. Mol. Plant Pathol..

[420] Ding C., Gao J., Zhang S., Jiang N., Su D., Huang X., Zhang Z. (2023). The basic/helix-loop-helix transcription factor family gene *RcbHLH112* is a susceptibility gene in gray mould resistance of rose (*Rosa chinensis*). Int. J. Mol. Sci..

[421] Li D., Li X., Liu X., Zhang Z. (2023). Comprehensive analysis of *bZIP* gene family and function of *RcbZIP17* on *Botrytis* resistance in rose (*Rosa chinensis*). Gene.

[422] Tian Y., Zhang S., Liu X., Zhang Z. (2021). Global investigation of TBL gene family in rose (*Rosa chinensis*) unveils *RcTBL16* is a susceptibility gene in gray mold resistance. Front. Plant Sci..

[423] Zhou J., Li N., Hu N., Tang N., Cao H., Liu Y., Chen J., Jian W., Gao Y., Yang J. (2022). Co-silencing of ABA receptors (SlRCAR) reveals interactions between ABA and ethylene signaling during tomato fruit ripening. Hortic. Res..

[424] Zhang H., Yan M., Deng R., Song F., Jiang M. (2020). The silencing of DEK reduced disease resistance against *Botrytis cinerea* and *Pseudomonas syringae* pv. *tomato* DC3000 based on virus-induced gene silencing analysis in tomato. Gene.

[425] Zhang H., Yin L., Song F., Jiang M. (2020). SKIP silencing decreased disease resistance against *Botrytis cinerea* and *Pseudomonas syringae* pv. *tomato* DC3000 in tomato. Front. Plant Sci..

[426] Wang D., Chen L., Liu C., Wang H., Liu Z., Ji X., He N., Xin Y. (2024). Mno-miR164a and *MnNAC100* regulate the resistance of mulberry to *Botrytis cinerea*. Physiol. Plant..

[427] Li Z., Yang S., Ma Y., Sui Y., Xing H., Zhang W., Liao Q., Jiang Y. (2023). Molecular mechanism of miR160d in regulating kiwifruit resistance to *Botrytis cinerea*. J. Agric. Food. Chem..

[428] Lee M.B., Han H., Lee S. (2023). The role of WRKY transcription factors, FaWRKY29 and FaWRKY64, for regulating *Botrytis* fruit rot resistance in strawberry (*Fragaria* × *ananassa* Duch.). BMC Plant Biol..

[429] Wang Y., Zhao F., Zhang G., Jia S., Yan Z. (2021). *FaWRKY11* transcription factor positively regulates resistence to *Botrytis cinerea* in strawberry fruit. Sci. Hortic..

[430] Sun K., van Tuinen A., van Kan J.A., Wolters A.-M.A., Jacobsen E., Visser R.G.F., Bai Y. (2017). Silencing of *DND1* in potato and tomato impedes conidial germination, attachment and hyphal growth of *Botrytis cinerea*. BMC Plant Biol..

[431] Gao W., Long L., Xu L., Lindsey K., Zhang X., Zhu L. (2015). Suppression of the homeobox gene *HDTF1* enhances resistence to *Verticillium dahliae* and *Botrytis cinerea* in cotton. J. Integr. Plant Biol..

[432] Liao C.-J., Hailemariam S., Sharon A., Mengiste T. (2022). Pathogenic strategies and immune mechanisms to necrotrophs: Differences and similarities to biotrophs and hemibiotrophs. Curr. Opin. Plant Biol..

[433] Cai Q., He B., Kogel K.H., Jin H. (2018). Cross-kingdom RNA trafficking and environmental RNAi—nature’s blueprint for modern crop protection strategies. Curr. Opin. Microbiol..

[434] Villalobos-Escobedo J.M., Herrera-Estrella A., Carreras-Villaseñor N. (2016). The interaction of fungi with the environment orchestrated by RNAi. Mycologia.

[435] Whangbo J.S., Hunter C.P. (2008). Environmental RNA interference. Trends Genet..

[436] Waterhouse P.M., Wang M.B., Lough T. (2001). Gene silencing as an adaptive defence against viruses. Nature.

[437] Timmons L., Court D.L., Fire A. (2001). Ingestion of bacterially expressed dsRNAs can produce specific and potent genetic interference in *Caenorhabditis elegans*. Gene.

[438] Baum J.A., Bogaert T., Clinton W., Heck G.R., Feldmann P., Ilagan O., Johnson S., Plaetinck G., Munyikwa T., Pleau M. (2007). Control of coleopteran insect pests through RNA interference. Nat. Biotechn..

[439] Mulot M., Boissinot S., Monsion B., Rastegar M., Clavijo G., Halter D., Bochet N., Erdinger M., Brault V. (2016). Comparative analysis of RNAi-based methods to down-regulate expression of two genes expressed at different levels in *Myzus persicae*. Viruses.

[440] Koch A., Biedenkopf D., Furch A., Weber L., Rossbach O., Abdellatef E., Linicus L., Johannsmeler J., Jelonek L., Goesmann A. (2016). An RNAi-based control of *Fusarium graminearum* infections through spraying of long dsRNAs involves a plant passage and is controlled by the fungal silencing machinery. PLoS Pathog..

[441] Worrall E.A., Bravo-Cazar A., Nilon A.T., Fletcher S.J., Robinson K.E., Carr J.P., Mitter N. (2019). Exogenous application of RNAi-inducing double-stranded RNA inhibits aphid-mediated transmission of a plant virus. Front. Plant Sci..

[442] Sang H., Kim J.I. (2000). Advanced strategies to control plant pathogenic fungi by host-induced gene silencing (HIGS) and spray-induced gene silencing (SIGS). Plant Biotechnol. Rep..

[443] Asokan R., Sharath Chandra G., Manamohan M., Krishna Kumar N.K., Sita T. (2014). Response of various target genes to diet-delivered dsRNA mediated RNA interference in the cotton bollworm, *Helicoverpa armigera*. J. Pest Sci..

[444] Meyering-Vos M., Müller A. (2007). RNA interference suggests sulfakinins as satiety effectors in the cricket *Gryllus bimaculatus*. J. Insect Physiol..

[445] Štefanić S., Dvořák J., Horn M., Braschi S., Sojka D., Ruelas D.S., Brian Suzuki B., Lim K.C., Hopkins S.D., McKerrow J.H. (2010). RNA interference in *Schistosoma mansoni* schistosomula: Selectivity, sensitivity and operation for larger-scale screening. PLoS Negl. Trop. Dis..

[446] Dornseifer S., Willkomm S., Far R.K.K., Liebschwager J., Beltsiou F., Frank K., Laufer S.D., Martinetz T., Sczakiel G., Claussen J.C. (2015). RNAi revised-target mRNA-dependent enhancement of gene silencing. Nucleic Acids Res..

[447] Larsson E., Sander C., Marks D. (2010). mRNA turnover rate limits siRNA and microRNA efficacy. Mol. Syst. Biol..

[448] Tenllado F., Díaz-Ruíz J.R. (2001). Double-stranded RNA-mediated interference with plant virus infection. J. Virol..

[449] Tenllado F., Martínez-García B., Vargas M., Díaz-Ruíz J.R. (2003). Crude extracts of bacterially expressed dsRNA can be used to protect plants against virus infections. BMC Biotechnol..

[450] Duanis-Assaf D., Galsurker O., Davydov O., Maurer D., Feygenberg O., Sagi M., Poverenov E., Fluhr R., Alkan N. (2022). Double-stranded RNA targeting fungal ergosterol biosynthesis pathway controls *Botrytis cinerea* and postharvest grey mould. Plant Biotechnol. J..

[451] Duanis-Assaf D., Shlar I., Galsurker O., Davydov O., Maurer D., Feygenberg O., Poverenov E., Fluhr R., Alkan N. (2022). Nano-clay, layered-double hydroxide (LDH), improves the efficacy of double-stranded RNA in controlling postharvest decay. Postharvest Biol. Technol..

[452] Niño-Sánchez J., Chen L.H., de Souza J.T., Mosquera S., Stergiopoulos I. (2021). Targeted delivery of gene silencing in fungi using genetically engineered bacteria. J. Fungi.

[453] Choquer M., Fournier E., Kunz C., Levis C., Pradier J.M., Simon A., Viaud M. (2007). *Botrytis cinerea* virulence factors: New insights into a necrotrophic and polyphageous pathogen. FEMS Microbiol. Lett..

[454] Blanco-Ulate B., Morales-Cruz A., Amrine K.C., Labavitch J.M., Powell A.L., Cantu D. (2014). Genome-wide transcriptional profiling of *Botrytis cinerea* genes targeting plant cell walls during infections of different hosts. Front. Plant Sci..

[455] Breen J., Mur L.A.J., Sivakumaran A., Akinyemi A., Wilkinson M.J., Rodriguez Lopez C.M. (2022). *Botrytis cinerea* loss and restoration of virulence during in vitro culture follows flux in global DNA methylation. Int. J. Mol. Sci..

[456] Spada M., Viviani A., Pugliesi C., Fambrini M., Pecchia S. (2021). Spray application of *BcBmp3*-dsRNA delivered by layered double hydroxide (LDH) clay nanosheets reduces virulence of *Botrytis cinerea* on *Lactuca sativa*: First results. J. Plant Pathol..

[457] Lück S., Kreszies T., Strickert M., Schweizer P., Kuhlmann M., Douchkov D. (2019). siRNA-Finder (si-Fi) software for RNAi-target design and off-target prediction. Front. Plant Sci..

[458] Fletcher S.J., Reeves P.T., Hoang B.T., Mitter N. (2020). A perspective on RNAi-based biopesticides. Front. Plant Sci..

[459] Broekaert W.F., Terras F.R., Cammue B.P., Vanderleyden J. (1990). An automated quantitative assay for fungal growth inhibition. FEMS Microbiol. Lett..

[460] Koch A., Stein E., Kogel K.H. (2018). RNA-based disease control as a complementary measure to fight *Fusarium* fungi through silencing of the azole target Cytochrome P450 Lanosterol C-14 α-Demethylase. Eur. J. Plant Pathol..

[461] Šečić E., Kogel K.H. (2021). Requirements for fungal uptake of dsRNA and gene silencing in RNAi-based crop protection strategies. Curr. Opin. Biotechnol..

[462] Shim C.K., Kim M.J., Kim Y.K., Jee H.J. (2014). Evaluation of lettuce germplasm resistance to gray mold disease for organic cultivations. Plant Pathol. J..

[463] Sarkar A., Roy-Barman S. (2021). Spray-induced silencing of pathogenicity gene *MoDES1* via exogenous double-stranded RNA can confer partial resistance against fungal blast in rice. Front. Plant Sci..

[464] Song X.S., Gu K.X., Duan X.X., Xiao X.M., Hou Y.P., Duan Y.B., Wang J.X., Zhou M.G. (2018). A myosin5 dsRNA that reduces the fungicide resistance and pathogenicity of *Fusarium asiaticum*. Pest. Biochem. Physiol..

[465] Yan S., Qian J., Cai C., Ma Z., Li J., Yin M., Ren B., Shen J. (2020). Spray method application of transdermal dsRNA delivery system for efficient gene silencing and pest control on soybean aphid *Aphis glycines*. J. Pest Sci..

[466] Hough J., Howard J.D., Brown S., Portwood D.E., Kilby P.M., Dickman M.J. (2020). Strategies for production of dsRNA biocontrols as alternatives to chemical pesticides. Front. Bioeng. Biotechnol..

[467] Rosa S., Pesaresi P., Mizzotti C., Bulone V., Mezzetti B., Baraldi E., Masiero S. (2022). Game-changing alternatives to conventional fungicides: Small RNAs and short peptides. Trends Biotechnol..

[468] Mann C.W.G., Sawyer A., Garbiner D.M., Mitter N., Carroll B.J., Eamens A.L. (2023). RNA-based control of fungal pathogens in plants. Int. J. Mol. Sci..

[469] Degnan R.M., Shuey L.S., Radford-Smith J., Gardiner D.M., Cattoll B.J., Mitter N., McTaggart A.R., Sawyer A. (2023). Double-stranded RNA prevents and cures infection by rust fungi. Commun. Biol..

[470] Singewar K., Fladung M. (2023). Doubled-stranded RNA (dsRNA) technology to control forest insect pests and fungal pathogens: Challenges and opportunities. Funct. Integr. Genom..

[471] Chen A., Halilovic L., Shay J.H., Koch A., Mitter N., Jin H. (2023). Improving RNA-based crop protection through nanotechnology and insights from cross-kingdom RNA trafficking. Curr. Opin. Plant Biol..

[472] Ray P., Sahu D., Aminedi R., Chandran D. (2022). Concepts and considerations for enhancing RNAi efficiency in phytopathogenic fungi for RNAi-based crop protection using nanocarrier-mediated dsRNA delivery systems. Front. Fungal Biol..

[473] Huang L., Jin J., Deighan P., Kiner E., McReynolds L., Lieberman J. (2013). Efficient and specific gene knockdown by small interfering RNAs produced in bacteria. Nat. Biotechnol..

[474] Yin G., Sun Z., Liu N., Zhang L., Song Y., Zhu C., Wen F. (2009). Production of double-stranded RNA for interference with TMV infection utilizing a bacterial prokaryotic expression system. Appl. Microbiol. Biotechnol..

[475] Ahn S.J., Donahue K., Koh Y., Martin R.R., Choi M.Y. (2019). Microbial-based double-stranded RNA production to develop cost-effective RNA interference application for insect pest management. Int. J. Insect Sci..

[476] Beaucage S.L., Reese C.B. (2009). Recent advances in the chemical synthesis of RNA. Curr. Protoc. Nucleic Acid Chem..

[477] Ahmadzada T., Reid G., McKenzie D.R. (2018). Fundamentals of siRNA and miRNA therapeutics and a review of targeted nanoparticle delivery systems in breast cancer. Biophys. Rev..

[478] Álvarez-Sánchez A.R., Romo-Quinones C., Rosas-Quijano R., Reyes A.G., Barraza A., Magallón-Barajas F., Angulo C., Mejía-Ruíz C.H. (2018). Production of specific dsRNA against white spot syndrome virus in the yeast *Yarrowia lipolytica*. Aquac. Res..

[479] Voloudakis A.E., Holeva M.C., Sarin L.P., Bamford D.H., Vargas M., Poranen M.M., Tenllado F. (2015). Efficient double-stranded RNA production methods for utilization in plant virus control. Plant Virology Protocols: New Approaches to Detect Viruses and Host Responses.

[480] Taning C.N.T., Arpaia S., Christiaens O., Dietz-Pfeilstetter A., Jones H., Mezzetti B., Sabbadini S., Sorteberg H., Sweet J., Ventura V. (2020). RNA-based biocontrol compounds: Current status and perspectives to reach the market. Pest Manag. Sci..

[481] Taning C.N.T., Mezzetti B., Kleter G., Smagghe G., Baraldi E. (2021). Does RNAi-based technology fit within EU sustainability goals?. Trends Biotechnol..

[482] Wuthisathid K., Chaijarasphong T., Chotwiwatthanakun C., Somrit M., Sritunyalucksana K., Itsathitphaisarn O. (2021). Co-expression of double-stranded RNA and viral capsid protein in the novel engineered *Escherichia coli* DualX-B15(DE3) strain. BMC Microbiol..

[483] Newmark P.A., Reddien P.W., Cebria F., Alvarado A.S. (2003). Ingestion of bacterially expressed double-stranded RNA inhibits gene expression in planarians. Proc. Natl. Acad. Sci. USA.

[484] Tian H., Peng H., Yao Q., Chen H., Xie Q., Tang B., Zhang W. (2009). Developmental control of a lepidopteran pest *Spodoptera exigua* by ingestion of bacteria expressing dsRNA of a non-midgut gene. PLoS ONE.

[485] Mendelsohn M.L., Gathmann A., Kardassi D., Sachana M., Hopwood E.M., Dietz-Pfeilstetter A., Michelsen-Correa S., Fletcher S.J., Székács A. (2020). Summary of discussions from the 2019 OECD conference on RNAi based pesticides. Front. Plant Sci..

[486] Somchai P., Jitrakorn S., Thitamadee S., Meetam M., Saksmerprome V. (2016). Use of microalgae *Chlamydomonas reinhardtii* for production of double-stranded RNA against shrimp virus. Aquacult. Rep..

[487] Guan R., Chu D., Han X., Miao X., Li H. (2021). Advances in the development of microbial double-stranded RNA production systems for application of RNA interference in agricultural pest control. Front. Bioeng. Biotechnol..

[488] Figueiredo Prates L.H., Merlau M., Rühl-Teichner J., Schetelig M.F., Häcker I. (2023). An optimized/scale up-ready protocol for extraction of bacterially produced dsRNA at good yield and low costs. Int. J. Mol. Sci..

[489] Nwokeoji A.O., Nwokeoji E.A., Chou T., Togola A. (2022). A novel sustainable platform for scaled manufacturing of double-stranded RNA biopesticides. Bioresour. Bioprocess..

[490] Aalto A.P., Sarin L.P., Van Dijk A.A., Saarma M., Poranen M.M., Arumäe U., Bamford D.H. (2007). Large-scale production of dsRNA and siRNA pools for RNA interference utilizing bacteriophage ϕ6 RNA-dependent RNA polymerase. RNA.

[491] Niehl A., Soininen M., Poranen M.M., Heinlein M. (2018). Synthetic biology approach for plant protection using ds RNA. Plant Biotech. J..

[492] Sun Y., Qiao X., Mindich L. (2004). Construction of carrier state viruses with partial genomes of the segmented dsRNA bacteriophages. Virology.

[493] Lichtenberg S.S., Nuti K., DeRouchey J., Tsyusko O.V., Unrine J.M. (2020). Efficacy of chitosan/double-stranded RNA polyplex nanoparticles for gene silencing under variable environmental conditions. Env. Sci. Nano.

[494] Wang K., Peng Y., Chen J., Peng Y., Wang X., Shen Z., Han Z. (2020). Comparison of efficacy of RNAi mediated by various nanoparticles in the rice striped stem borer (*Chilo suppressalis*). Pestic. Biochem. Physiol..

[495] Scarpin D., Nerva L., Chitarra W., Moffa M., D’Este F., Vuerich M., Filippi A., Braidot E., Petrussa E. (2023). Characterisation and functionalisation of chitosan nanoparticles as carriers for double-stranded RNA (dsRNA) molecules towards sustainable crop protection. Biosci. Rep..

[496] Wang Y., Yan Q., Lan C., Tang T., Wang K., Shen J., Niu D. (2023). Nanoparticle carriers enhance RNA stability and uptake efficiency and prolong the protection against *Rhizoctonia solani*. Phytopathol. Res..

[497] Mitter N., Worrall E.A., Robinson K.E., Li P., Jain R.G., Taochy C., Fletcher S.J., Carroll B.J., Lu G., Xu Z.P. (2017). Clay nanosheets for topical delivery of RNAi for sustained protection against plant viruses. Nat. Plants.

[498] Niño-Sánchez J., Sambasivam P.T., Sawyer A., Hamby R., Chen A., Czislowski E., Li P., Manzie N., Gardiner D.M., Ford R. (2022). BioClay™ prolongs RNA interference-mediated crop protection against *Botrytis cinerea*. J. Integr. Plant Biol..

[499] Qiao L., Niño-Sánchez J., Hamby R., Capriotti L., Chen A., Mezzetti B., Jin H. (2023). Artificial nanovesicles for dsRNA delivery in spray induced gene silencing for crop protection. Plant Biotechnol. J..

[500] Meagher R.B., Lewis Z.A., Ambati S., Lin X. (2021). Aiming for the bull’s eye: Targeting antifungals to fungi with dectin-decorated liposomes. PLoS Pathog..

[501] Voltan A.R., Quindos G., Alarcón K.P.M., Fusco-Almeida A.M., Mendes-Giannini M.J.S., Chorilli M. (2016). Fungal diseases: Could nanostructured drug delivery systems be a novel paradigm for therapy?. Int. J. Nanomed..

[502] Qiao L., Lan C., Capriotti L., Ah-Fong A., Nino Sanchez J., Hamby R., Heller J., Zhao H., Glass N.L., Judelson H.S. (2021). Spray-induced gene silencing for disease control is dependent on the efficiency of pathogen RNA uptake. Plant Biotechnol. J..

[503] Arpaia S., Christiaens O., Giddings K., Jones H., Mezzetti B., Moronta-Barrios F., Perry J.N., Sweet J.B., Taning C.N.T., Smagghe G. (2020). Biosafety of GM crop plants expressing dsRNA: Data requirements and EU regulatory considerations. Front. Plant Sci..

[504] Gu K.X., Song X.S., Xiao X.M., Duan X.X., Wang J.X., Duan Y.B., Hou Y.P., Zhou M.G. (2019). A *β_2_-tubulin* dsRNA derived from *Fusarium asiaticum* confers plant resistance to multiple phytopathogens and reduces fungicide resistance. Pestic. Biochem. Physiol..

[505] Xiong F., Liu M., Zhuo F., Yin H., Deng K., Feng S., Liu Y., Luo X., Feng L., Zhang S. (2019). Host-induced gene silencing of *BcTOR* in *Botrytis cinerea* enhances plant resistance to grey mould. Mol. Plant Pathol..

[506] Petrick J.S., Brower-Toland B., Jackson A.L., Kier L.D. (2013). Safety assessment of food and feed from biotechnology-derived crops employing RNA-mediated gene regulation to achieve desired traits: A scientific review. Regul. Toxicol. Pharmacol..

[507] Rodrigues T.B., Petrick J.S. (2020). Safety considerations for humans and other vertebrates regarding agricultural uses of externally applied RNA molecules. Front. Plant Sci..

[508] Vaishnaw A.K., Gollob J., Gamba-Vitalo C., Hutabarat R., Sah D., Meyers R., de Fougerolles T., Maraganore J. (2010). A status report on RNAi therapeutics. Silence.

[509] Dalakouras A., Koidou V., Papadopoulou K. (2024). DsRNA-based pesticides: Considerations for efficiency and risk assessment. Chemosphere.

[510] Arazoe T. (2021). CRISPR-based pathogenic fungal genome editing for control of infection and disease. Prog. Mol. Biol. Transl. Sci..

[511] Paul N.C., Park S.-W., Liu H., Choi S., Ma J., MacCready J.S., Chilvers M.I., Sang H. (2021). Plant and fungal genome editing to enhance plant disease resistance using the CRISPR/Cas9 system. Front. Plant Sci..

[512] Singh R., Caseys C., Kliebenstein D.J. (2024). Genetic and molecular landscapes of the generalist phytopathogen *Botrytis cinerea*. Mol. Plant Pathol..

[513] Zhang T., Jin Y., Zhao J.H., Gao F., Zhou B.J., Fang Y.Y., Guo H.S. (2016). Host-induced gene silencing of the target gene in fungal cells confers effective resistance to the cotton wilt disease pathogen *Verticillium dahliae*. Mol. Plant.

[514] de Oliveira Filho J.G., Silva G.D.C., Cipriano L., Gomes M., Egea M.B. (2021). Control of postharvest fungal diseases in fruits using external application of RNAi. J. Food Sci..

[515] Rank A.P., Koch A. (2021). Lab-to-field transition of RNA spray applications—How far are we?. Front. Plant Sci..

[516] Rabuma T., Gupta O.P., Chhokar V. (2022). Recent advances and potential applications of cross-kingdom movement of miRNAs in modulating plant’s disease response. RNA Biol..

[517] Koeppe S., Kawchuk L., Kalischuk M. (2023). RNA interference past and future applications in plants. Int. J. Mol. Sci..

[518] Padilla-Roji I., Ruiz-Jiménez L., Bakhat N., Vielba-Fernández A., Pérez-García A., Fernández-Ortuño D. (2023). RNAi technology: A new path for the research and management of obligate biotrophic phytopathogenic fungi. Int. J. Mol. Sci..

[519] Zhang B., Shao L., Wang J., Zhang Y., Guo X., Peng Y., Cao Y., Lai Z. (2021). Phosphorylation of ATG18a by BAK1 suppresses autophagy and attenuates plant resistance against necrotrophic pathogens. Autophagy.

[520] Finiti I., de la O Leyva M., Vicedo B., Gómez-Pastor R., López-Cruz J., García-Agustín P., Real M.D., González-Bosch C. (2014). Hexanoic acid protects tomato plants against *Botrytis cinerea* by priming defence responses and reducing oxidative stress. Mol. Plant Pathol..

[521] Jia Y., Kang L., Wu Y., Zhou C., Cai R., Zhang H., Li J., Chen Z., Kang D., Zhang L. (2024). Nano-selenium foliar intervention-induced resistance of cucumber to *Botrytis cinerea* by activating jasmonic acid biosynthesis and regulating phenolic acid and cucurbitacin. Pest. Manag. Sci..

[522] An Y.-Q., Bi B.-S., Xu H., Xi Z. (2023). Co-application of brassinolide and pyraclostrobin improved disease control efficacy by eliciting plant innate defense responses in *Arabidopsis thaliana*. J. Agric. Food Chem..

[523] Li Y., Li S., Du R., Wang J., Li H., Xie D., Yan J. (2021). Isoleucine enhances plant resistance against *Botrytis cinerea* via jasmonate signaling pathway. Front. Plant Sci..

[524] Zhao L., Islam M.S., Song P., Zhu L., Dong W. (2023). Isolation and optimization of a broad-spectrum synthetic antimicrobial peptide, Ap920-WI, from *Arthrobacter* sp. H5 for the biological control of plant diseases. Int. J. Mol. Sci..

[525] Li R., Cheng Y. (2023). Recent advances in mechanisms underlying defense responses of horticultural crops to *Botrytis cinerea*. Int. J. Mol. Sci..

[526] Orsi B., Sestari I., Preczenhak A.P., de Abreu Vieira A.P., Tessmer M.A., da Silva Souza M.A., Hassimotto N.M.A., Kluge R.A. (2023). Fruits tomato carotenoid mutants have altered susceptibility, to grey mold. Plant Physiol. Biochem..

[527] Maruri-López I., Romero-Contreras Y.J., Napsucialy-Mendivil S., González-Pérez E., Aviles-Baltazar N.Y., Chávez-Martínez A.I., Flores-Cuevas E.J., Schwan-Estrada K.R.F., Dubrovsky J.G., Jiménez-Bremont J.F. (2024). A biostimulant yeast, *Hanseniaspora opuntiae*, modifies *Arabidopsis thaliana* root architecture and improves the plant defense response against *Botrytis cinerea*. Planta.

[528] Yong J., Wu M., Carroll B.J., Xu Z.P., Zhang R. (2024). Enhancing plant biotechnology by nanoparticle delivery of nucleic acids. Trends Genet..

[529] Mezzetti B., Smagghe G., Arpaia S., Christiaens O., Dietz-Pfeilstetter A., Jones H., Kostov K., Sabbadini S., Opsahl-Sorteberg H.-G., Ventura V. (2020). RNAi: What is its position in agriculture?. J. Pest Sci..

